# Comparative proteomics reveals abnormal binding of ATGL and dysferlin on lipid droplets from pressure overload-induced dysfunctional rat hearts

**DOI:** 10.1038/srep19782

**Published:** 2016-01-22

**Authors:** Linghai Li, Huina Zhang, Weiyi Wang, Yun Hong, Jifeng Wang, Shuyan Zhang, Shimeng Xu, Qingbo Shu, Juanfen Li, Fuquan Yang, Min Zheng, Zongjie Qian, Pingsheng Liu

**Affiliations:** 1 Department of Anesthesiology, Beijing Chest Hospital, Capital Medical University, Beijing Tuberculosis and Thoracic Tumor Research Institute, Beijing, China; 2 Beijing An Zhen Hospital, Capital Medical University, Key Laboratory of Upper Airway Dysfunction-related Cardiovascular Diseases, Beijing Institute of Heart Lung and Blood Vessel Disease, Beijing, China; 3 National Laboratory of Biomacromolecules, Institute of Biophysics, Chinese Academy of Sciences, Beijing, China; 4 Department of Cardiovascular Diseases, Civil Aviation General Hospital, Peking University, Beijing, China; 5 Department of Gastroenterology, the First Affiliated Hospital, College of Medicine, Zhejang University, Hangzhou, China; 6 University of Chinese Academy of Sciences, Beijing, China; 7 Department of Cardiology, Affiliated Hospital of Guilin Medical University, Guilin, China

## Abstract

Excessive retention of neutral lipids in cardiac lipid droplets (LDs) is a common observation in cardiomyopathy. Thus, the systematic investigation of the cardiac LD proteome will help to dissect the underlying mechanisms linking cardiac steatosis and myocardial dysfunction. Here, after isolation of LDs from normal and dysfunctional Sprague-Dawley rat hearts, we identified 752 heart-associated LD proteins using iTRAQ quantitative proteomic method, including 451 proteins previously unreported on LDs. The most noteworthy finding was the identification of the membrane resealing protein, dysferlin. An analysis of dysferlin truncation mutants indicated that its C2 domain was responsible for its LD localization. Quantitative proteomic results further determined that 27 proteins were increased and 16 proteins were decreased in LDs from post pressure overload-induced dysfunctional hearts, compared with normal hearts. Notably, adipose triacylglycerol lipase (ATGL) was dramatically decreased and dysferlin was substantially increased on dysfunctional cardiac LDs. This study for the first time reveals the dataset of the heart LD proteome in healthy tissue and the variation of it under cardiac dysfunction. These findings highlight an association between the altered LD protein localization of dysferlin and ATGL and myocardial dysfunction.

The heart is a major consumer of energy through lipid utilization^1^. However, under certain pathological conditions associated with cardiac dysfunction, excess neutral lipids are deposited in cardiomyocytes as the consequence of insufficient fatty acid β-oxidation^2^,^3^,^4^. Lipid droplets (LDs), a ubiquitous organelle distributed among most cell types, serve as a neutral lipid reservoir and provide fatty acids to fuel cellular β-oxidative processes^5^. LDs stringently govern the storage and turnover of intracellular neutral lipids through the actions of LD-associated proteins, including both lipid metabolic enzymes as well as LD structural proteins of the perilipin family (PLINs)^6^. The altered expression and activity of these LD-associated proteins are reported to influence cardiac lipid homeostasis and, subsequently, cardiac function^7^. For instance, the cardiac targeted overexpression of adipose triacylglycerol lipase (ATGL) protects against pressure overload-induced cardiac dysfunction^8^, ameliorates diabetes-induced cardiomyopathy^9^, and even prevents obesity-related cardiac steatosis and dilated cardiomyopathy^10^. Thus, obtaining a global view of the cardiac LD protein profile under different physiological and pathological conditions will help to extend our understanding of heart lipid metabolism and the underlying mechanisms maintaining cardiac lipid homeostasis as well as provide insight into etiology of various cardiac pathological states.

Besides their role in neutral lipid metabolism^11^, LDs are also involved in diverse intracellular processes including signal transduction^12^, protein storage^13^, and membrane trafficking^14^ through the mediation of LD proteins either embedded in or associated with the organelle. Accumulated LD proteomic results suggest that proteins associated with membrane repair such as the SNARE complex^15^, Caveolin-3^16^, Rab proteins^17^, MG-53/TRIM72^18^, and dysferlin^19^ are located on LDs^20^,^21^. Other evidence also points to a potential relationship between membrane repair and lipid metabolism. For example, the membrane traffic inhibitor BFA not only blocks membrane repair^22^, but also blocks intracellular neutral lipid storage^23^. Moreover, a deficiency of dysferlin, a key protein in membrane repair, induces aberrant TAG accumulation^24^. It is well established that membrane repair proteins play a pivotal role in sustaining normal cardiac function, since rapid and efficient membrane resealing is crucial for maintaining cardiac plasma membrane integrity as well as normal cardiac contraction and relaxation^16^. However, the relationship between membrane sealing and lipid metabolism in cardiomyocytes remains obscure and needs further investigation. Therefore, the examination of the heart LD proteome will provide clues to illuminate the role of the organelle in cardiac membrane repair, and to dissect the mechanisms linking lipid metabolism, membrane repair, and cardiac function.

In this study, we investigated cardiac LD proteome in normal and pressure overload-induced dysfunctional rar heart. 752 proteins were identified. Of these, 43 proteins were found with significant variation in heart LD under different conditions. These findings provide useful information for future studies regarding the functions of heart LDs and give some novel clues to promote the development of clinical treatments for cardiopathy.

## Results

### Morphology of lipid droplets in rat myocardium

**Figure 1:**
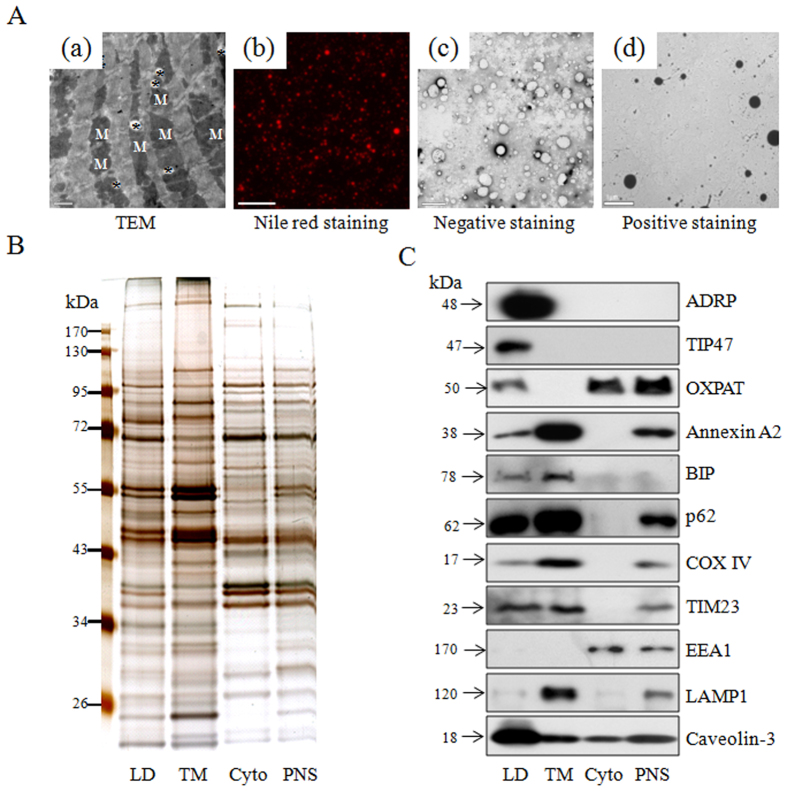
Isolating LDs from rat heart. (**Aa**) Ultra-thin sections of rat heart were observed by TEM. Asterisks indicate LDs. M, mitochondrial. Bar = 1 μm. (**Ab**) LD fraction isolated from rat heart was stained with Nile red and imaged by fluorescence microscopy. Bar = 50 μm, Heart LDs were imaged by TEM after negative staining (**Ac**) or positive staining (Ad). Bar = 1 μm. (**B**) Equal amount of proteins of different cellular fractions were separated by SDS-PAGE followed by silver staining. TM, total membrane; Cyto, cytosol; PNS, post-nuclear supernatant. (**C**) Equal amount of proteins from isolated LDs, total membrane (TM), cytosol (Cyto), and post-nuclear supernatant (PNS) were processed for immunoblotting with indicated antibodies.

Transmission electron microscopy (TEM) observation of mature rat heart revealed that cardiac LDs were dispersed in cardiomyocytes and were tightly associated with mitochondria (Fig. 1Aa). Heart LDs were isolated from five rat hearts according to a modified protocol, as reported previously^21^. Nile red-stained fluorescence micrographs showed that heart LDs appeared spherical shape. Apart from a few large LDs, most isolated LDs were smaller than 1 μm in diameter (Fig. 1Ab). Consistently, the electron micrographs from both negative and positive staining modes revealed the integrity of isolated cardiac LDs with most of them <1 μm in diameter (Fig. 1Ac,Ad). In addition to the morphological evaluation, the purity of the isolated LDs was also determined using more stringent biochemical measurements. Results from silver staining of electrophoretically separated proteins demonstrated that the protein pattern from the isolated LDs was distinctly different from that of post-nuclear supernatant (PNS), total membranes (TM) and cytosol (Cyto), suggesting the significant enrichment of LD-specific proteins (Fig. 1B). The purity of LDs was further assessed by the relative abundance of LD-resident proteins including adipocyte differentiation related protein (ADRP/PLIN2), TIP47/PLIN3, OXPAT/PLIN5, and by the deficiency of proteins of other intracellular compartments (i.e. EEA1, endosome; LAMP1, lysosome). The cell membrane protein Annexin A2 and muscle specific caveola protein caveolin-3 were also found in the LD fraction, consistent with previous observations^21^. Some mitochondrial proteins (COX IV and TIM23) and ER proteins (BIP and p62) were also found in the LD fraction (Fig. 1C), indicating the LD fraction isolated from rat heart was largely free of other organelles except for ER and mitochondria, which was in agreement with the proposed argument that LD was originally from ER^25^ and frequently associated with mitochondria^21^.

### Normal and dysfunctional rat heart lipid droplets

**Figure 2:**
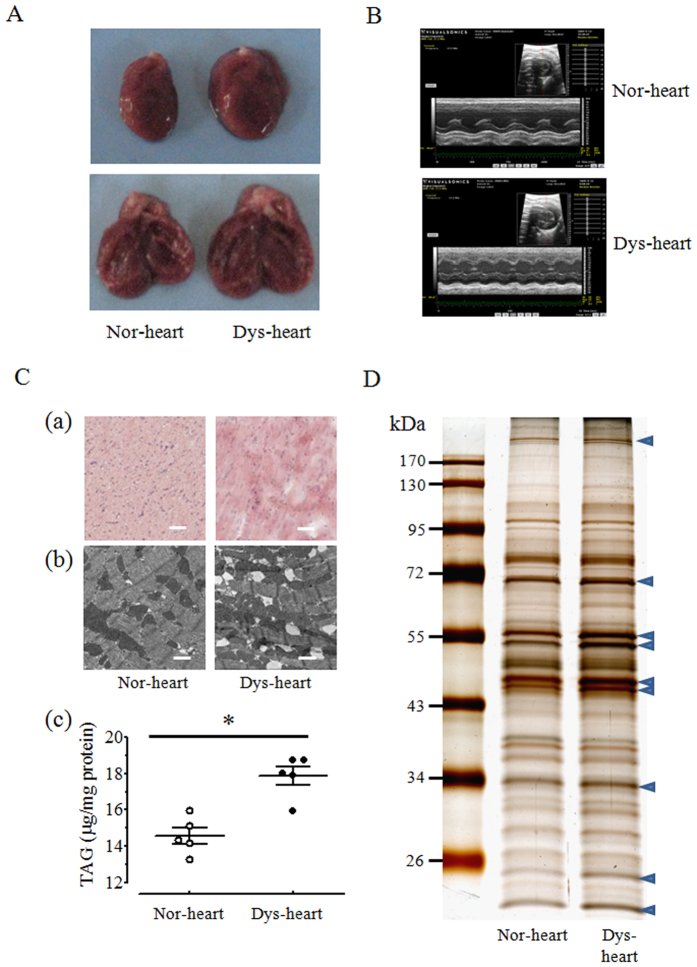
Morphology and protein profile of normal and dysfunctional rat heart LDs. (**A**) The morphological images of representative normal and dysfunctional heart with integral (left) or longitudinal section view (right). (**B**) Cardiac function of normal (upper) and dysfunctional heart (lower) was detected by echocardiography assay. (**C**) The tissue morphology of normal and dysfunctional heart was observed by microscope after Oil Red O staining (a, Bar = 20 μm) or by TEM (b, Bar = 1 μm). The corresponding TAG content was quantified by the relative ratio of TAG to protein (c). Results are means ± SEM (n = 5). *p < 0.05 vs. Nor-heart. (**D**) Different protein pattern of normal and dysfunctional cardiac LD. Arrows indicated the higher intensity protein bands in dysfunctional cardiac samples. Nor-heart, normal heart; Dys-heart, dysfunctional heart.

The establishment of a method to isolated cardiac LDs allowed us to compare the LD protein composition between normal and dysfunctional hearts to reveal molecular details associated with disorders of cardiomyocyte lipid storage and metabolism. We first compared the histological morphology of normal and dysfunctional hearts after successfully establishing the pressure overload-induced dysfunctional heart model in rats (Fig. 2A,B). Images taken of heart ventricle cryo-sections demonstrated increased lipid accumulation in dysfunctional heart with stronger Oil red O (ORO) staining than normal tissue (Fig. 2Ca). Furthermore, TEM images clearly showed that LDs in dysfunctional myocardium were larger and more abundant compared with the normal group (Fig. 2Cb). Consistent with the microscopy, a semi-quantitative analysis of triacylglycerol demonstrated that approximately 25% more of the neutral lipid deposited in dysfunctional hearts than the normal ones (Fig. 2Cc). The significant differences in LD morphology suggested that there may be accompanying distinctions in the protein profiles between these two groups. To examine this, the proteins extracted from normal and dysfunctional heart LDs were silver stained after electrophoretic separation on polyacrylamide gels. Indeed, some differences were observed in the LD protein profiles from the two groups; notably, some proteins bands in the gels from the dysfunctional group had substantially stronger staining than the controls (Fig. 2D, blue arrows).

### Quantitative proteomic analysis of cardiac lipid droplets

**Figure 3:**
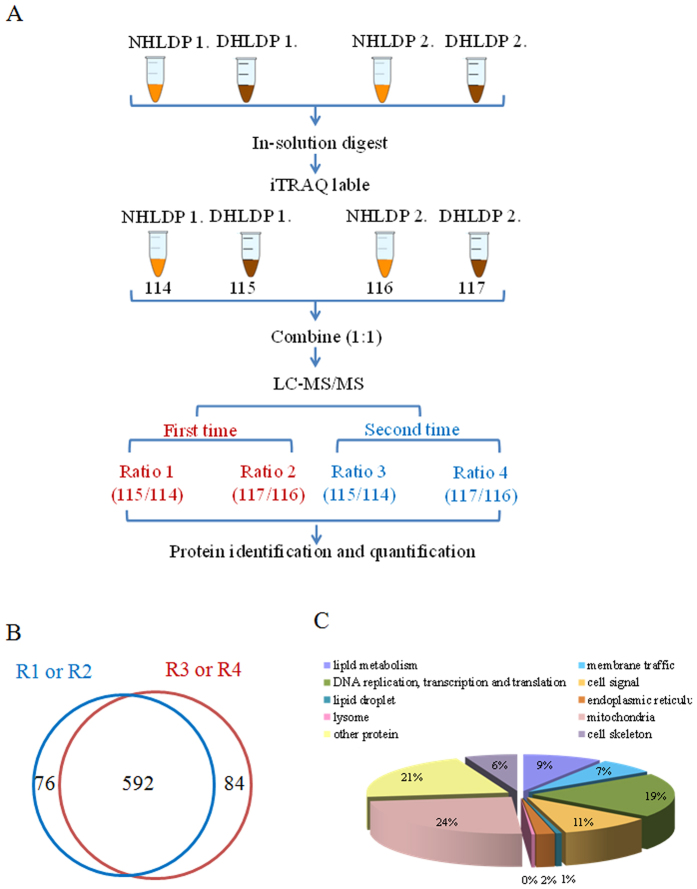
Proteome analysis of isolated heart LD. (**A**) Flowchart showed the experiment design for heart LD comparative proteomics. (**B**) The Venn diagram showed the overlap of the identified LD proteins in two technical replicates (blue and red) and two biological replicates (115/114 and 117/116). (**C**) The identified heart LD proteins were categorized by sub-cellular distributions and functions based on PANTHER and Uniprot KB sources.

After validating the purity and protein profile of LDs isolated from normal and dysfunctional hearts, quantitative proteomic analysis was performed. The proteins extracted from normal and dysfunctional heart LDs were digested with trypsin, labeled by iTRAQ, and then analyzed by nano LC-MS/MS (Fig. 3A). To obtain reliable results, two biological replicates and two technical replicates were carried out. One biological group contained proteins from normal cardiac LDs (labeled with iTRAQ reagents, 114 or 116) and dysfunctional cardiac LDs (labeled with iTRAQ reagents, 115 or 117). Only one protein (Isoform 4 of NADH dehydrogenase) was identified without any iTRAQ labeling. Therefore, we set our acceptance criteria to include proteins with at least two unique labeled peptides (Fig. 3A). Under this criterion, we identified a total of 752 rat cardiac LD-associated proteins after combining four replicates (R1-R4; 688, 688, 680 and 680 proteins respectively). Of these, 592 proteins were reproducibly detected as shown in the Venn diagram (Fig. 3B), with a subset of 301 proteins having been reported previously (Supplementary, Table S1), indicating the accuracy of the LD purification and proteomics techniques.

All identified proteins were categorized into 10 groups according to their function and their assigned sub-cellular locations based on PATRHE and Uniprot KB websites (Fig. 3C and Supplementary, Table S1). Examining these groups, mitochondrial proteins had the greatest representation (24%, 185 proteins), which was also observed in our previous LD proteomics from skeletal muscle^21^, and brown adipose tissue^26^, suggesting the intimate metabolic relationship between LDs and mitochondria in cardiomyocytes. Another major group was proteins involved in DNA replication, transcription and protein translation and modification (about 20%, 153 proteins), including several histone proteins and 21 chaperones. This finding raised the possibility that LDs may be involved in intracellular protein expression and activity by modulating protein maturation or degradation. Some recent study indeed proved that LD played a role in histone storage and stabilization^27^, and also participated in the degradation of ApoB100 and 3-hydroxy-3-methylglutaryl CoA (HMG CoA) through the mediation of LD-related Derlin-1 and Ubxd8^28^. Roughly 11% of the proteome from heart LD samples was cell signaling proteins (83 proteins), supporting the hypothesis that LDs participate in intracellular signal transduction. Another notable group was composed of 71 lipid metabolic proteins (about 9% of the total), including the enzymes implicated in metabolism of triacylglycerol (TAG), fatty acids, phospholipids, and sterol esters. This was in line with the role of LDs as a lipid metabolic compartment to sustain the intracellular lipid homeostasis. Membrane trafficking proteins accounted for approximately 6% of the total proteins. Besides the previously reported LD-related Rab proteins, SNAREs and coatomers^29^,^30^,^31^, the most unexpected finding was the identification of the muscle-specific membrane-sealing protein dysferlin and its associated proteins, MG-53/TRIM72^32^, caveolin-3^33^ and Annexin A2^34^. These identifications imply that cardiomyocyte LDs may be participated in membrane repairing during the constant process of myocardial contraction and relaxation. Besides the abundant mitochondrial proteins, the heart LD proteome also included proteins from other organelles and intracellular structures such as ER and the cytoskeleton, which is consistent with a dynamic interaction between LDs and other intracellular compartments. Finally, there were a substantial number of proteins (158; 21% of the total) not belonging to the other 9 categories, indicating some functions of LDs are still unknown and will require further study.

The STRING web database was employed to analyze the physical and functional interactions of all the identified LD proteins. Among the total 752 identified proteins, 671 had entries in the STRING database with 6173 associations as depicted by specific colored lines (Supplementary Fig. S1). Examining only the lipid metabolic and PLIN family proteins, the database depicted 57 proteins and presented 146 associations (Supplementary Fig. S2). The protein association networks enabled us to analyze the identified proteins systematically. From this complicated global view, some proteins showed close relationship with each other and formed unique protein clusters. Consistent with our functional classification, the densest interaction networks contained lipid metabolic proteins (orange color), and mitochondrial proteins (yellow color). Three other notable interaction networks were composed of cytoskeletal proteins (green color), membrane trafficking proteins (red color), and proteins involved in DNA replication, transcription, protein translation, and modification (blue color). On the other hand, the protein clusters can also lead insights into unknown protein functions and to help deeply understanding the relationship between proteins. Taking three enzymes in TAG mobilization as example, besides the internal connection with each other, there were specific linkage belonging to each enzyme, such as the interaction of Pnpla2 (ATGL) with Abhd5 (CGI-58); Lipe (HSL) with apolipoproteins (Apob and Apoe) and LDL receptor (Ldlr); and Mgll (MGL) with Lss (lanosterol synthase) (Supplementary Fig. S2), suggesting that except for the role in TAG hydrolysis that the three enzymes deal with together, there are unique functions owned by each of them.

### Verification of proteomic results

**Figure 4:**
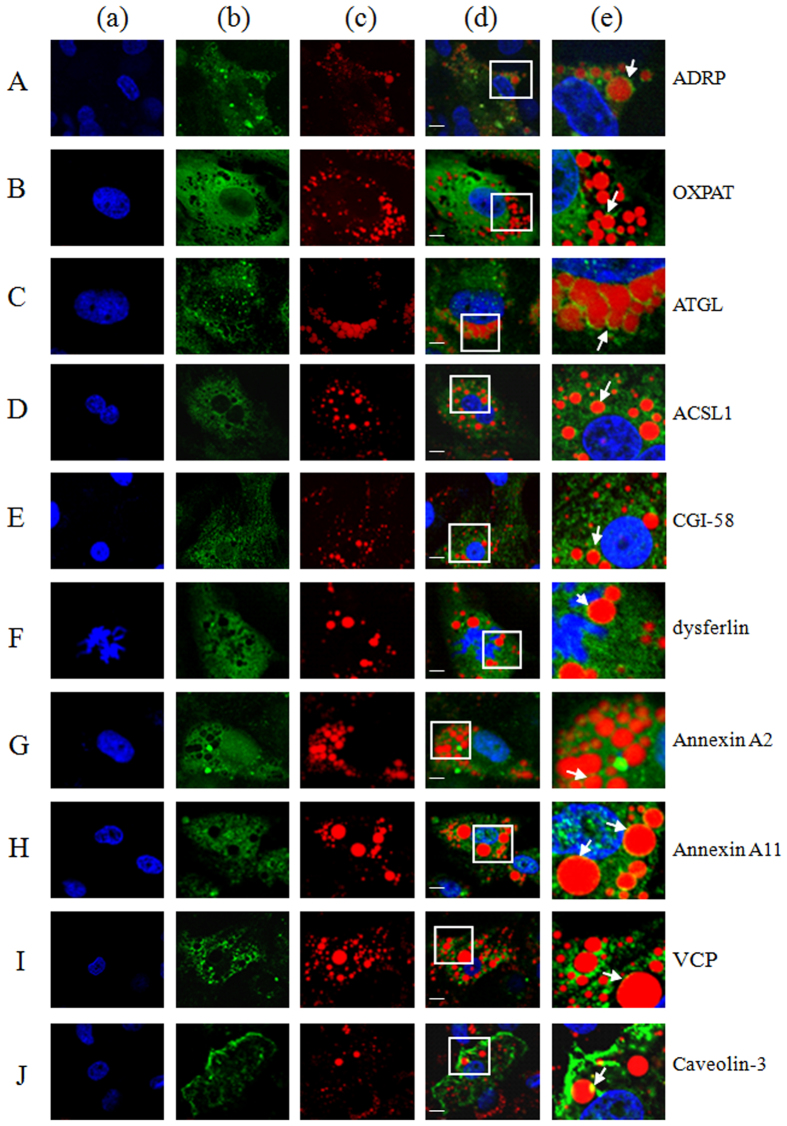
Subcellular distribution of LD-associated proteins in primary cardiomyocytes. The primary cardiomyocytes isolated from neonatal rat were cultured in DMEM supplemented with 10% FBS. 100 μmol/L oleate was added in the medium for 24 hours before the immunostaining analysis. (**A–J**) Ten of the identified heart LD proteins including ADRP, OXPAT, ATGL, ACSL, CGI-58, dysferlin, Annexin A2, Annexin A11, VCP, caveolin-3 were detected by immunofluorescent assay. Column a (blue): nucleus stained with Hochest33258; column b (green): LD-associated proteins stained with FITC; column c (red): LD stained with LipidTOX Deep Red; column d: the corresponding merged picture of column a, b and c; column e: the enlarged image of the white frame from column d pictures. The white arrowheads represented the LD residence of indicated proteins. Bar = 5 μm.

To verify the proteomic results, immunofluorescence assays were carried out to detect some of the identified LD-associated proteins in oleate-treated primary cardiomyocytes. The perilipin family protein ADRP/PLIN2 surrounded LDs, forming a ring-shaped image (Fig. 4A), while OXPAT/PLIN5 was present on the LDs as well as in the cytoplasm (Fig. 4B), implying the diverse characteristics of these two proteins in lipid metabolism and in maintaining the LD structure. Lipid metabolic proteins such as ATGL, ACSL and CGI-58 were also observed both on the LDs and in the cytoplasm (Fig. 4C–E). As expected, dysferlin (Fig. 4F) and dysferlin-associated Annexin A2, Annexin A11, caveolin-3, and VCP (Fig. 4G–J) were observed partially localized on cardiomyocyte LDs. But no green signal was observed in negative control images (Supplementary Figure S3).

### Lipid droplet targeting domain of dysferlin and overexpression of dysferlin decreases TAG accumulation

**Figure 5:**
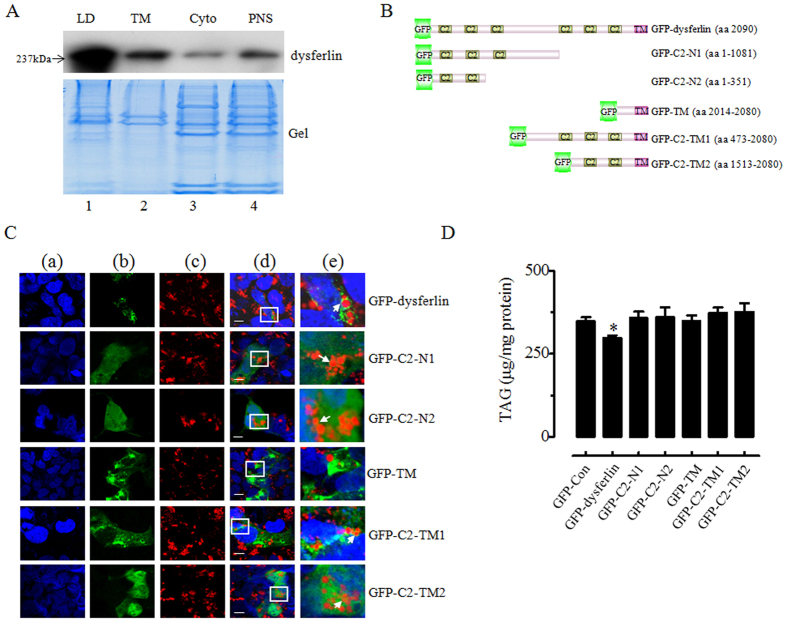
C2 domain-dependent LD targeting of dysferlin and attenuated TAG accumulation by dysferlin. (**A**) Sub-cellular distribution of dysferlin was detected by immunoblotting assay. Colloidal Blue-staining gel was utilized to present protein loading. (**B**) Domain diagram of full-length and truncation mutant constructs of dysferlin with GFP in-frame fusion in the C-terminal. (**C**) A series of truncation mutants of dysferlin expression vectors were transiently transfected to HEK293A along with oleate treatment for 24 hours and observed by an Olympus FV1000. Column a (blue): nuclear stained with Hochest33258; column b (green): GFP; column c (red): LD stained with LipidTOX Deep Red; column d: the corresponding merged picture of column a, b and c; column e: the enlarged image of the white frame from column d pictures. The white arrowheads represented the LD localization of indicated proteins. Bar = 5 μm. (**D**) TAG content was quantified by the relative ratio of TAG to protein after transient transfection of dysferlin and a series of truncation mutants of dysferlin expression vectors to HEK293A cells along with oleate (100 μmol/L) treatment for 24 hours. Results are means ± SEM (n = 4). *p < 0.05 vs. GFP-Con.

Immunoblotting assays were performed to confirm the localization of dysferlin. As shown in Fig. 5A, dysferlin was dramatically enriched on LDs rather than total membrane. Dysferlin consists of 6 cytosolic C2 domains (C2A–F) and a single-pass transmembrane domain at its C terminus^35^ (Fig. 5B). The C2 domains of dysferlin are thought to function in membrane repair, vesicle trafficking, and membrane fusion^36^ by interaction with phospholipids and with other proteins. Therefore, we hypothesized that the C2 domains may be involved in the LD targeting of dysferlin. To test this hypothesis, a series of dysferlin C2 domain truncation mutants fused with a GFP reporter were over-expressed in oleate-treated HEK293A cells. Fluorescence confocal micrographs showed that although different truncations had distinctive intracellular locations (e.g., GFP-C2-TM1 and GFP-TM had more cytoplasmic signal, whereas GFP-C2-N2, GFP-C2-N1 and GFP-C2-TM2 signals were equally dispersed throughout the cytoplasm and nucleus), if the C2 domain was present in the structure, there was still merged fluorescence (yellow) signal observed on the LDs. This indicates that the C2 domain, not transmembrane domain, was responsible for targeting dysferlin to the surface of LDs (Fig. 5C). In addition, the peptide sequences identified for dysferlin in the LD proteome included many amino acids belonging to C2 domain (Supplementary Fig. S4), although not completely identical (which may because the hydrophobic amino acids of C2 domains^37^ are not easy to be identified by mass spectrometry), giving another support for the pivotal role of C2 domain in dysferlin LD targeting. Additionally, to determine the relationship between dysferlin and lipid storage, WT dysferlin and its truncation mutants were transiently transfected into HEK293A with the similar efficiency, alone with oleate treatment for 24 hours, and then TAG level was measured. Results showed that only WT dysferlin attenuated TAG amount after overexpression in HEK293A (Fig. 5D).

### Differential protein expression on lipid droplets from normal and dysfunctional hearts

**Figure 6:**
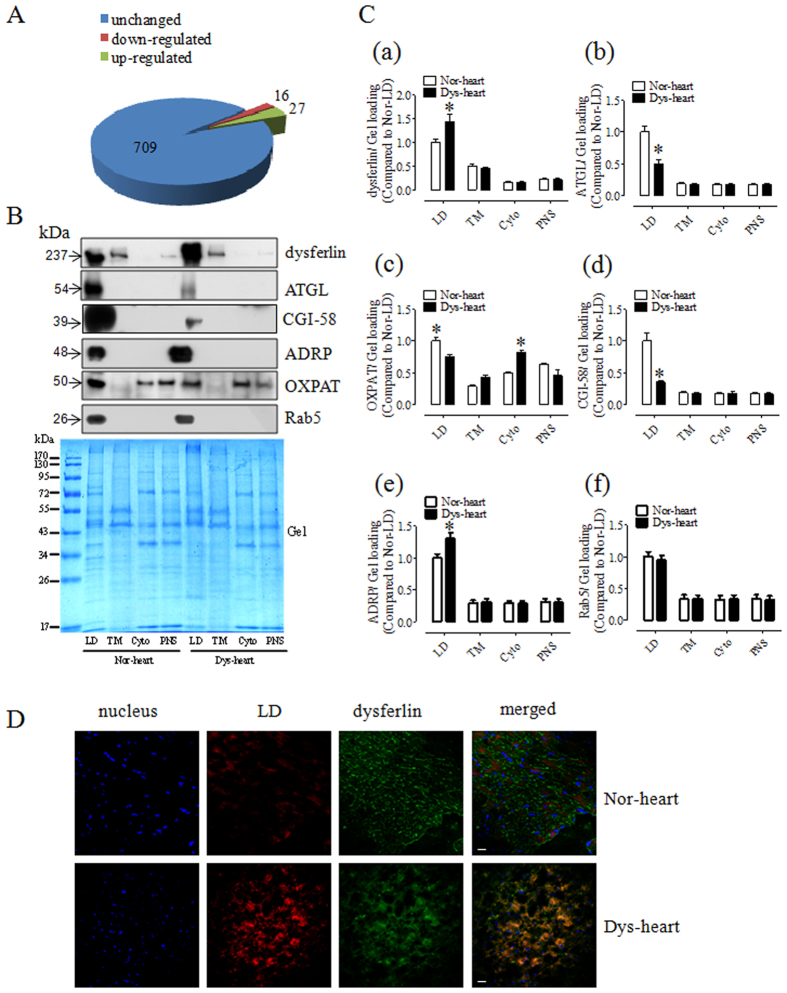
Comparative proteomics of the normal and dysfunctional heart LD. (**A**) The Venn diagram showed the number of unchanged, up-regulated and down-regulated proteins from cardiac LD comparative proteome. (**B**) After iTRAQ-quantification, some of the identified proteins including perilipin family proteins (ADRP, OXPAT), membrane traffic proteins (dysferlin, Rab 5) and metabolic proteins (ATGL, CGI-58) were detected in different cellular fractions by Western blotting. (**C**) Statistical data of the expression of Dysferlin, ATGL, OXPAT, CGI-58, ADRP and Rab 5 on normal and dysfunctional cardiac LDs. Protein expression was normalized to gel staining. Results are means ± SEM (n = 4). *p < 0.05 vs. Nor-heart. (**D**) Immunofluorescence results demonstrated the localization of dysferln in cryosection of normal and dysfunctional hearts. Blue, nuclear stained with Hochest33258; green: dysferlin stained with FITC; red: LD stained with LipidTOX Deep Red. Bar = 20 μm.

**Figure 7:**
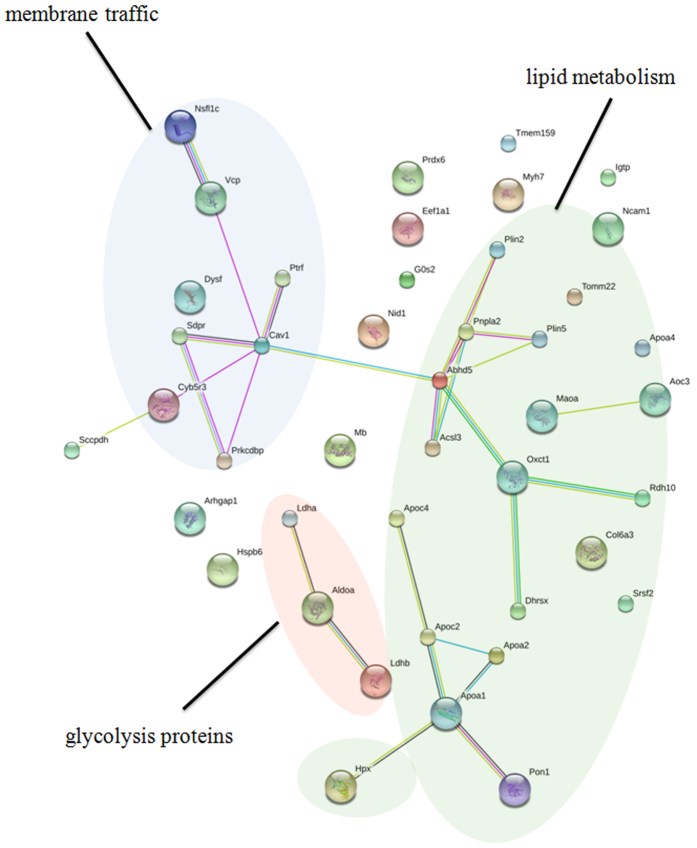
The association network of LD proteins with varying expression in normal and dysfunctional heart. The relationship of heart LD proteins with different expression under physical and pathological status was predicted by the website program STRING against rat database. Network edges represent predicted functional associations with different line colors standing for various types of evidence used for predicting: red, fusion evidence; green, neighborhood evidence; blue, co-occurrence evidence; purple, experimental evidence; yellow, text-mining evidence; black, co-expression evidence. Purple circle embraced the proteins associated with membrane traffic process; red circle comprised the glycolysis-related proteins and green circle contained the lipid metabolism-related proteins.

Using iTRAQ-based quantitative proteomics with a Q Exactive mass spectrometer (Thermo Scientific, USA) equipped with an Easy n-LC 1,000 HPLC (Thermo Scientific, USA), we were not only able to identify more LD-associated proteins than previously reported, but also able to obtain quantification data for comparative proteomics. To obtain reliable quantification results, only proteins quantified at least twice (iTRAQ labeled twice) were accepted. Since the expression ratio of most proteins was unchanged and the mean value of the total protein expression ratio was 1.033 with standard deviation (SD) value of 0.225, a 1.96-fold change was chosen as the threshold for significant changes in protein expression. Using these criteria, the expression levels of 43 proteins were changed in dysfunctional cardiac LDs compared with normal ones. Of these, 16 proteins were down-regulated and 27 proteins were up-regulated (Fig. 6A, Table 1). Supplementary Figure S5 in presented a representative MS/MS spectrum of the dysferlin peptide with the different expression level in normal and dysfunctional cardiac LD samples after iTRAQ labeling. Consistent with iTRAQ quantification results, Western blotting validation showed that ADRP/PLIN2 were up-regulated, whereas the ATGL, CGI-58 as well as OXPAT/PLIN5 were reduced on dysfunctional cardiac LDs (Fig. 6B,C), suggesting that the excessive accumulation of lipid in dysfunctional hearts was directly due to the lower hydrolysis of TAG. Notably, dysferlin and dysferlin-associated partners VCP, caveolin-3, and Annexin A2 were enriched on dysfunctional cardiac LDs compared with normal LDs (Fig. 6B,C and Supplementary Fig. S6). Consistent with the immunoblotting results, immunostaining demonstrated that more dysferlin signal was apparent in the cytoplasm of dysfunctional cardiac myocytes in comparison to normal ones (Fig. 6D). The enrichment of these proteins on LDs may be related to the insufficient membrane healing known to exist in dysfunctional hearts. Meanwhile, in accordance with the comparative proteome identification, Western blotting demonstrated that there is no difference in Rab5 targeting on dysfunctional cardiac LDs and its counterpart (Fig. 6B,C). As to the identified proteins with altered expression level on heart LDs under different physiological and pathological statuses, 43 proteins entered STRING database and 60 links were illustrated (Fig. 7). In the STRING network, membrane traffic proteins (purple), lipid metabolism proteins (green) and glycolysis proteins (red) formed 3 main protein links that revealed the relevant alterations of these intracellular processes between normal and dysfunctional hearts.

## Discussion

In the research presented here, we have enumerated the proteome of cardiac LDs and have made a comparative analysis between normal and dysfunction hearts. We identified 752 LD proteins by 2D-LC-MS/MS, including 451 previously unreported proteins. Dysferlin was found closely associated with LDs in cardiomyocytes and its C2 domain was found to be required for LD targeting. A quantitative proteomic comparison between normal and dysfunctional heart LDs identified 43 proteins with altered expression levels in LDs. Notably, this suggests that the reduction of ATGL and the enrichment of dysferlin on the defective cardiac LDs may be relevant to the development of cardiac dysfunction.

Membrane repair is an elemental process for maintaining cell integrity and function. Cardiomyocytes in particular require unique mechanisms to efficiently and effectively repair the plasma membrane damaged due to frequent disruptions caused by persistent mechanical contraction. Transient membrane damage existing in normal cardiomyocytes is exacerbated by various pathophysiological stresses when the capacity of membrane repair machinery cannot meet the cellular requirement. Dysferlin is a well-recognized membrane-associated protein that is implicated in the repair of sarcolemma membrane in skeletal muscles^38^. Recent studies have found that dysferlin-deficient mice display defective cardiomyocyte membrane repair capacity that is linked to increased susceptibility to cardiomyopathy^39^,^40^. It has been postulated that dysferlin exerts its membrane repair function by binding with an unidentified lysosomal-exclusive vesicular compartment^39^. However, the nature of these vesicles and the exact mechanism underlying dysferlin-mediated membrane repair remain to be elucidated^41^. Our results showed that dysferlin and its partners, VCP, Annexin A2, and caveolin-3, were localized on heart LDs, implying that LDs contribute to cardiac membrane resealing. Truncation mutants revealed that the phospholipid binding structure, the C2 domain, may be responsible for dysferlin LD targeting. Moreover, the identification of the dysferlin-interacting cardiac membrane-repair proteins, MG-53/TRIM72^32^ and its partner PTRF^42^ in the heart LD proteome further supports the hypothesis that LDs are involved in membrane repair. Beyond our current observations, there is precedence in previous research also pointing to a role for LDs in membrane resealing through the synthesis of phospholipids (phospholipids synthases localized on LD^21^), or by providing a source of pre-existing membrane lipids either following homotypic fusion^43^, or direct fusion with the plasma membrane at the disrupted location^44^,^30^, However, there is no direct experimental evidence in support of this and no molecular mechanism has been demonstrated. The more interesting finding is the decreased TAG accumulation by dysferlin overexpression in HEK293A cells, while the increased expression of dysferlin and its associated proteins Annexin A2 and caveolin-3 on dysfunctional cardiac LDs according to the comparative proteomics study. These observations let us hypothesize that dysferlin and its partners possibly promote the lipid conversion from TAG to phospholipid under normal situation, thus decreasing the accumulation of intracellular TAG when overexpressing dysferin. While under cardiac dysfunction, LDs may only act as a docking site of these membrane carrier proteins since energy generation is insufficient for membrane-resealing and lipid conversion.

Synthesis and catabolism of intracellular TAG are tightly controlled under normal conditions through regulation of the expression, location and activity of lipid metabolic enzymes. Under circumstances where lipid storage and energy utilization are unbalanced, the pathologic accumulation of TAG in LDs can occur. For instance, under normal conditions, lipotropic hormones promote CGI-58 translocation to LDs, leading to the recruitment of ATGL, and a subsequent enhancement of ATGL activity hydrolyzing TAG. At the same time, increased phosphorylation of PLIN1 through lipotropic hormone-induced pathways releases hormone sensitive lipase (HSL), triggering its relocation from the cytoplasm to LDs for its action^45^. However, in a diabetic state, elevated TNF-α reduces PLIN1 expression in adipocytes, attenuating the activity of HSL, and resulting in abnormal neutral lipid stored in adipocyte LDs^46^. Compared with other tissues such as liver and adipose, the homeostasis of lipid metabolism in the heart is more critical due to the extremely high rate of lipid turnover in the tissue. In the cardiac LD proteome presented here, we found that the TAG metabolic enzyme, ATGL, and its co-factor CGI-58 were associated with LDs. More strikingly, our comparative proteomics and subsequent verification demonstrated that the amount of ATGL and CGI-58 on LDs was reduced under cardiac dysfunction. Another significant finding from the comparative proteome data is the enhanced targeting of G0/G1 switch gene 2 (G0S2) on dysfunctional cardiac LDs. G0S2 can inhibit ATGL activity, and this suggests another mechanism explaining ATGL attenuation in cardiac dysfunction^47^. All these findings are reasonable since the insufficient β-oxidation does not require so much fatty acid hydrolyzed by ATGL under cardiac dysfunction, thus leading to the excess retention of TAG in cardiac LDs. Our Western blotting results also revealed the increased binding of ADRP/PLIN2 on dysfunctional cardiac LDs, which may be required to stabilize the enlarged LDs. By contrast, another PLIN family protein OXPAT/PLIN5 was significantly reduced in the dysfunctional cardiac LD fraction, perhaps reflecting unique functions of OXPAT/PLIN5 in the modulation of myocardial TAG deposition^48^. Collectively, the discovery of these alterations in lipid metabolism-related proteins on dysfunctional cardiac LDs will facilitate the development of a deeper understanding of the changes in lipid metabolism associated with various physiological and pathological situations.

Our study provides the first dataset of heart LD proteins as well as their variation under a pathological condition. It highlights potential associations between the ectopic LD location of dysferlin and ATGL with cardiac dysfunction. These results offer new insights into the intricate metabolic functions in heart and may promote new directions in future research concerning the etiology and treatment of heart failure. Meanwhile, some unresolved problems of this study are also proposed. For example, the exact mechanism of elevated dysferlin on defective cardiac LD still remains elusive. To obtain the dynamics and comprehensive cardiac LD proteomics, other situation such as myocardial ischemia, cardiomyopathy, and different stages of pressure overload-induced defective heart should be measured to help deeply understanding the lipid metabolism of heart under various situations.

## Methods

### Generation of rat cardiac dysfunction model

This investigation was approved by the Animal Care and Use Committee of Institute of Biophysics, Chinese Academy of Sciences, Beijing, China, which has a permission of conducting animal experiments, SYXK (SPF 2009-111). All experimental protocols were conformed to the Guide for the Care and Use of Laboratory Animals (NIH Publication Eighth Edition, updated 2011). The rat cardiac dysfunction model was constructed by clipping the ascending aorta under anesthesia by intraperitoneal injection of pentobarbital (40 mg/kg). During the operation procedure, cardiovascular and respiratory effects and variable responses were monitored. To minimize the pain, morphine (10 mg/kg, subcutaneous injection) was administered after surgery. Heart function was evaluated by echocardiography assay (Andover, MA, USA). The rats with heart ejection fraction (EF) ratio <50% were categorized into the heart dysfunction group and were sacrificed for LD isolation.

### LD isolation

Rats were sacrificed under anesthesia with i.p. pentobarbital (40 mg/kg). Five rat hearts were pooled for each LD isolation performed as described^21^.

### Protein digestion, iTRAQ labeling, LC-MS/MS analysis, protein identification and quantification analysis

The samples for iTRAQ quantitative analysis were prepared according to iTRAQ^™^ Reagents Protocol (Applied Biosystems, USA). The labeled proteins were analyzed by nanoLC-MS/MS using a Q Exactive equipped with an Easyn-LC 1000 HPLC system (Thermo Scientific). The raw data and protein quantification data were analyzed with Proteome Discovery version 1.4. The fold change threshold for up or down regulation was set as mean ± 1.960σ.

### Statistical analysis

Results represent means ± SEM (n = 4–5), unless specified. Student’s t-test (two-tailed) was used when two groups of means were compared. Protein expression was quantified by Quantity One software (Bio-Rad) and normalized to gel staining. TAG concentration was normalized to corresponding protein level. Statistical significance was accepted at *p* ≤ 0.05 (*).

## Additional Information

**How to cite this article**: Li, L. *et al*. Comparative proteomics reveals abnormal binding of ATGL and dysferlin on lipid droplets from pressure overload-induced dysfunctional rat hearts. *Sci. Rep.* **6**, 19782; doi: 10.1038/srep19782 (2016).

## Supplementary Material

Supplementary Information

## Figures and Tables

**Table 1:** The different LD proteins in normal and dysfunctional heart identified by quantitative mass spectrometry.

UniProt KB	Description	Gene Symbol	iTRAQ ratio (dysfunctional/normal)	P value
P04638	Apolipoprotein A-II	Apoa2	0.36 ± 0.07	1.20E-05
P41350	Caveolin-1	Cav1	0.47 ± 0.10	9.10E-06
G3V8L9	Polymerase I and transcript release factor	Ptrf	0.51 ± 0.05	8.25E-07
P04639	Apolipoprotein A-I	Apoa1	0.51 ± 0.02	1.00E-09
P02651	Apolipoprotein A-IV	Apoa4	0.57 ± 0.01	8.33E-05
Q75Q41	Mitochondrial import receptor subunit TOM22 homolog	Tomm22	0.57 ± 0.03	1.81E-08
D4A115	Protein Col6a3	Col6a3	0.57 ± 0.02	1.57E-05
Q6QA69	1-acylglycerol-3-phosphate O-acyltransferase ABHD5	Abhd5 (CGI-58)	0.58 ± 0.07	1.00E-07
Q66H98	Serum deprivation-response protein	Sdpr	0.58 ± 0.05	1.06E-07
G3V8D4	Apolipoprotein C-II (Predicted)	Apoc2	0.58 ± 0.04	6.26E-08
Q9Z1H9	Protein kinase C delta-binding protein	Prkcdbp	0.58 ± 0.01	8.60E-07
P0C548	Patatin-like phospholipase domain-containing protein 2	Pnpla2 (ATGL)	0.58 ± 0.05	4.79E-08
O08590	Membrane primary amine oxidase	Aoc3	0.58 ± 0.03	1.07E-07
M0R7Z9	Protein Plin5	Plin5(OXPAT)	0.58 ± 0.03	7.85E-06
F1LM84	Nidogen-1	Nid1	0.58 ± 0.03	4.06E-09
P55159	Serum paraoxonase/arylesterase 1	Pon1	0.58 ± 0.07	1.08E-03
Q5U2U5	Adipose differentiation related protein	Plin2 (ADRP)	1.48 ± 0.07	5.50E-06
P04642	L-lactate dehydrogenase A chain	Ldha	1.49 ± 0.03	1.29E-06
M0R757	Elongation factor 1-alpha	LOC100360413	1.49 ± 0.05	2.70E-03
B2GV06	Succinyl-CoA:3-ketoacid coenzyme A transferase 1, mitochondrial	Oxct1	1.49 ± 0.07	3.22E-09
G3V8B0	Myosin-7	Myh7	1.49 ± 0.10	1.33E-03
Q63151-2	Isoform Short of Long-chain-fatty-acid—CoA ligase 3	Acsl3	1.50 ± 0.07	4.95E-07
O35244	Peroxiredoxin-6	Prdx6	1.50 ± 0.09	2.61E-05
P42123	L-lactate dehydrogenase B chain	Ldhb	1.51 ± 0.05	1.17E-10
Q80ZF7	Retinol dehydrogenase 10	Rdh10	1.52 ± 0.09	1.33E-05
Q9QZ76	Myoglobin	Mb	1.52 ± 0.10	2.08E-02
P46462	Transitional endoplasmic reticulum ATPase	Vcp	1.53 ± 0.04	3.24E-10
P05065	Fructose-bisphosphate aldolase A	Aldoa	1.53 ± 0.07	1.31E-08
P55797	Apolipoprotein C-IV	Apoc4	1.58 ± 0.06	1.72E-06
P97541	Heat shock protein beta-6	Hspb6	1.58 ± 0.08	8.02E-05
P20059	Hemopexin	Hpx	1.64 ± 0.06	1.51E-08
Q6AY30	Saccharopine dehydrogenase-like oxidoreductase	Sccpdh	1.64 ± 0.09	4.98E-06
D4A6X1	Dysferlin (Predicted), isoform CRA_a	Dysf	1.64 ± 0.08	3.40E-05
Q6PDU1	Serine/arginine-rich splicing factor 2	Srsf2	1.65 ± 0.09	6.46E-06
Q6UK00	Promethin	Tmem159	1.70 ± 0.10	2.56E-09
P20070-3	Isoform 3 of NADH-cytochrome b5 reductase 3	Cyb5r3	1.72 ± 0.05	5.43E-07
F1LUV9	Neural cell adhesion molecule 1 (Fragment)	Ncam1	1.73 ± 0.01	4.98E-06
F7F469	Protein Irgm2	Igtp	1.74 ± 0.08	3.40E-05
G3V9Z3	Amine oxidase [flavin-containing] A	Maoa	1.77 ± 0.09	6.46E-06
O35987	NSFL1 cofactor p47	Nsfl1c	1.81 ± 0.05	2.56E-09
E9PTT7	Protein Dhrsx	Dhrsx	1.95 ± 0.09	1.04E-03
D4A6C5	Protein Arhgap1	Arhgap1	2.32 ± 0.04	1.47E-10
Q5M840	G0/G1 switch protein 2	G0S2	2.51 ± 0.09	8.29E-10

iTRAQ ratios was presented with mean ± SD. 95% confidence intervals (z score = 1.96) were used to determine the cutoff values for proteins with changes. Significant test for the intergroup variables were analyzed with the Student’s t-test with *p* ≤ 0.05 considered statistically significant.
