# Supplementary material for: Comparative proteomics reveals abnormal binding of ATGL and dysferlin on lipid droplets from pressure overload-induced dysfunctional rat hearts
Source: Sci Rep. 2016 Jan 22;6:19782. doi: 10.1038/srep19782 (PMC4726412; doi:10.1038/srep19782)
Supplement: Supplementary Information [file srep19782-s1.doc]

**Comparative proteomics reveals abnormal binding of ATGL and dysferlin on lipid droplets from pressure overload-induced dysfunctional rat hearts**

Linghai Li^1,3§^, Huina Zhang^2,3 §^*, Weiyi Wang^4^, Yun Hong^5^, Jifeng Wang^3^, Shuyan Zhang^3^, Shimeng Xu^3,6^, Qingbo Shu^3,6^, Juanfen Li^7^, Fuquan Yang^3^, Min Zheng^5^, Zongjie Qian^7^, and Pingsheng Liu^3^*

^1^Department of Anesthesiology, Beijing Chest Hospital, Capital Medical University,

Beijing Tuberculosis and Thoracic Tumor Research Institute, Beijing, China

^2^Beijing An Zhen Hospital, Capital Medical University, Key Laboratory of Upper Airway Dysfunction-related Cardiovascular Diseases, Beijing Institute of Heart Lung and Blood Vessel Disease, Beijing, China

^3^National Laboratory of Biomacromolecules, Institute of Biophysics, Chinese Academy of Sciences, Beijing, China

^4^Department of Cardiovascular Diseases, Civil Aviation General Hospital, Peking University, Beijing, China

^5^Department of Gastroenterology, the First Affiliated Hospital, College of Medicine, Zhejang University, Hangzhou, China

^6^University of Chinese Academy of Sciences, Beijing, China

^7^Department of Cardiology, Affiliated Hospital of Guilin Medical University, Guilin, China

^§^These authors contributed equally to this work.

*Correspondence to Pingsheng Liu and Huina Zhang

**SUPPLEMENTARY METHODS**

**Animal model**

This investigation was approved by the Animal Care and Use Committee of Institute of Biophysics, Chinese Academy of Sciences, Beijing, China, which has a permission of conducting animal experiments, SYXK (SPF 2009-111). All experimental protocols were conformed to the Guide for the Care and Use of Laboratory Animals (NIH Publication Eighth Edition, updated 2011). Four-week-old male rats (Sprague-Dawley, 70–80 g) were anesthetized with i.p. pentobarbital (40 mg/kg) and placed on a ventilator. After thoracotomy, a clip with an internal diameter of 0.58 mm (Weck Hemoclip, Research Triangle Park, NC) was placed on the ascending aorta. Animals in the sham group were treated under a similar procedure without insertion of a clip. During the operation procedure, cardiovascular and respiratory effects and variable responses were monitored. To minimize the pain, morphine (10 mg/kg, subcutaneous injection) was administered after surgery. The animals were initially randomized in two groups: one group of 30 animals with aortic banding and the other group of 32 animals with sham operation. All animals survived the initial operation

**Echocardiography assay of cardiac function**

At intervals of 18, 20 and 22 week, serial echocardiograms were performed. Rats were anesthetized with pentobarbital 40 mg/kg i.p. and the anterior chest was shaved. Transthoracic M-mode echocardiography was performed using a Hewlett-Packard Sonos 5500 imaging system (Andover, MA, USA) with a 15-MHz broadband transducer. The rats with heart ejection fraction ratio <50% were categorized as heart dysfunction group and sacrificed for LD isolation.

**Cell culture**

Rat pups (Sprague-Dawley) at the age of postnatal day 1–3 were sacriﬁced by ethyl ether. Primary rat cardiomyocytes were prepared from the cardiac ventricles of Sprague-Dawley neonates. Simply, neonate rat hearts were minced into 1 mm^3^ small pieces using small scissors and then washed 3 times with PBS to remove blood. The heart pieces were incubated in 1% trypsin (containing 0.1% collengase II) at 37°C for 15 minutes. The cell suspension was pelleted, and re-suspended in a complete medium (DMEM containing 20% fetal calf serum) and the rest of the tissue pieces were collected again and digested with trypsin for another 15 minutes. This procedure was repeated for a further 3-5 times until the heart pieces were dissolved. Then all the collected cell suspension was passed through a nylon mesh and then centrifugated at 1,000 × g for 10 minutes. The cells were re-suspended in a complete medium and plated on plastic dishes. After 2 hours, the non-adhered cells (cardiomyocytes) were aspirated in a complete medium and plated on fibronectin-coated coverslips for 12 hours before immunofluorescence detection.

The HEK293A were purchased from American Type Culture Collection and were cultured in DMEM containing 10% FBS, 100 U/mL penicillin and 100 μg/mL of streptomycin. When cells were in 90% confluent, the indicated plasmids (GFP, GFP-DFL, GFP-C2-TM1, GFP-C2-N2, GFP-C2-N1, GFP-C2-TM2 and GFP-TM) provided as gifts by Dr. Kate Bushby (Institute of Human Genetics Newcastle University) were transfected by Lipofectamine™ 2000 CD Reagent (Invitrogen, USA). All the HEK293A cells with plasmids transfection were incubated with oleate (100 μmol/L) for 24 hours before TAG level assay or confocal microscopy observation.

**LD purification**

LD was purified as previously described[^1^](#_ENREF_1) with little modification. Rats were sacrificed after anesthetizing with i.p. pentobarbital (40 mg/kg) and five rat hearts were used for once LD purification. After [anesthetization](app:ds:anesthetization) via pentobarbital, the left ventricles of rat hearts were quickly obtained from normal or dysfunctional heart SD rats and put into ice-cold PBS containing 0.2 mmol/L PMSF. Next, visible vessels and other connective tissues were carefully removed. All the heart were cut into 1～3 mm^3^ pieces and transferred to 12 mL buffer A (25 mmol/L tricine pH 7.6, 250 mmol/L sucrose) plus 0.2 mmol/L PMSF, homogenized with a glass teflon Dounce homogenizer on ice for 20 times. The heart cell suspension was prepared by passing though the 200 nylon mesh sieve with tissue homogenate. All the cells were further homogenized by N_2_ bomb (500 psi for 15 minutes on ice). After centrifugation at 3,000 × *g*, the post-nuclear supernatant (PNS) fraction (10 mL) with 2 Ml buffer B (20 mmol/L HEPES, pH 7.4, 100 mmol/L KCl, and 2 mmol/L MgCl_2_) was loaded into a SW40 tube. The sample was centrifuged at 256,000 × *g* for 1 hour at 4°C. The white band of lipid droplets floating at the top of solution was collected and washed with buffer B three times. Then the sample was ready for Nile red (Sigma-Aldrich, USA) staining or TEM observation or other following experiments.

**Transmission electron microscopy**

Ultra-thin sectioning, negative and positive staining for transmission electron microscopy (TEM) were carried out to visualize the fine structure of rat heart tissue and purified LDs respectively, as previously described[^1^](#_ENREF_1). Simply glutaraldehyde-prefixed heart tissue was post-fixed in 2% osmium tetraoxide for 1 hour at 4°C. Then samples were dehydrated in an increasing solvency of ethanol at room temperature and embedded in Quetol 812. After section with Leica EM UC6 Ultramicrotome (Leica Company), all samples were stained with 2% uranyl acetate for 15 minutes and then with lead citrate for 5 minutes at room temperature. For negative staining, the isolated LDs were placed on a Formvar-carbon coated copper grid and stained with 2% (w/v) uranyl acetate for 30 seconds. For positive staining, a drop of the LDs was placed on a Formvar-carbon-coated nickel grid and fixed with 2% glutaraldehyde in 0.1 mol/L sodium phosphate buffer for 10 minutes, 1% OsO_4_ in 0.1 mol/L sodium phosphate buffer for another 15 minutes, after that, the grid was stained with 0.1% tannic acid for 10 minutes and 4% uranyl acetate for 10 minutes. All the stained grids were viewed with a FEI Tecnai 20 (FEI Company) electron microscope.

**Protein preparation and immunoblotting**

LD proteins were precipitated by 100% acetone, dissolved in 5 × SDS sample buffer and denatured at 95°C for 5 minutes. Cytosol and PNS protein were lysed directly with 5 × SDS loading buffer and denatured at 95°C for 5 minutes. Total membrane was resuspended in buffer B as above-mentioned and precipitated in 7.2% trichloroacetic acid, and subsequently washed with acetone before dissolving in 5 × SDS loading buffer by sonication. After SDS-PAGE, proteins were stained with a silver stain kit (Bio-Rad) or Colloidal Blue stain kit (Invitrogen) or transferred to a PVDF membrane for immunoblotting. Immunoblotting was performed with the primary antibodies against Caveolin-3, Annexin A2, BIP, Rab5, LAMP1 and EEA1 (Millipore), OXPAT (Novus), TIP47 (Abnova Corporation), COX IV (Cell Signaling Technology), Dysferlin (Leica Microsystems), TIM23 (provided by Dr. Quan Chen), ADRP (provided from Dr. Ginette Serrero) and p62 (provided by Dr. Dorothy Mundy) and detected by ECL system. Colloidal Blue Staining was used as loading control.

**Immunofluorescence staining**

Primary cardiomyocytes (seeded on coverslips for 24 hours) or heart cryosections were fixed, blocked and incubated with the indicated primary antibodies against Caveolin-3 (Abcam, ab30750, rabbit polyclonal antibody), Annexin A2 (Abcam, ab41803, rabbit polyclonal antibody), Annexin A11 (Proteintech Group, 10479-2-AP, rabbit polyclonal antibody), ADRP (provided from Dr. Ginette Serrero, mouse monoclonal antibody), OXPAT (Novus, NB110-60509, rabbit polyclonal antibody), ATGL (Cell Signaling Technology, 2138s, rabbit polyclonal antibody), ACSL1 (Cell Signaling Technology, 4047s, rabbit polyclonal antibody), CGI-58 (Abcam, ab80365, rabbit polyclonal antibody), dysferlin (Leica Microsystems, NCL-Halmlet, mouse monoclonal antibody) and VCP (Cell Signaling Technology, 2648s, rabbit polyclonal antibody) at 4°C overnight. Fluorescein-conjugated secondary antibodies (1:100 dilutions) were then added to the samples, followed by 1 hour incubation. Further, cells or cryosections were stained with DAPI and LipidTOX Deep Red (Invitrogen) in the dark for 5 minutes. The slides were washed and covered with mounting medium. Confocal microscopic images were captured with an Olympus FV1000 fluorescence microscope (Olympus).

**TAG measurement**

100 mg heart tissue was washed twice with l mL PBS and dissolved in 200-400 μL 1% Triton X-100 by sonicating 6 times, 6 seconds each time at 200 Watt. The whole tissue lysates were then centrifuged at 10,000 × *g* for 5 minutes at 4ºC. The supernatant was collected into a new Eppendorf tube. TAG in the supernatant was measured using the triacylglycerol assay kit E1003 (Applygen Technologies, China). Protein was quantified using Pierce BCA Protein Assay Kit (Thermo, USA).

**Protein digestion and iTRAQ labeling, LC-MS/MS analysis, protein identification and quantification analysis**

Two groups of normal and dysfunctional heart LDs were isolated for proteomics comparison analysis. The samples for iTRAQ quantitative analysis was prepared according to iTRAQ™ Reagents Protocol (Applied Biosystems, USA) with little modified. Simply, 10 μg LD protein precipitated by acetone was dissolved in 10 μL 8M urea (pH 7.5) plus 10 μL Dissolution Buffer. Then 2 μL Reducing Reagent was added to each sample tube and incubate at 37°C for 1 hour. After that, 1 μL Cysteine Blocking Reagent was added to each sample tube and incubate at room temperature for 10 minutes. Protein digestion by trypsin (Invitrogen, USA) (0.3 μg trypsin to digest 10 μg protein) was carried out after adding Dissolution Buffer (75 μL) dilution. After incubation at 37 ºC overnight, the trypsin digestion process was terminated at -20°C for 30 minutes. The protein digestions were dried in a centrifugal vacuum concentrator, reconstituted with 20 μL Dissolution Buffer plus 70 μL ethanol, then transferred to one iTRAQ™ Reagent vial and incubated at room temperature for 2 hours. Subsequently, all the iTRAQ™ Reagent-labeled tryptic peptides were combined into one tube, dried in a centrifugal vacuum concentrator, and diluted in 200 μL 0.5% formic acid (FA). 50 μL FA-diluted peptides (about 10 ug) were desalted by using C18 ZipTip cleanup (Millipore, USA) according to the [merchandise](javascript:void(0);) instruction. Simply, ZipTip was equilibrated with 100% acetonitrile and 0.1% formic acid for 3 times respectively. Then the peptide sample was loaded on the ZipTip by pipetting the protein digest up and down for 10 times. After that, ZipTip was washed with 0.1% formic acid for 5 times, and the peptides were eluted with 40% /0.1% formic acid and 60% acetonitrile/0.1% formic acid for 3 times separately. Then the sample was dried by a centrifugal vacuum concentrator and diluted in 15μL of 0.5% FA for the following LC-MS/MS analysis.

All nano LC-MS/MS experiments were performed on a Q Exactive (Thermo Scientific, USA) equipped with an Easy n-LC 1,000 HPLC system (Thermo Scientific, USA). The labeled peptides were loaded onto a 100 μm id×2 cm fused silica trap column packed in-house with reversed phase silica (Reprosil-Pur C18 AQ, 5 μm, Dr. Maisch GmbH) and then separated on an a 75 μm id×20 cm C18 column packed with reversed phase silica (Reprosil-Pur C18 AQ, 3 μm, Dr. Maisch GmbH). The peptides bounded on the column were eluted with a 78-minutes linear gradient. The solvent A consisted of 0.1% FA in water solution and the solvent B consisted of 0.1% FA in acetonitrile solution. The segmented gradient was 5–8% B, 8 minutes; 8–22% B, 50 minutes; 22–32% B, 12 minutes; 32-95% B, 1 minute; 95% B, 7 minutes at a flow rate of 280 nL/min.

The MS analysis was performed with Q Exactive mass spectrometer (Thermo Scientific). With the data-dependent acquisition mode, the MS data were acquired at a high resolution 70,000 (m/z 200) across the mass range of 300–1600 m/z. The target value was 3.00E+06 with a maximum injection time of 60 ms. The top 20 precursor ions were selected from each MS full scan with isolation width of 2 m/z for fragmentation in the HCD collision cell with normalized collision energy of 27%. Subsequently, MS/MS spectra were acquired at resolution 17,500 at m/z 200. The target value was 5.00E+04 with a maximum injection time of 80 ms. The dynamic exclusion time was 40 seconds. For nano electrospray ion source setting, the spray voltage was 2.0 kV; no sheath gas flow; the heated capillary temperature was 320 °C. For each analysis, 2 ug peptides were injected and each sample was measured in duplicate.

The raw data from Q Exactive were analyzed with Proteome Discovery version 1.4 using Sequest HT search engine for protein identification and Percolator for FDR (false discovery rate) analysis. The Uniprot rat database (updated on 05 - 2015) was individual used for searching the data from rat sample. Some important searching parameters were set as following: trypsin was selected as enzyme and one missed cleavages were allowed for searching; the mass tolerance of precursor was set as 10 ppm and the product ions tolerance was 0.02 Da.; MMTS was set as a fixed modification of cysteine and methionine oxidation and iTRAQ 4 plex labeled lysine and N-terminus of peptides were specified as variable modifications. FDR analysis was performed with Percolator and FDR <1% was set for protein identification. The peptides confidence was set as high for peptides filter.

Proteins quantification was also performed on Proteome Discovery version 1.4 using the ratio of the intensity of reporter ions from the MS/MS spectra. Only unique peptides of proteins or protein groups were selected for protein relative quantification. The normal heart LDs from two groups labeled with tag 113 and 115 were respectively considered as control reference for calculating the ratios of 114:113 and 116:115, in which the dysfunctional heart LDs from two groups were labeled with tags 115 and 116 respectively.

All ratios were transformed to base 2 logarithm values. Then, 95% confidence intervals (z score = 1.96) were used to determine the cutoff values for significant changes. The normalization to the protein median of each sample was used to corrected experimental bias and the number of minimum protein count must be greater than twenty. The fold change threshold for up or down regulation was set as mean±1.960σ.

**Data mining and bioinformatics**

The characteristics of the identified proteins were analyzed using free online tools: protein molecular weights and isoelectric focusing (IEF) points were calculated using ExPASy; the calculation of protein GRAVY values was carried out by Protein GRAVY. The function and sub-cellular location of all the identified proteins were annotated according to Gene Ontology Annotation database descriptions and categorized manually. Protein associations and classification were revealed by the website program STRING, PANTHER and Uniprot KB.

**Statistical analysis**

Results represent means ± SEM (n=4-5), unless specified. Student’s t-test (two-tailed) was used when two groups of means were compared. Protein expression was quantified by Quantity One software (Bio-Rad) and normalized to gel staining. TAG concentration was normalized to corresponding protein level. Statistical significance was accepted at *p*≤0.05 (*).

**SUPPLEMENTARY FIGURES**


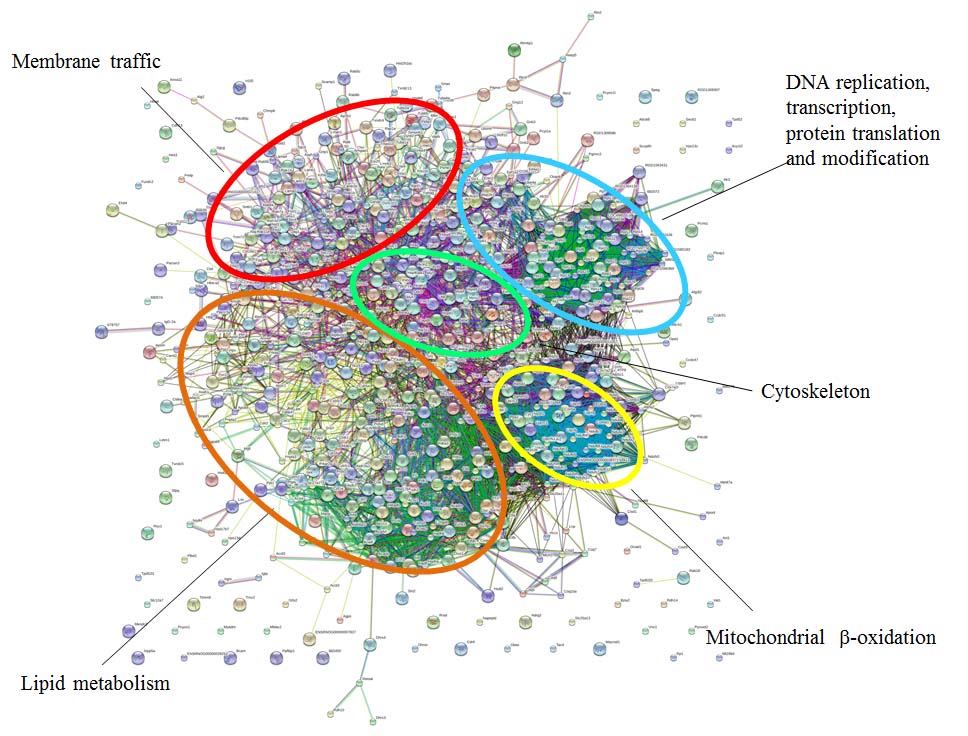
**Supplementary Figure S1.**

**Figure S1.** The depicted interaction network of heart LD-associated proteins. STRING software analysis showed some proteins with close relationship formed unique protein clusters. Orange circle contained most of the lipid metabolic-related proteins. Yellow color embraced the identified proteins associated with mitochondria β-oxidation. Blue color comprised most of identified proteins implicated in DNA replication, transcription, protein translation and modification. Red color contained most identified membrane traffic-related proteins. Green color comprised most identified cytoskeleton proteins.

**Supplementary Figure S2.**

**
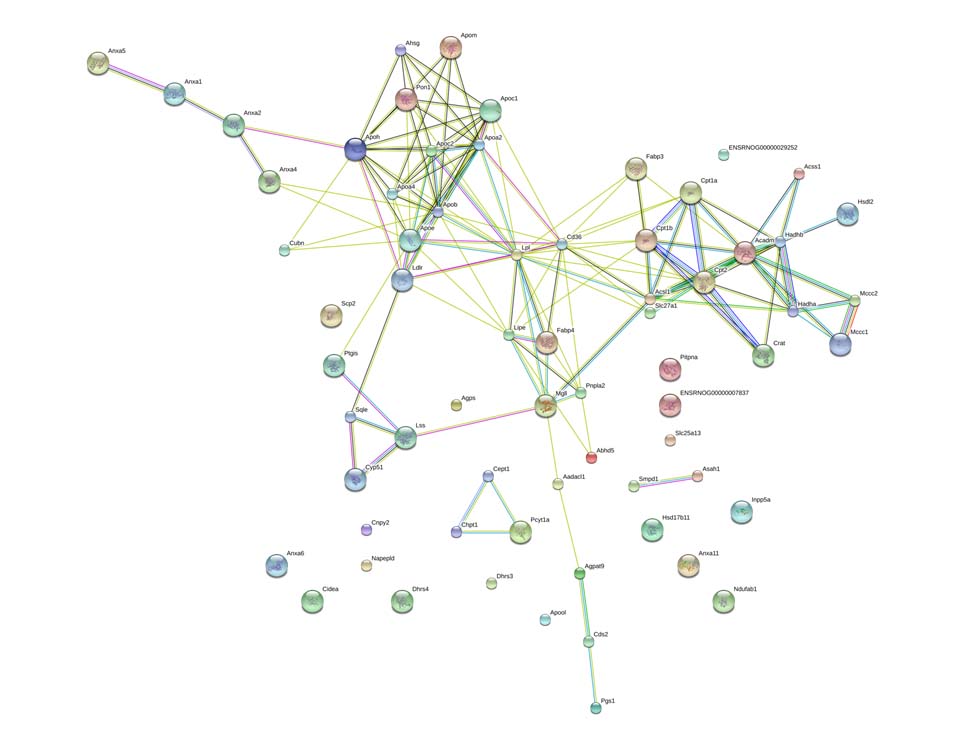
**

**Figure S2.** The association network of identified lipid metabolic proteins and PLIN family proteins was predicted by the website program STRING against rat database.

**Supplementary Figure S3.**


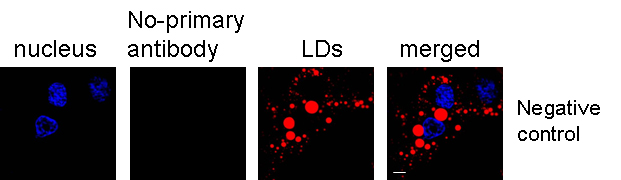


**Figure S3.** Negative control of immunofluoresence image of the primary cardiomyocytes.

The primary cardiomyocytes isolated from neonatal rat were cultured in DMEM supplemented with 10% FBS. 100 µmol/L oleate was added in the medium for 24 hours. The immunostaining was carried out as negative control (without primary antibody incubation) for other immunostaining with indicated primary antibody treatment, Blue: nucleus stained with Hochest33258; Red: LD stained with LipidTOX Deep Red. Bar = 5 µm.

**Supplementary Figure S4.**

**
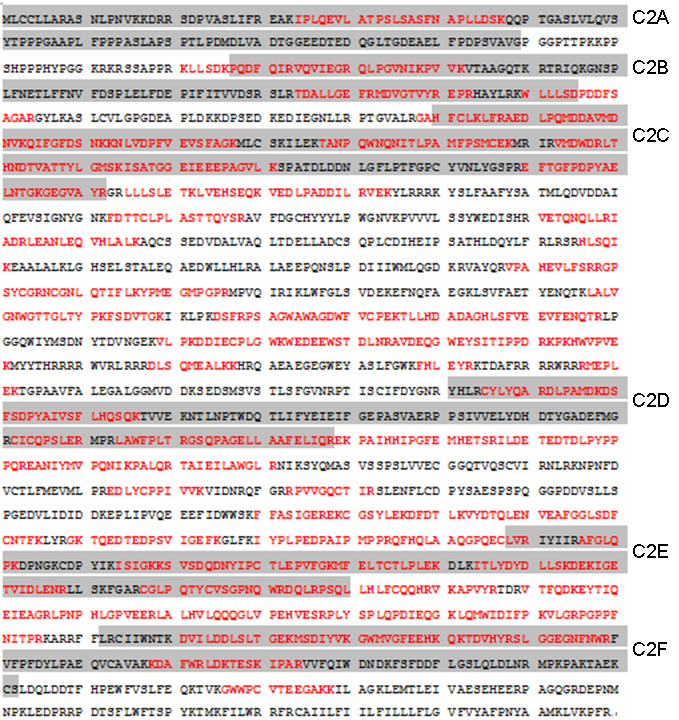
**

**Figure S4.** The identified peptides by mass spectrometry (red words, accounting for 46.72% of total amino acids of dysferlin) and 6 C2 domains (C2A-F, gray

color) of dysferlin.

**Supplementary Figure S5.**

**
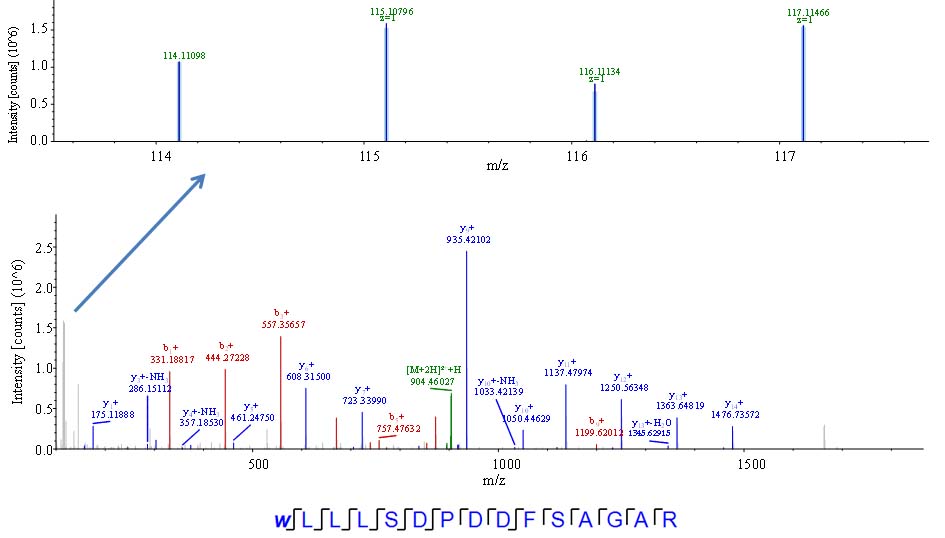
**

**Figure S5.** The MS/MS spectrum of the dysferlin representative peptide WLLLSDPDDFSAGAR was shown (lower panel). The y and b ions were indicated. Arrows pointed to the enlarged view of reporter ion of the peptide.

**Supplementary Figure S6.**


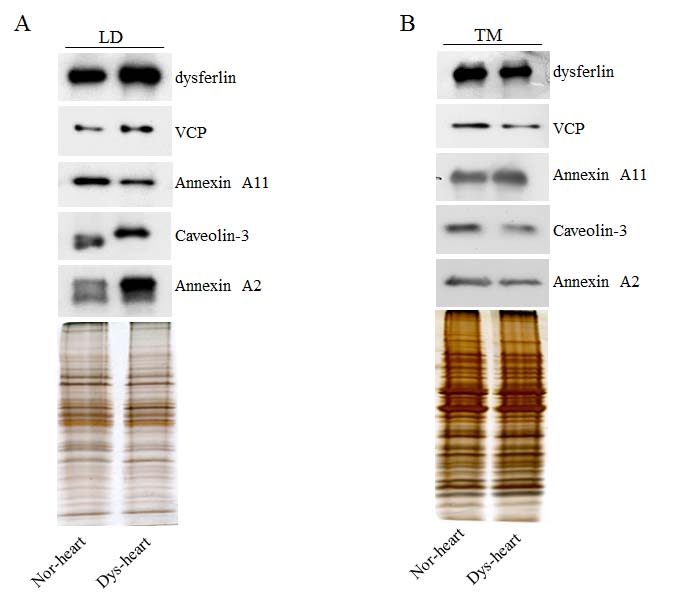
**.**

**Figure S6.** The variation of subcellular distribution of membrane repairing-associated proteins in normal and dysfunctional hearts. **A.** Immunoblotting

was used to detect the LD binding of indicated membrane repairing-associated proteins (dysferlin, VCP, Annexin A11, Caveolin-3, Annexin A2) in

normal and failing rat hearts. **B.** Immunoblotting was used to detect the membrane binding of indicated membrane repairing-associated proteins

in normal and failing rat hearts.

**Supplementary Table S1. Rat heart LD proteins identified and quantified by quantitative mass spectrometry†**

| **UniProt** | **Description** | **Score** | **Seq**  **Cov.** | **Uni. Pep.** | **Ratio1** | **Ratio 2** | **Ratio 3** | **Ratio 4** | **mean** | **SD** | **MW (kDa)** | **pI** | **Gene Symbol** | **Gene ID** | **NCBI Entry** | **GI NO.** | **GRAVY** | **PAF** | **Reported** |  | | | | | | | | | | | | | | | | | |
| --- | --- | --- | --- | --- | --- | --- | --- | --- | --- | --- | --- | --- | --- | --- | --- | --- | --- | --- | --- | --- | --- | --- | --- | --- | --- | --- | --- | --- | --- | --- | --- | --- | --- | --- | --- | --- | --- |
| **lipid metabolism** | | | | | | | | | | | | | | | | | | | |  | | | | | | | | | | | | | | | | | |
| P04638 | Apolipoprotein A-II | 9.92 | 19.61 | 2 | 0.32 | 0.4 | 0.28 | 0.44 | 0.36 | 0.07 | 11.43 | 6.65 | Apoa2 | 25649 | P04638 | 114004 | -0.242 | 0.35 | 1 |  | | | | | | | | | | | | | | | | | |
| P04639 | Apolipoprotein A-I | 161.6 | 70.27 | 22 | 0.53 | 0.49 | 0.49 | 0.54 | 0.51 | 0.02 | 30.04 | 5.74 | Apoa1 | 25081 | P04639 | 146345369 | -0.719 | 1.764 |  |  | | | | | | | | | | | | | | | | | |
| P02651 | Apolipoprotein A-IV | 76.12 | 47.06 | 15 | 0.58 | 0.57 | 0.55 | 0.57 | 0.57 | 0.01 | 44.43 | 5.22 | Apoa4 | 25080 | P02651 | 114008 | -0.667 | 0.518 | 14 |  | | | | | | | | | | | | | | | | | |
| P55159 | Serum paraoxonase/arylesterase 1 | 9.04 | 7.32 | 3 | 0.53 | 0.63 |  |  | 0.58 |  | 39.33 | 5.22 | Pon1 | 84024 | P55159 | 308153573 | 0.051 | 0.076 |  |  | | | | | | | | | | | | | | | | | |
| G3V8D4 | Apolipoprotein C-II (Predicted) | 11.99 | 25.77 | 3 | 0.6 | 0.53 | 0.61 | 0.59 | 0.58 | 0.04 | 10.69 | 4.61 | Apoc2 | 292697 | NP_001078821 | 145553986 | -0.031 | 0.468 | 1 |  | | | | | | | | | | | | | | | | | |
| Q6QA69 | 1-acylglycerol-3-phosphate O-acyltransferase ABHD5 | 150.8 | 65.24 | 14 | 0.62 | 0.59 | 0.48 | 0.62 | 0.58 | 0.07 | 39.08 | 6.61 | Abhd5 | 316122 | Q6QA69 | 73921644 | -0.214 | 1.024 | 1 |  | | | | | | | | | | | | | | | | | |
| P0C548 | Patatin-like phospholipase domain-containing protein 2 | 223.8 | 53.77 | 24 | 0.55 | 0.55 | 0.63 | 0.59 | 0.58 | 0.04 | 52.53 | 6.61 | Pnpla2 | 100911615,361676 | P0C548 | 150403923 | -0.077 | 1.332 | 2,1 |  | | | | | | | | | | | | | | | | | |
| Q06000 | Lipoprotein lipase | 40.46 | 28.48 | 11 | 0.72 | 0.71 | 0.66 | 0.8 | 0.72 | 0.06 | 53.05 | 8.13 | Lpl | 24539 | Q06000 | 462538 | -0.336 | 0.245 | 12 |  | | | | | | | | | | | | | | | | | |
| F1LX28 | Protein Acot11 (Fragment) | 72.68 | 32.08 | 17 | 0.73 | 0.72 | 0.78 | 0.72 | 0.74 | 0.03 | 65.77 | 6.57 | Acot11 | 100363074 | NP_001258312 | 406035346 | -0.35 | 0.471 |  |  | | | | | | | | | | | | | | | | | |
| F1LP67 | Prostacyclin synthase (Fragment) | 7.17 | 6.89 | 2 | 0.68 | 0.79 |  |  | 0.74 |  | 51.75 | 6.71 | Ptgis | 25527 | NP_113745 | 13928752 | -0.317 | 0.039 |  |  | | | | | | | | | | | | | | | | | |
| Q5XI77 | Annexin | 77.91 | 35.39 | 17 | 0.77 | 0.76 | 0.74 | 0.71 | 0.76 | 0.03 | 54.13 | 7.65 | Anxa11 | 290527 | Q5XI77 | 81910359 | -0.511 | 0.462 | 7,10 |  | | | | | | | | | | | | | | | | | |
| P97849 | Long-chain fatty acid transport protein 1 | 38.15 | 15.79 | 9 | 0.84 | 0.83 | 0.79 | 0.8 | 0.81 | 0.02 | 71.24 | 8.46 | Slc27a1 | 94172 | P97849 | 2492888 | -0.015 | 0.182 |  |  | | | | | | | | | | | | | | | | | |
| P18886 | Carnitine O-palmitoyltransferase 2, mitochondrial | 281.7 | 60.33 | 45 | 0.83 | 0.8 | 0.82 | 0.81 | 0.82 | 0.01 | 74.06 | 7.33 | Cpt2 | 25413 | P18886 | 117289 | -0.295 | 1.391 |  |  | | | | | | | | | | | | | | | | | |
| P19836 | Choline-phosphate cytidylyltransferase A | 101.1 | 58.31 | 21 | 0.83 | 0.82 | 0.82 | 0.83 | 0.82 | 0.01 | 41.65 | 7.03 | Pcyt1a | 140544 | P19836 | 1345858 | -0.708 | 1.008 |  |  | | | | | | | | | | | | | | | | | |
| D3ZF13 | Acyl carrier protein | 29.05 | 27.56 | 4 | 0.74 | 0.91 | 0.8 | 0.86 | 0.83 | 0.07 | 17.5 | 5.07 | LOC683884 | 293453 | NP_001099764 | 157820787 | 0.038 | 0.514 |  |  | | | | | | | | | | | | | | | | | |
| P15304-2 | Isoform 2 of Hormone-sensitive lipase | 137.8 | 38.67 | 23 | 0.77 | 0.83 | 0.82 | 0.89 | 0.83 | 0.05 | 84.12 | 6.95 | Lipe | 25330 | NP_036991 | 6981164 | -0.307 | 0.487 | 12,1 |  | | | | | | | | | | | | | | | | | |
| P19939 | Apolipoprotein C-I | 15.83 | 28.41 | 3 | 0.79 | 0.89 | 0.83 | 0.85 | 0.84 | 0.04 | 9.85 | 9.09 | Apoc1 | 25292 | P19939 | 114018 | -0.057 | 0.812 | 1 |  | | | | | | | | | | | | | | | | | |
| Q8R431 | Monoglyceride lipase | 79.55 | 53.47 | 11 | 0.86 | 0.92 | 0.85 | 0.84 | 0.87 | 0.04 | 33.48 | 7.37 | Mgll | 29254 | Q8R431 | 47116974 | -0.013 | 0.777 | 8,1 |  | | | | | | | | | | | | | | | | | |
| P16446 | Phosphatidylinositol transfer protein alpha isoform | 9.38 | 12.55 | 2 | 0.85 | 0.88 |  |  | 0.87 |  | 31.89 | 6.37 | Pitpna | 29525 | P16446 | 130771 | -0.739 | 0.094 | 1 |  | | | | | | | | | | | | | | | | | |
| P16303 | Carboxylesterase 1D | 22.11 | 13.1 | 7 | 0.83 | 0.92 | 0.86 | 0.89 | 0.88 | 0.04 | 62.11 | 6.54 | Ces1d | 113902 | P16303 | 57013350 | -0.105 | 0.145 | 5,14 |  | | | | | | | | | | | | | | | | | |
| Q8VID1 | Dehydrogenase/reductase SDR family member 4 | 20.64 | 11.83 | 4 | 0.81 | 1.05 | 0.76 | 0.91 | 0.88 | 0.13 | 29.8 | 9.55 | Dhrs4 | 266686 | Q8VID1 | 308153437 | 0.156 | 0.235 |  |  | | | | | | | | | | | | | | | | | |
| Q704S8 | Carnitine O-acetyltransferase | 18.97 | 6.71 | 4 | 0.87 | 0.8 | 1.16 | 0.72 | 0.89 | 0.19 | 70.76 | 8.54 | Crat | 311849 | Q704S8 | 59797483 | -0.25 | 0.099 |  |  | | | | | | | | | | | | | | | | | |
| P02650 | Apolipoprotein E | 153.5 | 65.71 | 30 | 0.95 | 0.87 | 0.87 | 0.89 | 0.89 | 0.04 | 35.73 | 5.27 | Apoe | 25728 | P02650 | 1703338 | -0.707 | 1.959 | 14,1 |  | | | | | | | | | | | | | | | | | |
| P14630 | Apolipoprotein M | 6 | 16.84 | 3 | 0.86 | 0.85 | 0.95 | 0.94 | 0.86 | 0.05 | 21.5 | 6.09 | Apom | 55939 | P14630 | 19856159 | -0.333 | 0.186 |  |  | | | | | | | | | | | | | | | | | |
| Q07969 | Platelet glycoprotein 4 | 74.75 | 25 | 11 | 0.87 | 0.95 |  |  | 0.91 |  | 52.7 | 8.56 | Cd36 | 29184 | Q07969 | 146345388 | -0.048 | 0.455 |  |  | | | | | | | | | | | | | | | | | |
| Q63704 | Carnitine O-palmitoyltransferase 1, muscle isoform | 131.4 | 33.55 | 24 | 0.89 | 0.95 | 0.9 | 0.92 | 0.91 | 0.03 | 88.16 | 8.6 | Cpt1b | 25756 | Q63704 | 2493498 | -0.242 | 0.635 |  |  | | | | | | | | | | | | | | | | | |
| Q4V8F9 | Hydroxysteroid dehydrogenase-like protein 2 | 62.5 | 29.2 | 12 | 0.95 | 0.88 | 0.95 | 0.87 | 0.91 | 0.04 | 58.31 | 6.19 | Hsdl2 | 313200 | Q4V8F9 | 81907928 | -0.502 | 0.36 | 4,7,8,12,15 |  | | | | | | | | | | | | | | | | | |
| Q5U362 | Annexin | 14.48 | 12.54 | 4 |  |  | 0.93 | 0.96 | 0.95 |  | 35.87 | 5.57 | Anxa4 | 79124 | Q5U362 | 81910262 | -0.431 | 0.139 | 7 |  | | | | | | | | | | | | | | | | | |
| Q07936 | Annexin A2 | 65.55 | 48.08 | 14 | 0.99 | 0.9 | 0.99 | 0.97 | 0.96 | 0.04 | 38.65 | 7.69 | Anxa2 | 56611 | Q07936 | 584760 | -0.526 | 0.647 | 1 |  | | | | | | | | | | | | | | | | | |
| P06759 | Apolipoprotein C-III | 12.71 | 31.68 | 2 | 1.11 | 0.82 | 0 | 0 | 0.96 | 0.2 | 11.11 | 4.77 | Apoc3 | 24207 | P06759 | 6226553 | -0.198 | 0.63 | 1 |  | | | | | | | | | | | | | | | | | |
| Q5U1W6 | Apolipoprotein O-like | 40.97 | 27.56 | 5 | 0.96 | 0.96 | 0.95 | 0.98 | 0.96 | 0.01 | 28.22 | 9.01 | Apool | 317191 | Q5U1W6 | 81883434 | -0.407 | 0.532 | 1 |  | | | | | | | | | | | | | | | | | |
| P55260 | Annexin A4 | 12.82 | 19.44 | 5 | 0.99 | 0.94 | 0 | 0 | 0.97 | 0.04 | 35.83 | 5.44 | Anxa4 | 79124 | P55260 | 37999910 | -0.423 | 0.14 |  |  | | | | | | | | | | | | | | | | | |
| Q6AYS8 | Estradiol 17-beta-dehydrogenase 11 | 56.2 | 45.64 | 11 | 0.93 | 1.02 | 0.96 | 0.97 | 0.97 | 0.04 | 32.92 | 8.63 | Hsd17b11 | 289456 | Q6AYS8 | 73620788 | 0.261 | 0.577 |  |  | | | | | | | | | | | | | | | | | |
| P48037 | Annexin A6 | 54.87 | 25.11 | 12 | 0.95 | 1 |  |  | 0.98 |  | 75.71 | 5.57 | Anxa6 | 79125 | P48037 | 1351943 | -0.432 | 0.198 | 1 |  | | | | | | | | | | | | | | | | | |
| F1LQ28 | Platelet glycoprotein 4 | 57.77 | 19.07 | 10 |  |  | 0.96 | 1.02 | 0.99 |  | 52.63 | 8.65 | Cd36 | 29184 | NP_113749 | 48675379 | -0.041 | 0.399 |  |  | | | | | | | | | | | | | | | | | |
| B2GV54 | Neutral cholesterol ester hydrolase 1 | 19.81 | 11.52 | 3 | 1.06 | 0.94 | 0.97 | 0.99 | 1 | 0.05 | 45.79 | 7.05 | Nceh1 | 294930 | B2GV54 | 212288176 | 0.007 | 0.218 |  |  | | | | | | | | | | | | | | | | | |
| Q5XIA6 | Protein Smpd1 | 8.19 | 5.26 | 3 | 1.04 | 1.02 | 1.04 | 0.95 | 1.01 | 0.04 | 69.72 | 7.4 | Smpd1 | 308909 | Q5XIA6 | 81883732 | -0.189 | 0.043 |  |  | | | | | | | | | | | | | | | | | |
| P07150 | Annexin A1 | 6.13 | 7.8 | 2 |  |  | 1.03 | 1 | 1.01 |  | 38.8 | 7.34 | Anxa1 | 25380 | P07150 | 113947 | -0.431 | 0.052 | 1 |  | | | | | | | | | | | | | | | | | |
| P07483 | Fatty acid-binding protein, heart | 85.11 | 64.66 | 12 | 1.03 | 1.01 | 1.05 | 1.01 | 1.02 | 0.02 | 14.77 | 6.33 | Fabp3 | 79131 | P07483 | 119804 | -0.373 | 1.896 | 5,8 |  | | | | | | | | | | | | | | | | | |
| P18163 | Long-chain-fatty-acid--CoA ligase 1 | 639.1 | 65.52 | 58 | 1.03 | 1.03 | 1.06 | 1.01 | 1.03 | 0.02 | 78.13 | 6.99 | Acsl1 | 25288 | P18163 | 126011 | -0.08 | 2.509 |  |  | | | | | | | | | | | | | | | | | |
| Q6P7S1 | Acid ceramidase | 167.6 | 55.58 | 25 | 1.05 | 1.02 | 1.07 | 1 | 1.04 | 0.03 | 44.41 | 8.31 | Asah1 | 84431 | Q6P7S1 | 81885370 | -0.175 | 1.373 | 1 |  | | | | | | | | | | | | | | | | | |
| Q9EQR2 | Alkyldihydroxyacetonephosphate synthase, peroxisomal | 18.12 | 13.04 | 6 | 1.05 | 0.99 | 1.02 | 1.08 | 1.04 | 0.04 | 71.54 | 7.36 | Agps | 84114 | Q9EQR2 | 81872483 | -0.346 | 0.098 |  |  | | | | | | | | | | | | | | | | | |
| A0JN30 | Canopy 2 homolog (Zebrafish) | 7.15 | 14.29 | 2 | 1.15 | 1.22 | 1.04 | 0.79 | 1.19 | 0.19 | 20.7 | 5.14 | Cnpy2 | 685814 | NP_001071053 | 117606182 | -0.479 | 0.145 |  |  | | | | | | | | | | | | | | | | | |
| P70623 | Fatty acid-binding protein, adipocyte | 22.19 | 31.82 | 6 | 1.08 | 1.07 | 1.06 | 1 | 1.05 | 0.04 | 14.7 | 7.94 | Fabp4 | 79451 | P70623 | 2494405 | -0.173 | 0.476 | 8 |  | | | | | | | | | | | | | | | | | |
| Q5XIT9 | Methylcrotonoyl-CoA carboxylase beta chain, mitochondrial | 37.3 | 23.27 | 10 | 1.04 | 1.16 | 1.04 | 1.04 | 1.07 | 0.06 | 61.48 | 8.31 | Mccc2 | 361884 | Q5XIT9 | 81883845 | -0.178 | 0.211 |  |  | | | | | | | | | | | | | | | | | |
| D3ZZN3 | Protein Acss1 | 14.07 | 11.14 | 5 | 1.12 | 1.06 | 1.09 | 1.03 | 1.08 | 0.04 | 74.84 | 6.86 | Acss1 | 296259 | NP_001099994 | 157818027 | -0.109 | 0.067 |  |  | | | | | | | | | | | | | | | | | |
| P11915-2 | Isoform SCP2 of Non-specific lipid-transfer protein | 19.81 | 46.15 | 6 | 1.11 | 1.19 | 1.12 | 0.91 | 1.08 | 0.12 | 15.29 | 8.57 | Scp2 | 6342 | P11915 | 128391 | -0.222 | 0.523 |  |  | | | | | | | | | | | | | | | | | |
| Q4V8J4 | Glycerol-3-phosphate acyltransferase 3 | 8.79 | 10.07 | 4 | 1.05 | 1.21 | 0.97 | 1.15 | 1.09 | 0.1 | 50.98 | 8.56 | Agpat9 | 305166 | Q4V8J4 | 81907944 | 0.058 | 0.078 | 9 |  | | | | | | | | | | | | | | | | | |
| G3V796 | Acetyl-Coenzyme A dehydrogenase, medium chain | 53.6 | 29.45 | 10 | 1.09 | 1.11 | 1.08 | 1.11 | 1.09 | 0.02 | 46.54 | 8.41 | Acadm | 24158 | NP_058682 | 292494885 | -0.284 | 0.365 | 1 |  | | | | | | | | | | | | | | | | | |
| D4A5W8 | Protein Pgs1 | 12.22 | 8.5 | 4 | 1.12 | 1.03 | 1.06 | 1.26 | 1.07 | 0.11 | 62.43 | 8.79 | Pgs1 | 303698 | XP_001081747 | 293340596 | -0.123 | 0.064 |  |  | | | | | | | | | | | | | | | | | |
| F1LX07 | Protein Slc25a12 (Fragment) | 76.46 | 26.22 | 11 | 1.16 | 1.06 | 1.12 | 1.22 | 1.14 | 0.07 | 74.18 | 8.75 | LOC100360985 | 362145 | XP_008773742 | 672017397 | -0.026 | 0.31 |  |  | | | | | | | | | | | | | | | | | |
| D3ZZX1 | Inositol polyphosphate-5-phosphatase A (Predicted), isoform CRA_a | 5.15 | 5.34 | 2 | 1.12 | 1.18 |  |  | 1.15 |  | 47.59 | 6.92 | Inpp5a | 365382 | NP_001102393 | 157821515 | -0.414 | 0.042 |  |  | | | | | | | | | | | | | | | | | |
| Q5I0M1 | Apolipoprotein H | 28.73 | 16.52 | 4 | 1.16 | 1.17 | 1.07 | 1.21 | 1.15 | 0.06 | 38.43 | 8.21 | Apoh | 287774 | Q5I0M1 | 81883011 | -0.254 | 0.286 |  |  | | | | | | | | | | | | | | | | | |
| F1LZW6 | Protein Slc25a13 (Fragment) | 67.89 | 32.3 | 12 | 1.19 | 1.13 | 1.12 | 1.2 | 1.16 | 0.04 | 74.34 | 8.7 | Slc25a13 | 362322 | XP_001054092 | 392339831 | 0.022 | 0.309 |  |  | | | | | | | | | | | | | | | | | |
| Q769K2 | N-acyl-phosphatidylethanolamine-hydrolyzing phospholipase D | 102.7 | 44.7 | 16 | 1.12 | 1.24 | 1.18 | 1.13 | 1.17 | 0.06 | 45.69 | 6.04 | Napepld | 296757 | Q769K2 | 81864936 | -0.571 | 0.832 |  |  | | | | | | | | | | | | | | | | | |
| Q3B7V0 | Dehydrogenase/reductase (SDR family) member 3 | 94.63 | 39.74 | 12 | 1.34 | 1.23 | 1.31 | 1.19 | 1.27 | 0.07 | 33.59 | 8.69 | Dhrs3 | 313689 | Q3B7V0 | 123786315 | 0.128 | 0.893 | 4,7,8,1 |  | | | | | | | | | | | | | | | | | |
| P14668 | Annexin A5 | 28.61 | 31.35 | 11 | 1.33 | 1.3 | 1.47 | 1.32 | 1.31 | 0.08 | 35.72 | 5.05 | Anxa5 | 25673 | P14668 | 4033508 | -0.322 | 0.336 | 7 |  | | | | | | | | | | | | | | | | | |
| P48450 | Lanosterol synthase | 50.06 | 20.74 | 13 | 1.43 | 1.53 | 1.3 | 1.38 | 1.41 | 0.09 | 83.25 | 6.67 | Lss | 81681 | P48450 | 62296496 | -0.317 | 0.24 | 4,6,8,9,12,13,15,1 |  | | | | | | | | | | | | | | | | | |
| D4ACH9 | Protein Cidea | 99.71 | 39.49 | 14 | 1.52 | 1.33 | 1.45 | 1.34 | 1.41 | 0.09 | 22.11 | 9.55 | Cidea | 291541 | NP_001163938 | 281427180 | -0.018 | 1.764 |  |  | | | | | | | | | | | | | | | | | |
| Q5HZX7 | UPF0554 protein C2orf43 homolog | 33.71 | 31.38 | 7 | 1.8 | 1.06 | 1.34 | 1.48 | 1.42 | 0.31 | 36.86 | 7.93 |  | 313949 | Q5HZX7 | 81909703 | 0.022 | 0.353 |  |  | | | | | | | | | | | | | | | | | |
| Q5PPL3 | Sterol-4-alpha-carboxylate 3-dehydrogenase, decarboxylating | 68.49 | 43.09 | 12 | 1.5 | 1.35 | 1.63 | 1.27 | 1.44 | 0.16 | 40.39 | 8.85 | Nsdhl | 309262 | Q5PPL3 | 81883212 | -0.129 | 0.57 |  |  | | | | | | | | | | | | | | | | | |
| O35547 | Long-chain-fatty-acid--CoA ligase 4 | 41.08 | 14.03 | 5 | 1.47 | 1.31 | 1.65 | 1.33 | 1.44 | 0.16 | 74.28 | 8.02 | Acsl4 | 113976 | O35547 | 6016483 | -0.256 | 0.215 |  |  | | | | | | | | | | | | | | | | | |
| D3Z9J9 | Protein Pnpla3 | 19.98 | 18.12 | 6 | 1.59 | 1.52 | 1.37 | 1.31 | 1.45 | 0.13 | 45.88 | 7.17 | Pnpla3 | 362972 | NP_001269253 | 537361028 | 0.08 | 0.196 |  |  | | | | | | | | | | | | | | | | | |
| Q62904 | 3-keto-steroid reductase | 20.59 | 21.56 | 5 | 1.45 | 1.37 | 1.61 | 1.37 | 1.41 | 0.11 | 37.35 | 7.52 | Hsd17b7 | 29540 | Q62904 | 8134405 | -0.066 | 0.187 |  |  | | | | | | | | | | | | | | | | | |
| Q63151-2 | Isoform Short of Long-chain-fatty-acid--CoA ligase 3 | 112.6 | 34.98 | 19 | 1.49 | 1.53 | 1.56 | 1.41 | 1.5 | 0.07 | 79.25 | 8.35 | Acsl3 | 114024 | NP_476448 | 16923952 | -0.161 | 0.479 |  |  | | | | | | | | | | | | | | | | | |
| O35244 | Peroxiredoxin-6 | 8.36 | 12.05 | 2 | 1.43 | 1.58 | 1.42 | 1.58 | 1.5 | 0.09 | 24.8 | 5.94 | Prdx6 | 94167 | O35244 | 5902791 | -0.215 | 0.081 |  |  | | | | | | | | | | | | | | | | | |
| Q80ZF7 | Retinol dehydrogenase 10 | 80.34 | 42.82 | 10 | 1.58 | 1.41 | 1.6 | 1.48 | 1.52 | 0.09 | 38.04 | 7.37 | Rdh10 | 353252 | Q80ZF7 | 81895476 | 0.14 | 0.578 | 1 |  | | | | | | | | | | | | | | | | | |
| P55797 | Apolipoprotein C-IV | 37.13 | 42.74 | 6 | 1.55 | 1.5 | 1.65 | 1.61 | 1.58 | 0.06 | 14.52 | 9.32 | Apoc4 | 680551 | P55797 | 119370278 | -0.352 | 0.757 |  |  | | | | | | | | | | | | | | | | | |
| **membrane traffic** | | | | | | | | | | | | | | | | | | |  | |  |  |  |  |  |  |  |  |  |  |  |  |  |  |  |  |  |
| P41350 | Caveolin-1 | 48.89 | 33.71 | 6 | 0.4 | 0.6 | 0.38 | 0.5 | 0.47 | 0.1 | 20.54 | 5.53 | Cav1 | 25404 | P41350 | 51338710 | 0.029 | 1.217 | 7,8 |  | | | | | | | | | | | | | | | | | |
| O35303-2 | Isoform 2 of Dynamin-1-like protein | 79.28 | 32.68 | 19 | 0.63 | 0.79 |  |  | 0.71 |  | 79.9 | 6.93 | Dnm1l | 114114 | XP_006248778 | 564377808 | -0.292 | 0.35 | 1 |  | | | | | | | | | | | | | | | | | |
| E9PTW1 | Protein Scamp3 | 8.6 | 9.14 | 2 | 0.76 | 0.81 | 0.65 | 0.73 | 0.74 | 0.06 | 38.47 | 6.76 | Scamp | 65169 | NP_113912.1 | 274325671 | 0.032 | 0.052 |  |  | | | | | | | | | | | | | | | | | |
| O35303-6 | Isoform 6 of Dynamin-1-like protein | 80.54 | 34.54 | 21 |  |  | 0.75 | 0.76 | 0.76 |  | 80.05 | 7.08 | Dnm1l | 114114 | XP_006248778 | 564377808 | -0.292 | 0.362 | 1 |  | | | | | | | | | | | | | | | | | |
| B0BNG3 | Lman2 protein | 5.84 | 5.87 | 2 |  |  | 0.73 | 0.89 | 0.81 |  | 40.37 | 6.95 | Lman2 | 290994 | NP_001108496 | 169234844 | -0.381 | 0.05 |  |  | | | | | | | | | | | | | | | | | |
| Q63584 | Transmembrane emp24 domain-containing protein 10 | 26.53 | 19.18 | 4 | 0.76 | 0.83 | 0.78 | 0.88 | 0.81 | 0.05 | 24.84 | 6.42 | Tmed10 | 84599 | Q63584 | 62906896 | -0.127 | 0.322 |  |  | | | | | | | | | | | | | | | | | |
| Q5I0E7 | Transmembrane emp24 domain-containing protein 9 | 8.11 | 15.32 | 3 | 0.78 | 0.9 | 0.67 | 1.01 | 0.84 | 0.15 | 27.01 | 8.27 | Tmed9 | 361207 | Q5I0E7 | 81889018 | -0.302 | 0.111 |  |  | | | | | | | | | | | | | | | | | |
| D3ZR27 | Mitofusin-1 | 4.96 | 3.1 | 2 | 0.75 | 0.96 |  |  | 0.85 |  | 83.6 | 6.43 | Mfn1 | 192647 | EDM01190 | 149048649 | -0.225 | 0.024 |  |  | | | | | | | | | | | | | | | | | |
| Q62991 | Sec1 family domain-containing protein 1 | 8.04 | 5.49 | 2 |  |  | 0.77 | 0.95 | 0 |  | 72.22 | 6.51 | Scfd1 | 54350 | Q62991 | 51316554 | -0.324 | 0.028 |  |  | | | | | | | | | | | | | | | | | |
| Q6DGF2 | Enthoprotin | 11.61 | 9.53 | 4 | 1 | 0.82 | 1.06 | 0.6 | 0.87 | 0.21 | 52.11 | 6.25 | Clint1 | 360515 | Q6DGF2 | 81884721 | -0.766 | 0.096 |  |  | | | | | | | | | | | | | | | | | |
| Q4KM74 | Vesicle-trafficking protein SEC22b | 6.93 | 10.23 | 2 | 0.93 | 0.83 |  |  | 0.88 |  | 24.72 | 8.51 | Sec22b | 310710 | Q4KM74 | 116256065 | -0.182 | 0.081 |  |  | | | | | | | | | | | | | | | | | |
| P56603 | Secretory carrier-associated membrane protein 1 | 5.69 | 10.06 | 2 |  |  | 0.95 | 0.9 | 0.92 |  | 37.97 | 7.71 | Scamp1 | 29521 | P56603 | 3914958 | -0.073 | 0.053 |  |  | | | | | | | | | | | | | | | | | |
| Q3MIE4 | Synaptic vesicle membrane protein VAT-1 homolog | 48.43 | 30.45 | 11 | 0.9 | 0.91 | 0.95 | 0.94 | 0.93 | 0.02 | 43.09 | 6.62 | Vat1 | 287721 | Q3MIE4 | 123780797 | -0.048 | 0.348 | 8,15 |  | | | | | | | | | | | | | | | | | |
| P62944 | AP-2 complex subunit beta | 4.11 | 2.13 | 2 | 0.92 | 0.97 | 1.02 | 0.82 | 0.93 | 0.09 | 104.5 | 5.38 | Ap2b1 | 140670 | P62944 | 51702208 | -0.095 | 0.029 |  |  | | | | | | | | | | | | | | | | | |
| M0RDQ6 | Protein RGD1561609 | 9.15 | 5.62 | 3 | 1 | 0.88 |  |  | 0.94 |  | 90.7 | 7.97 | RGD1561609 | 306117 | XP_003752885 | 392333406 | -0.592 | 0.033 |  |  | | | | | | | | | | | | | | | | | |
| D4A376 | Ras-related protein Rab-12 | 12.88 | 13.9 | 2 |  |  | 0.94 | 0.94 | 0.94 |  | 20.85 | 9.31 | Rab12 | 25530 | NP_037149 | 158186685 | -0.477 | 0.24 |  |  | | | | | | | | | | | | | | | | | |
| Q9WVB1 | Ras-related protein Rab-6A | 23.52 | 24.04 | 4 | 0.87 | 0.96 | 0.95 | 1 | 0.95 | 0.06 | 23.57 | 5.54 | Rab6a | 84379 | Q9WVB1 | 313104164 | -0.416 | 0.339 | 4 |  | | | | | | | | | | | | | | | | | |
| F1M779 | Clathrin heavy chain 1 | 18.04 | 4.42 | 6 | 0.93 | 0.95 | 0.87 | 1.06 | 0.95 | 0.08 | 191.4 | 5.69 | Cltc | 54241 | XP_006247171 | 564373740 | -0.241 | 0.031 | 7 |  | | | | | | | | | | | | | | | | | |
| Q2TA68-3 | Isoform 3 of Dynamin-like 120 kDa protein, mitochondrial | 139.9 | 34.1 | 30 | 0.96 | 1.01 | 0.91 | 0.96 | 0.96 | 0.04 | 115.5 | 7.53 | Opa1 | 171116 | NP_598269 | 148747459 | -0.587 | 0.398 |  |  | | | | | | | | | | | | | | | | | |
| P21588 | 5'-nucleotidase | 7.93 | 5.56 | 2 |  |  | 1.11 | 0.82 | 0.96 |  | 63.93 | 6.98 | Nt5e | 58813 | P21588 | 112826 | -0.054 | 0.031 |  |  | | | | | | | | | | | | | | | | | |
| Q9Z1E1 | Flotillin-1 | 40.53 | 31.54 | 13 | 0.98 | 0.97 | 0.95 | 0.97 | 0.96 | 0.01 | 47.47 | 7.15 | Flot1 | 64665 | Q9Z1E1 | 13124118 | -0.356 | 0.295 | 7 |  | | | | | | | | | | | | | | | | | |
| G3V6T1 | Protein Copa | 8.29 | 2.53 | 2 |  |  | 0.86 | 1.09 | 0.97 |  | 138.3 | 7.65 | Copa | 304978 | AAH91312.1 | 197384345 | -0.279 | 0.014 |  |  | | | | | | | | | | | | | | | | | |
| Q5U316 | Ras-related protein Rab-35 | 31.7 | 32.34 | 4 | 1.01 | 0.94 |  |  | 0.97 |  | 23.01 | 8.29 | Rab35 | 288700 | Q5U316 | 62900797 | -0.473 | 0.565 | 7,16,1 |  | | | | | | | | | | | | | | | | | |
| G3V8I4 | Syntaxin 4A (Placental), isoform CRA_a | 4.46 | 10.4 | 3 | 0.97 | 1 |  |  | 0.99 |  | 34.17 | 6.14 | Stx4 | 81803 | EDM17224 | 149067672 | -0.558 | 0.088 |  |  | | | | | | | | | | | | | | | | | |
| Q4V8H8 | EH domain-containing protein 2 | 93.06 | 32.6 | 13 | 1.01 | 0.97 | 1.03 | 1 | 1 | 0.03 | 61.2 | 6.55 | Ehd2 | 361512 | Q4V8H8 | 81908709 | -0.318 | 0.539 |  |  | | | | | | | | | | | | | | | | | |
| Q62902 | Protein ERGIC-53 | 16.56 | 8.12 | 3 |  |  | 0.94 | 1.08 | 1.01 |  | 57.92 | 6.34 | Lman1 | 116666 | Q62902 | 29611707 | -0.539 | 0.069 |  |  | | | | | | | | | | | | | | | | | |
| Q9Z2S9-3 | Isoform 4 of Flotillin-2 | 30.69 | 36.94 | 12 | 1.07 | 1.08 | 1.07 | 1.06 | 1.07 | 0.01 | 41.73 | 5.29 | Flot2 | 83764 | NP_001257729 | 399154173 | -0.159 | 0.312 | 7 |  | | | | | | | | | | | | | | | | | |
| B0BNK1 | Protein Rab5c | 15.49 | 22.69 | 3 | 1.23 | 0.97 | 1.04 | 1.05 | 1.07 | 0.11 | 23.41 | 8.41 | Rab5c | 287709 | NP_001099310 | 347800697 | -0.309 | 0.299 | 2,12,13,1 |  | | | | | | | | | | | | | | | | | |
| P70550 | Ras-related protein Rab-8B | 34.67 | 31.88 | 3 |  |  | 1.13 | 1.04 | 1.09 |  | 23.59 | 9.07 | Rab8b | 266688 | P70550 | 2500066 | -0.372 | 0.636 | 1 |  | | | | | | | | | | | | | | | | | |
| B0BMW0 | RAB14, member RAS oncogene family | 91.35 | 60.93 | 13 | 1.16 | 1.07 | 1.11 | 1 | 1.11 | 0.07 | 23.88 | 6.21 | Rab14 | 94197 | XP_006234111 | 564340557 | -0.399 | 1.214 | 7,12,1 |  | | | | | | | | | | | | | | | | | |
| P63045 | Vesicle-associated membrane protein 2 | 3.7 | 21.55 | 2 |  |  | 1.09 | 1.09 | 1.09 |  | 12.68 | 8.13 | Vamp2 | 24803 | P63045 | 51704188 | 0.004 | 0.158 | 1 |  | | | | | | | | | | | | | | | | | |
| P63012 | Ras-related protein Rab-3A | 36.34 | 28.64 | 4 | 0.99 | 1.33 | 0.96 | 1.22 | 1.12 | 0.18 | 24.95 | 5.03 | Rab3a | 25531 | P63012 | 51702793 | -0.49 | 0.561 | 8 |  | | | | | | | | | | | | | | | | | |
| Q6NYB7 | Ras-related protein Rab-1A | 75.98 | 67.32 | 4 | 1.15 | 1.21 | 1.09 | 1.05 | 1.13 | 0.07 | 22.66 | 6.21 | Rab1A | 81754 | Q6NYB7 | 51338716 | -0.294 | 1.103 | 7,13 |  | | | | | | | | | | | | | | | | | |
| G3V6H0 | Protein LOC100363782 | 98.54 | 83.08 | 8 | 1.2 | 1.04 | 1.16 | 1.11 | 1.13 | 0.07 | 22.16 | 5.73 | LOC100363782 | 100126191,100363782 | NP_001103449 | 158341664 | -0.305 | 1.579 |  |  | | | | | | | | | | | | | | | | | |
| A1L1J8 | Protein Rab5b | 14.02 | 14.42 | 2 |  |  | 1.19 | 1.08 | 1.13 |  | 23.66 | 8.13 | Rab5b | 288779 | NP_001073405 | 121583768 | -0.39 | 0.296 | 7,1 |  | | | | | | | | | | | | | | | | | |
| Q9Z270 | Vesicle-associated membrane protein-associated protein A | 54.84 | 53.82 | 11 | 1.15 | 1.16 | 1.13 | 1.13 | 1.14 | 0.02 | 27.82 | 8.4 | Vapa | 58857 | Q9Z270 | 122066704 | -0.396 | 0.827 | 1 |  | | | | | | | | | | | | | | | | | |
| Q6AXT5 | Ras-related protein Rab-21 | 29.37 | 28.25 | 5 | 1.14 | 1.12 | 1.17 | 1.18 | 1.13 | 0.03 | 24.15 | 7.94 | Rab21 | 299799 | Q6AXT5 | 81884468 | -0.343 | 0.373 |  |  | | | | | | | | | | | | | | | | | |
| Q641Z6 | EH domain-containing protein 1 | 38.19 | 22.1 | 6 | 1.18 | 1.23 | 1.16 | 1.12 | 1.17 | 0.04 | 60.56 | 6.83 | Ehd1 | 293692 | Q641Z6 | 81910618 | -0.363 | 0.231 |  |  | | | | | | | | | | | | | | | | | |
| G3V7P1 | Syntaxin-12 | 5.69 | 9.49 | 2 |  |  | 1.08 | 1.27 | 1.17 |  | 31.17 | 5.33 | Stx12 | 65033 | G3V7P1 | 378524692 | -0.6 | 0.064 |  |  | | | | | | | | | | | | | | | | | |
| P05712 | Ras-related protein Rab-2A | 61.57 | 54.72 | 11 | 1.16 | 1.26 | 1.2 | 1.11 | 1.18 | 0.06 | 23.52 | 6.54 | Rab2a | 65158 | P05712 | 131789 | -0.377 | 0.978 |  |  | | | | | | | | | | | | | | | | | |
| P09527 | Ras-related protein Rab-7a | 60.28 | 63.29 | 13 | 1.19 | 1.17 | 1.17 | 1.31 | 1.21 | 0.07 | 23.49 | 6.7 | Rab7a | 29448 | P09527 | 1710001 | -0.377 | 0.894 |  |  | | | | | | | | | | | | | | | | | |
| Q9Z269 | Vesicle-associated membrane protein-associated protein B | 68.97 | 42.39 | 7 | 1.16 | 1.19 | 1.28 | 1.32 | 1.24 | 0.08 | 26.9 | 7.78 | Vapb | 60431 | Q9Z269 | 24638336 | -0.316 | 0.706 |  |  | | | | | | | | | | | | | | | | | |
| O35509 | Ras-related protein Rab-11B | 35.25 | 38.99 | 7 |  |  | 1.22 | 1.25 | 1.24 |  | 24.47 | 5.94 | Rab11b | 79434 | O35509 | 50403673 | -0.435 | 0.49 | 1 |  | | | | | | | | | | | | | | | | | |
| Q5RKJ9 | RAB10, member RAS oncogene family | 45.31 | 39 | 6 | 1.25 | 1.31 | 1.29 | 1.11 | 1.28 | 0.09 | 22.53 | 8.38 | Rab10 | 50993 | Q5RKJ9 | 81910044 | -0.33 | 0.799 | 4,7,13,1 |  | | | | | | | | | | | | | | | | | |
| P35053 | Glypican-1 | 46.08 | 25.09 | 11 | 1.28 | 1.37 | 1.22 | 1.29 | 1.29 | 0.06 | 61.69 | 7.21 | Gpc1 | 58920 | P35053 | 462191 | -0.365 | 0.211 |  |  | | | | | | | | | | | | | | | | | |
| Q8R3Z7 | EH-domain containing 4 | 29.79 | 20.7 | 7 | 1.28 | 1.36 | 1.14 | 1.41 | 1.3 | 0.12 | 61.43 | 6.76 | Ehd4 | 192204 | Q8R3Z7 | 81915041 | -0.392 | 0.228 | 1 |  | | | | | | | | | | | | | | | | | |
| P51638 | Caveolin-3 | 14.73 | 17.22 | 2 | 1.34 | 1.33 | 1.4 | 1.44 | 1.38 | 0.05 | 17.39 | 5.81 | Cav3 | 29161 | P51638 | 1705649 | 0.339 | 0.575 |  |  | | | | | | | | | | | | | | | | | |
| Q5EB77 | Ras-related protein Rab-18 | 24.83 | 28.64 | 5 | 1.45 | 1.42 | 1.42 | 1.4 | 1.42 | 0.02 | 22.96 | 5.24 | Rab18 | 307039 | Q5EB77 | 81909560 | -0.308 | 0.436 | 4,7,12,13,1 |  | | | | | | | | | | | | | | | | | |
| D4A6X1 | Dysferlin (Predicted), isoform CRA_a | 532.4 | 46.72 | 80 | 1.62 | 1.64 | 1.74 | 1.56 | 1.64 | 0.08 | 230.8 | 5.68 | Dysf | 312492 | NP_001101339 | 157823277 | -0.396 | 0.741 | 1 |  | | | | | | | | | | | | | | | | | |
| O35987 | NSFL1 cofactor p47 | 9.25 | 8.92 | 2 | 1.79 | 1.84 | 1.87 | 1.76 | 1.81 | 0.05 | 40.66 | 5.15 | Nsfl1c | 83809 | O35987 | 41017427 | -0.638 | 0.074 |  |  | | | | | | | | | | | | | | | | | |
| **DNA replication, transcription and translation** | | | | | | | | | | | | | | | | | | |  | |  |  |  |  |  |  |  |  |  |  |  |  |  |  |  |  |  |
| G3V8L9 | Polymerase I and transcript release factor | 232.6 | 53.06 | 29 | 0.54 | 0.45 | 0.55 | 0.49 | 0.51 | 0.05 | 43.88 | 5.52 | Ptrf | 287710 | NP_001099311 | 157786694 | -0.758 | 2.21 | 7 |  | | | | | | | | | | | | | | | | | |
| Q66H98 | Serum deprivation-response protein | 60.76 | 28.54 | 12 | 0.5 | 0.62 | 0.58 | 0.6 | 0.58 | 0.05 | 46.36 | 5.29 | Sdpr | 316384 | Q66H98 | 81884184 | -0.923 | 0.561 |  |  | | | | | | | | | | | | | | | | | |
| Q6AY48 | Poly(RC) binding protein 3 | 11.69 | 7.84 | 2 |  |  | 0.63 | 0.68 | 0.65 |  | 33.76 | 8.51 | Pcbp3 | 294336 | Q6AY48 | 81884522 | -0.114 | 0.148 |  |  | | | | | | | | | | | | | | | | | |
| Q6AYU5 | Poly(RC) binding protein 2 | 19.51 | 15.89 | 4 | 0.59 | 0.71 |  |  | 0.65 |  | 38.56 | 6.79 | Pcbp2 | 363005 | Q6AYU5 | 81884660 | -0.137 | 0.207 | 1 |  | | | | | | | | | | | | | | | | | |
| F1LYI5 | Protein RGD1564138 | 8.87 | 12.93 | 2 | 0.52 | 0.63 | 0.77 | 0.84 | 0.69 | 0.14 | 16.4 | 11.43 | RGD1564138 | 29283 | NP_058846 | 8394209 | -1.018 | 0.244 |  |  | | | | | | | | | | | | | | | | | |
| Q5RJQ4 | NAD-dependent protein deacetylase sirtuin-2 | 11.06 | 6.86 | 2 |  |  | 0.82 | 0.67 | 0.75 |  | 39.29 | 7.08 | Sirt2 | 361532 | Q5RJQ4 | 81883338 | -0.305 | 0.051 |  |  | | | | | | | | | | | | | | | | | |
| Q9EQX9 | Ubiquitin-conjugating enzyme E2 N | 9.07 | 25.66 | 5 | 0.69 | 0.81 | 0.82 | 0.81 | 0.75 | 0.06 | 17.11 | 6.57 | Ube2n | 116725 | Q9EQX9 | 77417616 | -0.272 | 0.409 | 7,13,15,1 |  | | | | | | | | | | | | | | | | | |
| Q63797 | Proteasome activator complex subunit 1 | 19.99 | 15.26 | 3 | 0.9 | 0.78 | 0.72 | 0.75 | 0.79 | 0.08 | 28.56 | 6.02 | Psme1 | 29630 | Q63797 | 18202600 | -0.641 | 0.245 |  |  | | | | | | | | | | | | | | | | | |
| F1LQW3 | Protein Sfpq | 15.88 | 9.41 | 3 | 0.92 | 0.76 | 0.74 | 0.75 | 0.79 | 0.09 | 65.65 | 8.81 | Sfpq | 252855 | NP_001020442 | 70778983 | -1.055 | 0.076 |  |  | | | | | | | | | | | | | | | | | |
| Q5BJP4 | Protein LOC100363776 | 5.83 | 3.63 | 2 | 0.89 | 0.77 | 0.87 | 0.81 | 0.83 | 0.08 | 58.65 | 10.13 | Rbm39 | 100910882,362251 | Q5BJP4 | 81909463 | -0.681 | 0.034 |  |  | | | | | | | | | | | | | | | | | |
| Q6AYJ9 | ADP-ribosyltransferase 3 | 35.43 | 25.67 | 8 | 0.81 | 0.84 | 0.79 | 0.89 | 0.83 | 0.04 | 41.69 | 5.9 | Art3 | 305235 | Q6AYJ9 | 81884613 | -0.299 | 0.288 |  |  | | | | | | | | | | | | | | | | | |
| P43278 | Histone H1.0 | 8.44 | 13.92 | 3 | 0.86 | 0.84 | 0.85 | 0.83 | 0.84 | 0.01 | 20.87 | 10.9 | H1f0 | 24437 | P43278 | 1170150 | -1.053 | 0.192 | 1 |  | | | | | | | | | | | | | | | | | |
| Q9WTT7 | Basic leucine zipper and W2 domain-containing protein 2 | 8.9 | 6.68 | 2 | 0.79 | 0.92 | 0.83 | 0.86 | 0.85 | 0.06 | 48.02 | 6.68 | Bzw2 | 171439 | Q9WTT7 | 81882072 | -0.385 | 0.042 |  |  | | | | | | | | | | | | | | | | | |
| Q68FU7 | Ubiquinone biosynthesis monooxygenase COQ6 | 19.5 | 10.29 | 4 | 0.83 | 0.94 | 0.82 | 0.86 | 0.86 | 0.05 | 51.46 | 7.21 | Coq6 | 299195 | Q68FU7 | 81884362 | 0.028 | 0.117 |  |  | | | | | | | | | | | | | | | | | |
| Q641Y0 | Dolichyl-diphosphooligosaccharide--protein glycosyltransferase 48 kDa subunit | 5.04 | 6.8 | 3 | 0.76 | 1.01 | 0.85 | 0.85 | 0.87 | 0.1 | 48.86 | 5.97 | Ddost | 313648 | Q641Y0 | 81884080 | -0.003 | 0.061 | 1 |  | | | | | | | | | | | | | | | | | |
| Q5FVM4 | Non-POU domain-containing octamer-binding protein | 15.04 | 10.29 | 5 | 0.91 | 0.96 | 0.73 | 0.89 | 0.87 | 0.1 | 54.89 | 8.95 | Nono | 317259 | Q5FVM4 | 67460593 | -1.038 | 0.109 |  |  | | | | | | | | | | | | | | | | | |
| D4A9P9 | Gup1, glycerol uptake/transporter homolog (Yeast) (Predicted) | 17.63 | 4.97 | 3 | 0.97 | 0.9 | 0.86 | 0.86 | 0.9 | 0.05 | 56.34 | 6.6 | Hhatl | 301073 | NP_001100338 | 157822045 | 0.357 | 0.142 | 1 |  | | | | | | | | | | | | | | | | | |
| P25235 | Dolichyl-diphosphooligosaccharide--protein glycosyltransferase subunit 2 | 6.72 | 5.39 | 2 |  |  | 0.91 | 0.89 | 0.9 |  | 69.04 | 5.97 | Rpn2 | 64701 | P25235 | 62512124 | 0.09 | 0.043 |  |  | | | | | | | | | | | | | | | | | |
| D3ZJ08 | Histone H3 | 25.82 | 33.82 | 8 | 1.08 | 0.8 | 0.92 | 0.8 | 0.94 | 0.13 | 15.38 | 11.27 | Hist2h3c2 | 310678,684841 | NP_001101168 | 157818935 | -0.604 | 0.91 |  |  | | | | | | | | | | | | | | | | | |
| P35427 | 60S ribosomal protein L13a | 11.32 | 16.26 | 4 | 1.01 | 0.85 | 0.99 | 0.83 | 0.92 | 0.09 | 23.46 | 11.02 | Rpl13a | 317646 | P35427 | 548747 | -0.443 | 0.298 | 15 |  | | | | | | | | | | | | | | | | | |
| P62804 | Histone H4 | 58.98 | 53.4 | 8 | 0.9 | 0.95 | 0.94 | 0.88 | 0.92 | 0.03 | 11.36 | 11.36 | Hist1h4b | 100360950,102551184,291152,295277,64627,680097 | P62804 | 51317315 | -0.521 | 1.849 |  |  | | | | | | | | | | | | | | | | | |
| Q6P3V8 | Eukaryotic translation initiation factor 4A1 | 14.86 | 10.1 | 3 | 0.85 | 1 | 0.93 | 0.91 | 0.92 | 0.06 | 46.12 | 5.48 | Eif4a1 | 287436 | Q6P3V8 | 81892198 | -0.261 | 0.087 | 1 |  | | | | | | | | | | | | | | | | | |
| Q00715 | Histone H2B type 1 | 57.33 | 62.4 | 10 | 1.08 | 0.86 | 0.9 | 0.86 | 0.93 | 0.11 | 13.98 | 10.36 | Hist1h2ba | 64647 | Q00715 | 399856 | -0.835 | 1.931 |  |  | | | | | | | | | | | | | | | | | |
| P83941 | Transcription elongation factor B polypeptide 1 | 9.07 | 31.25 | 3 | 0.97 | 0.93 | 0.86 | 0.96 | 0.93 | 0.05 | 12.46 | 4.78 | Tceb1 | 64525 | P83941 | 48428883 | -0.199 | 0.241 | 1 |  | | | | | | | | | | | | | | | | | |
| Q6P7A7 | Dolichyl-diphosphooligosaccharide--protein glycosyltransferase subunit 1 | 30.38 | 18.81 | 10 | 0.92 | 0.96 | 0.92 | 0.92 | 0.93 | 0.02 | 68.36 | 6.51 | Rpn1 | 25596 | Q6P7A7 | 81885337 | -0.226 | 0.161 |  |  | | | | | | | | | | | | | | | | | |
| P13471 | 40S ribosomal protein S14 | 22.97 | 43.05 | 5 | 0.95 | 0.93 | 0.96 | 0.89 | 0.94 | 0.03 | 16.25 | 10.05 | Rps14 | 29284 | P13471 | 133785 | -0.503 | 0.739 | 15 |  | | | | | | | | | | | | | | | | | |
| Q63798 | Proteasome activator complex subunit 2 | 7.31 | 13.03 | 2 | 1.01 | 1.07 | 0.87 | 0.78 | 0.93 | 0.13 | 26.84 | 5.72 | Psme2 | 29614 | Q63798 | 18202601 | -0.268 | 0.075 |  |  | | | | | | | | | | | | | | | | | |
| Q3V5X8 | Endonuclease G | 7.99 | 17.01 | 5 | 0.93 | 1.01 | 0.89 | 0.97 | 0.95 | 0.05 | 32.25 | 9.51 | Endog | 362100 | Q3V5X8 | 123781243 | -0.292 | 0.248 |  |  | | | | | | | | | | | | | | | | | |
| B0BN81 | Ribosomal protein S5, isoform CRA_b | 6.72 | 13.24 | 3 | 1.01 | 0.89 |  |  | 0.95 |  | 22.89 | 9.72 | Rps5 | 25538 | NP_001264172 | 471009907 | -0.393 | 0.175 | 3,16 |  | | | | | | | | | | | | | | | | | |
| F1M9N5 | Ubiquitin conjugation factor E4 A | 7.03 | 3.47 | 4 | 0.9 | 1.16 | 0.82 | 0.92 | 0.95 | 0.15 | 122.4 | 5.25 | Ube4a | 315608 | NP_997493 | 46485190 | -0.22 | 0.033 | 7,13,15,1 |  | | | | | | | | | | | | | | | | | |
| D3ZZC1 | Protein Txndc5 | 9.68 | 8.63 | 3 | 1.1 | 0.89 | 0.89 | 0.95 | 0.96 | 0.1 | 46.32 | 5.88 | Txndc5 | 100362805 | NP_001258259 | 404501500 | -0.438 | 0.108 |  |  | | | | | | | | | | | | | | | | | |
| P61023 | Calcineurin B homologous protein 1 | 49.54 | 63.59 | 11 | 0.93 | 0.93 | 0.99 | 0.99 | 0.96 | 0.04 | 22.42 | 5.1 | Chp1 | 64152 | P61023 | 46577577 | -0.626 | 0.758 |  |  | | | | | | | | | | | | | | | | | |
| F1M0Q9 | Protein Pm20d1 | 7.64 | 6.44 | 3 | 1.02 | 1.03 | 0.82 | 0.97 | 1.02 | 0.1 | 46.59 | 6.9 | Pm20d1 | 498226 | NP_001102538 | 157818365 | -0.018 | 0.064 |  |  | | | | | | | | | | | | | | | | | |
| P29314 | 40S ribosomal protein S9 | 3.77 | 8.25 | 2 |  |  | 0.91 | 1.02 | 0.96 |  | 22.58 | 10.65 | Rps9 | 81772 | P29314 | 52788199 | -0.644 | 0.089 | 15 |  | | | | | | | | | | | | | | | | | |
| P11598 | Protein disulfide-isomerase A3 | 153.4 | 57.03 | 27 | 1.01 | 0.94 | 0.98 | 0.96 | 0.97 | 0.03 | 56.59 | 6.21 | Pdia3 | 29468 | P11598 | 1352384 | -0.455 | 1.007 |  |  | | | | | | | | | | | | | | | | | |
| P05197 | Elongation factor 2 | 7.06 | 4.08 | 3 | 0.95 | 1.01 |  |  | 0.98 |  | 95.22 | 6.83 | Eef2 | 29565 | P05197 | 119176 | -0.206 | 0.042 |  |  | | | | | | | | | | | | | | | | | |
| P62856 | 40S ribosomal protein S26 | 6.75 | 26.96 | 3 |  |  | 0.94 | 1.03 | 0.99 |  | 13.01 | 11 | Rps26 | 27139 | P62856 | 51338622 | -0.66 | 0.231 | 15 |  | | | | | | | | | | | | | | | | | |
| P24368 | Peptidyl-prolyl cis-trans isomerase B | 30.48 | 28.7 | 6 | 0.89 | 1.02 | 0.94 | 1.11 | 0.99 | 0.09 | 23.79 | 9.5 | Ppib | 64367 | P24368 | 215274190 | -0.202 | 0.462 |  |  | | | | | | | | | | | | | | | | | |
| A0JPQ4 | Tripartite motif-containing protein 72 | 102.8 | 49.48 | 18 | 0.93 | 1.04 | 0.95 | 1.04 | 0.99 | 0.06 | 52.8 | 6.27 | Trim72 | 365377 | A0JPQ4 | 126253817 | -0.243 | 0.625 | 1 |  | | | | | | | | | | | | | | | | | |
| Q63081 | Protein disulfide-isomerase A6 | 55.46 | 32.5 | 9 | 1.04 | 0.99 | 0.97 | 0.97 | 1.01 | 0.03 | 48.14 | 5.14 | Pdia6 | 286906 | Q63081 | 62296810 | -0.311 | 0.353 |  |  | | | | | | | | | | | | | | | | | |
| D3ZFA8 | Protein LOC100362366 | 13.03 | 31.11 | 3 | 1.08 | 1.02 | 0.85 | 1.02 | 0.99 | 0.1 | 15.46 | 9.77 | LOC100364909 | 29286 | NP_058848 | 297515469 | -0.584 | 0.194 |  |  | | | | | | | | | | | | | | | | | |
| M0R5K9 | Protein LOC100912024 (Fragment) | 7.91 | 15.23 | 4 |  |  | 1.05 | 0.94 | 1 |  | 17.64 | 11.11 | LOC100912024 | 294282 | NP_998722 | 47087103 | -0.696 | 0.34 |  |  | | | | | | | | | | | | | | | | | |
| Q9JLA3 | UDP-glucose:glycoprotein glucosyltransferase 1 | 5 | 1.42 | 2 |  |  | 1.04 | 0.96 | 1 |  | 176.3 | 5.66 | Uggt1 | 171129 | Q9JLA3 | 224471866 | -0.317 | 0.011 |  |  | | | | | | | | | | | | | | | | | |
| P12001 | 60S ribosomal protein L18 | 16.61 | 30.32 | 5 | 1.18 | 0.94 | 0.95 | 0.92 | 1 | 0.12 | 21.65 | 11.78 | Rpl18 | 81766 | P12001 | 132733 | -0.718 | 0.277 | 15 |  | | | | | | | | | | | | | | | | | |
| Q5U2Q8 | Asph protein | 26 | 25.12 | 6 | 1 | 0.99 | 1.04 | 0.97 | 1 | 0.03 | 22.62 | 9.7 | Asph | 312981 | Q5U2Q8 | 81883480 | -1.159 | 0.442 |  |  | | | | | | | | | | | | | | | | | |
| B0K031 | 60S ribosomal protein L7 | 27.09 | 26.15 | 7 | 1.04 | 1.04 | 1 | 0.95 | 1.01 | 0.04 | 30.29 | 10.87 | Rpl7 | 297755 | NP_001094004 | 198278505 | -0.578 | 0.528 |  |  | | | | | | | | | | | | | | | | | |
| P54921 | Alpha-soluble NSF attachment protein | 9.8 | 14.24 | 4 |  |  | 0.96 | 1.06 | 0 |  | 33.17 | 5.45 | Napa | 140673 | P54921 | 6094309 | -0.355 | 0.121 |  |  | | | | | | | | | | | | | | | | | |
| P23358 | 60S ribosomal protein L12 | 19.5 | 35.76 | 4 | 1.05 | 0.97 |  |  | 1.01 |  | 17.83 | 9.42 | Rpl12 | 102555453,499782 | P23358 | 132653 | -0.363 | 0.449 | 15 |  | | | | | | | | | | | | | | | | | |
| P35565 | Calnexin | 72.85 | 21.32 | 13 | 1.03 | 1.03 | 1.02 | 0.98 | 1.01 | 0.02 | 67.21 | 4.63 | Canx | 29144 | P35565 | 543922 | -0.896 | 0.446 | 2,7,8,12,15,1 |  | | | | | | | | | | | | | | | | | |
| M0R763 | Protein LOC100911337 | 7.86 | 25.6 | 4 |  |  | 1.03 | 1.01 | 1.02 |  | 13.76 | 10.11 | LOC100911337 | 122799 | NP_001005528 | 53850582 | -0.815 | 0.291 |  |  | | | | | | | | | | | | | | | | | |
| Q5RJR9 | Serine (Or cysteine) proteinase inhibitor, clade H, member 1, isoform CRA_b | 89.93 | 44.84 | 15 | 1.03 | 1.09 | 0.99 | 0.99 | 1.03 | 0.05 | 46.53 | 8.82 | Serpinh1 | 29345 | Q5RJR9 | 81889407 | -0.294 | 0.537 | 15 |  | | | | | | | | | | | | | | | | | |
| Q99ML5 | Prenylcysteine oxidase | 22.07 | 11.9 | 3 | 0.94 | 1.16 | 1.02 | 0.98 | 1.03 | 0.1 | 56.25 | 7.09 | Pcyox1 | 246302 | Q99ML5 | 62286984 | -0.099 | 0.089 |  |  | | | | | | | | | | | | | | | | | |
| P62919 | 60S ribosomal protein L8 | 21.22 | 31.13 | 7 | 1.09 | 1.05 | 1 | 0.97 | 1.03 | 0.05 | 28.01 | 11.03 | Rpl8 | 100360117,100910370,26962 | P62919 | 51702822 | -0.528 | 0.357 |  |  | | | | | | | | | | | | | | | | | |
| P0CG51 | Polyubiquitin-B | 36.88 | 69.51 | 5 |  |  | 0.99 | 1.07 | 1.03 |  | 34.35 | 7.53 | Ubb | 192255 | P0CG51 | 302595879 | -0.492 | 0.349 |  |  | | | | | | | | | | | | | | | | | |
| M0RA26 | Protein LOC100362987 | 5.38 | 25 | 2 |  |  | 1.12 | 0.95 | 1.03 | 0.12 | 9.51 | 9.7 | LOC100362987 | 100362987 | XP_002727201 | 293349609 | -0.617 | 0.21 |  |  | | | | | | | | | | | | | | | | | |
| Q6IMY8 | Heterogeneous nuclear ribonucleoprotein U | 9.2 | 4.89 | 4 | 1.16 | 0.97 | 1.01 | 1.01 | 1.04 | 0.08 | 87.68 | 6.24 | Hnrnpu | 117280 | Q6IMY8 | 81884791 | -0.926 | 0.057 |  |  | | | | | | | | | | | | | | | | | |
| D3Z9G3 | Protein LOC100361103 | 11.07 | 23.13 | 5 | 1.28 | 0.88 | 1.13 | 0.88 | 1.04 | 0.2 | 18.57 | 10.55 | LOC100361103 | 79449 | AAH58459 | 37231655 | -0.894 | 0.323 |  |  | | | | | | | | | | | | | | | | | |
| D3ZZK1 | Protein LOC100359563 | 6.91 | 30.25 | 3 | 1 | 1.06 | 1.12 | 0.99 | 1.05 | 0.06 | 13.35 | 9.8 | LOC100359563 | 100359563 |  |  | -0.385 | 0.3 |  |  | | | | | | | | | | | | | | | | | |
| P22062 | Protein-L-isoaspartate(D-aspartate) O-methyltransferase | 7.42 | 11.01 | 2 | 1.08 | 1.12 | 0.98 | 1 | 1.05 | 0.07 | 24.63 | 7.68 | Pcmt1 | 25604 | P22062 | 124106314 | -0.167 | 0.081 | 15 |  | | | | | | | | | | | | | | | | | |
| D3ZX01 | Protein Rps4y2 | 11.99 | 12.6 | 3 |  |  | 1.15 | 0.96 | 1.05 |  | 29.18 | 10.27 | Rps4y2 | 690845 | NP_001103082 | 157819705 | -0.317 | 0.171 |  |  | | | | | | | | | | | | | | | | | |
| P62832 | 60S ribosomal protein L23 | 12.8 | 34.29 | 4 | 1.04 | 1 | 1.04 | 1.14 | 1.02 | 0.06 | 14.86 | 10.51 | Rpl23 | 29282 | P62832 | 51338617 | -0.194 | 0.337 | 15 |  | | | | | | | | | | | | | | | | | |
| B2RYP6 | LUC7-like 2 (S. cerevisiae) | 8.48 | 6.89 | 3 | 1.08 | 0.99 | 1.08 | 1.08 | 1.06 | 0.05 | 46.55 | 10.1 | Luc7l2 | 312251 | NP_001101323 | 157822829 | -1.511 | 0.086 |  |  | | | | | | | | | | | | | | | | | |
| P50878 | 60S ribosomal protein L4 | 43.64 | 24.7 | 10 | 1.08 | 0.93 | 1.11 | 1.1 | 1.06 | 0.08 | 47.23 | 10.92 | Rpl4 | 64302 | P50878 | 1710511 | -0.634 | 0.339 | 15 |  | | | | | | | | | | | | | | | | | |
| M0R7P0 | Protein LOC100912027 | 4.57 | 30.15 | 3 | 1.03 | 1.09 |  |  | 1.06 |  | 15.81 | 10.56 | LOC100912027 | 64306 | NP_071959 | 11968094 | -0.665 | 0.253 |  |  | | | | | | | | | | | | | | | | | |
| P05765 | 40S ribosomal protein S21 | 16.65 | 30.12 | 3 | 1.1 | 0.88 | 1.38 | 0.89 | 1.06 | 0.23 | 9.12 | 8.51 | Rps21 | 81775 | P05765 | 133879 | -0.352 | 0.658 | 15 |  | | | | | | | | | | | | | | | | | |
| P62982 | Ubiquitin-40S ribosomal protein S27a | 45.29 | 54.49 | 7 | 1.01 | 1.12 |  |  | 1.06 |  | 17.94 | 9.64 | Rps27a | 100912032,81777 | P62982 | 302393757 | -0.858 | 0.78 |  |  | | | | | | | | | | | | | | | | | |
| F7FLF2 | Protein LOC100360057 (Fragment) | 7.84 | 16.54 | 3 | 1.05 | 1.04 | 1.04 | 1.12 | 1.06 | 0.04 | 14.65 | 9.19 | Rpl22 | 100360057 | XP_003753964 | 392340011 | -0.815 | 0.341 |  |  | | | | | | | | | | | | | | | | | |
| P85834 | Elongation factor Tu, mitochondrial | 141.2 | 67.26 | 23 | 1.08 | 1.04 | 1.04 | 1.11 | 1.06 | 0.03 | 49.49 | 7.56 | Tufm | 293481 | P85834 | 190359305 | -0.179 | 1.03 | 5,7,15,16,1 |  | | | | | | | | | | | | | | | | | |
| B1PRL5 | Muscle-related coiled-coil protein | 91.42 | 57.18 | 18 | 1.08 | 1.06 | 1.13 | 1.03 | 1.07 | 0.04 | 41.07 | 8.63 | Murc | 313225 | B1PRL5 | 226711873 | -0.89 | 0.852 |  |  | | | | | | | | | | | | | | | | | |
| G3V8V6 | O-acetyl-ADP-ribose deacetylase MACROD1 | 29.19 | 33.88 | 6 | 1 | 1.16 | 1.07 | 1.06 | 1.07 | 0.06 | 27.12 | 9.03 | Macrod1 | 246233 | AAP97291 | 33150826 | -0.35 | 0.332 |  |  | | | | | | | | | | | | | | | | | |
| P61314 | 60S ribosomal protein L15 | 28.69 | 33.82 | 6 | 1.05 | 1.04 | 1.17 | 1.05 | 1.08 | 0.06 | 24.13 | 11.62 | Rpl15 | 245981 | P61314 | 47117719 | -0.93 | 0.456 | 15 |  | | | | | | | | | | | | | | | | | |
| H7C5Y5 | 60S ribosomal protein L6 | 31.65 | 37.37 | 10 | 1.02 | 1.05 | 1.25 | 1 | 1.08 | 0.11 | 33.63 | 10.7 | Rpl6 | 117042 | CAA60588 | 1490384 | -0.774 | 0.595 | 15 |  | | | | | | | | | | | | | | | | | |
| P49242 | 40S ribosomal protein S3a | 36.65 | 40.53 | 9 | 1.09 | 1.01 | 1.05 | 1.17 | 1.08 | 0.07 | 29.93 | 9.73 | Rps3a | 29288 | P49242 | 1350987 | -0.626 | 0.601 | 15 |  | | | | | | | | | | | | | | | | | |
| F1LSW7 | 60S ribosomal protein L14 | 21.54 | 27.1 | 7 | 1.07 | 1.18 | 1.01 | 1.1 | 1.09 | 0.07 | 23.31 | 11.11 | Rpl14 | 65043 | NP_075238.2 | 2500360 | -0.502 | 0.429 |  |  | | | | | | | | | | | | | | | | | |
| P83732 | 60S ribosomal protein L24 | 7.62 | 17.83 | 3 | 1.24 | 1.02 | 1.1 | 1.01 | 1.13 | 0.11 | 17.77 | 11.25 | Rpl24 | 64307 | P83732 | 41019239 | -0.926 | 0.169 | 15 |  | | | | | | | | | | | | | | | | | |
| P62268 | 40S ribosomal protein S23 | 15.41 | 25.87 | 5 | 1.09 | 0.98 | 1.17 | 1.13 | 1.09 | 0.08 | 15.8 | 10.49 | Rps23 | 124323 | P62268 | 50403678 | -0.519 | 0.443 | 15 |  | | | | | | | | | | | | | | | | | |
| G3V6I9 | 60S ribosomal protein L26 | 14.5 | 37.24 | 7 | 1.09 | 1.18 | 1.1 | 1.02 | 1.1 | 0.07 | 17.25 | 10.55 | Rpl26 | 287417 | XP_006246668 | 564372554 | -1.107 | 0.986 |  |  | | | | | | | | | | | | | | | | | |
| P62907 | 60S ribosomal protein L10a | 18.27 | 23.96 | 4 | 1.08 | 1.11 | 1.16 | 1.05 | 1.1 | 0.05 | 24.82 | 9.94 | Rpl10a | 81729 | P62907 | 51702767 | -0.408 | 0.282 | 15 |  | | | | | | | | | | | | | | | | | |
| P62859 | 40S ribosomal protein S28 | 11.68 | 46.38 | 3 |  |  | 1.04 | 1.16 | 1.1 |  | 7.84 | 10.7 | Rps28 | 691531 | P62859 | 51338623 | -0.594 | 0.51 | 15 |  | | | | | | | | | | | | | | | | | |
| P24049 | 60S ribosomal protein L17 | 16.2 | 25 | 5 | 1.2 | 1.15 | 1.07 | 1.01 | 1.11 | 0.09 | 21.38 | 10.18 | Rpl17 | 291434 | P24049 | 132805 | -0.903 | 0.421 |  |  | | | | | | | | | | | | | | | | | |
| P84100 | 60S ribosomal protein L19 | 5.53 | 16.84 | 4 | 1.31 | 1.09 | 1.12 | 0.96 | 1.2 | 0.14 | 23.45 | 11.47 | Rpl19 | 81767 | P84100 | 51338626 | -1.111 | 0.171 | 15 |  | | | | | | | | | | | | | | | | | |
| D3ZJD3 | Protein RGD1565183 | 16.1 | 30.66 | 5 | 1.13 | 1.19 | 1.11 | 1.05 | 1.12 | 0.06 | 15.56 | 11.93 | RGD1565183 | 64638 | NP_073188 | 61889083 | -0.689 | 0.45 |  |  | | | | | | | | | | | | | | | | | |
| G3V912 | Protein Tmx4 | 15.65 | 11.31 | 3 | 1.17 | 1.11 | 1.05 | 1.16 | 1.12 | 0.06 | 37.52 | 4.35 | Tmx4 | 296182 | NP_001093999 | 209915579 | -0.456 | 0.133 |  |  | | | | | | | | | | | | | | | | | |
| M0R9L0 | Protein Naca | 16.18 | 2.08 | 3 | 1.19 | 1.05 |  |  | 1.12 |  | 220.1 | 9.32 | Naca | 288770 | NP_001185509 | 310703617 | -0.366 | 0.018 |  |  | | | | | | | | | | | | | | | | | |
| D4A6G6 | Protein LOC100362339 | 31.84 | 37.24 | 7 | 1.17 | 1.2 | 1.04 | 1.09 | 1.12 | 0.07 | 16.11 | 10.4 | LOC100362339 | 29287 | NP_001032423 | 82654220 | -0.671 | 0.683 |  |  | | | | | | | | | | | | | | | | | |
| D3ZDR2 | Chromatin modifying protein 6 (Predicted) | 9.24 | 9.5 | 2 | 1.07 | 1.06 | 1.04 | 1.34 | 1.13 | 0.14 | 23.27 | 5.31 | Chmp6 | 287873 | NP_001099326 | 157786724 | -0.844 | 0.129 |  |  | | | | | | | | | | | | | | | | | |
| P62632 | Elongation factor 1-alpha 2 | 63.11 | 31.53 | 5 | 1.15 | 1.15 | 1.18 | 1.06 | 1.15 | 0.06 | 50.42 | 9.03 | Eef1a2 | 24799 | P62632 | 50402096 | -0.3 | 0.416 | 5,7,15,16,1 |  | | | | | | | | | | | | | | | | | |
| D3ZIE1 | 40S ribosomal protein S8 | 8.76 | 18.27 | 3 | 1.28 | 1 |  |  | 1.14 |  | 24.14 | 10.14 | RGD1566369 | 65136 | NP_113894 | 13928986 | -0.993 | 0.373 | 15 |  | | | | | | | | | | | | | | | | | |
| Q5VLR5 | BWK4 | 8.97 | 9.85 | 4 | 0.96 | 1.1 | 1.22 | 1.28 | 1.14 | 0.14 | 46.85 | 5.27 | Erp44 | 298066 | Q5VLR5 | 81862689 | -0.413 | 0.085 |  |  | | | | | | | | | | | | | | | | | |
| P62845 | 40S ribosomal protein S15 | 7.9 | 22.76 | 3 | 1.14 | 1.19 | 1.22 | 1.02 | 1.14 | 0.09 | 17.03 | 10.39 | Rps15 | 29285 | P62845 | 51338619 | -0.714 | 0.352 | 15 |  | | | | | | | | | | | | | | | | | |
| D4A9G1 | Protein Rpl3l | 28.42 | 25.55 | 10 | 1.21 | 1.12 | 1.22 | 1.03 | 1.14 | 0.09 | 46.36 | 10.45 | Rpl3l | 287122 | NP_001178518 | 300797628 | -0.622 | 0.259 |  |  | | | | | | | | | | | | | | | | | |
| P62282 | 40S ribosomal protein S11 | 12.76 | 33.54 | 6 | 1.06 | 1.13 | 1.17 | 1.22 | 1.15 | 0.07 | 18.42 | 10.3 | Rps11 | 81774 | P62282 | 54039305 | -0.607 | 0.326 | 15 |  | | | | | | | | | | | | | | | | | |
| P62890 | 60S ribosomal protein L30 | 8.99 | 29.57 | 3 | 1.04 | 1.24 | 1.06 | 1.26 | 1.15 | 0.12 | 12.78 | 9.63 | Rpl30 | 100362027,64640 | P62890 | 51702801 | -0.295 | 0.313 | 15 |  | | | | | | | | | | | | | | | | | |
| F2Z3T9 | Protein U2af2 | 2.11 | 3.58 | 2 |  |  | 1.12 | 1.18 | 1.15 |  | 53.48 | 9.09 | U2af2 | 308335 | XP_003748811 | 392343893 | -0.578 | 0.037 |  |  | | | | | | | | | | | | | | | | | |
| Q6AYT3 | tRNA-splicing ligase RtcB homolog | 11.39 | 7.92 | 3 | 1 | 1.07 | 1.25 | 1.29 | 1.15 | 0.14 | 55.21 | 7.23 | Rtcb | 362855 | Q6AYT3 | 81884655 | -0.241 | 0.054 |  |  | | | | | | | | | | | | | | | | | |
| D3ZTH8 | Protein LOC689899 | 14.21 | 28.21 | 4 | 1.28 | 1 | 1.3 | 1.04 | 1.16 | 0.16 | 17.74 | 10.39 | LOC689899 | 360572 | NP_001101753 | 157818939 | -0.76 | 0.282 |  |  | | | | | | | | | | | | | | | | | |
| P62278 | 40S ribosomal protein S13 | 10.84 | 22.52 | 3 | 1.29 | 1.15 | 1.14 | 1.06 | 1.16 | 0.1 | 17.21 | 10.54 | Rps13 | 161477,684988 | P62278 | 54039303 | -0.454 | 0.232 | 15 |  | | | | | | | | | | | | | | | | | |
| P62914 | 60S ribosomal protein L11 | 15.35 | 26.97 | 6 |  |  | 1.17 | 1.15 | 1.16 |  | 20.24 | 9.6 | Rpl11 | 362631 | P62914 | 51702792 | -0.534 | 0.395 | 15 |  | | | | | | | | | | | | | | | | | |
| D3ZNZ8 | 40S ribosomal protein S27 | 2.03 | 23.81 | 2 | 1.1 | 1.23 |  |  | 1.16 |  | 9.46 | 9.28 | Rps27l2 | 94266 | NP_446049 | 16758382 | -0.467 | 0.211 | 15 |  | | | | | | | | | | | | | | | | | |
| D4A4D5 | Protein LOC100362751 | 35.91 | 69.57 | 5 | 1.2 | 1.22 | 1.17 | 1.07 | 1.16 | 0.07 | 11.7 | 4.54 | LOC498555 | 140662 | NP_001025192 | 71795613 | -0.263 | 0.855 |  |  | | | | | | | | | | | | | | | | | |
| P62703 | 40S ribosomal protein S4, X isoform | 13.97 | 13.69 | 4 | 1.33 | 1 |  |  | 1.16 |  | 29.58 | 10.15 | Rps4x | 100362640,29426 | P62703 | 50403621 | -0.33 | 0.169 | 15 |  | | | | | | | | | | | | | | | | | |
| P19945 | 60S acidic ribosomal protein P0 | 31.65 | 29.02 | 7 | 1.1 | 1.23 | 1.19 | 1.16 | 1.17 | 0.05 | 34.19 | 6.25 | Rplp0 | 64205 | P19945 | 730581 | 0.05 | 0.263 | 15 |  | | | | | | | | | | | | | | | | | |
| P63326 | 40S ribosomal protein S10 | 16.74 | 27.88 | 4 | 1.1 | 1.16 | 1.1 | 1.33 | 1.17 | 0.11 | 18.9 | 10.15 | Rps10 | 100363439,81773 | P63326 | 54039307 | -0.89 | 0.37 | 15 |  | | | | | | | | | | | | | | | | | |
| P09895 | 60S ribosomal protein L5 | 32.46 | 32.32 | 10 | 1.31 | 1.16 | 1.13 | 1.1 | 1.17 | 0.09 | 34.44 | 9.74 | Rpl5 | 81763 | P09895 | 1173056 | -0.765 | 0.494 | 15 |  | | | | | | | | | | | | | | | | | |
| P62243 | 40S ribosomal protein S8 | 13.38 | 30.77 | 5 |  |  | 1.39 | 0.96 | 1.18 |  | 24.19 | 10.32 | Rps8 | 65136 | P62243 | 54039527 | -1.02 | 0.413 | 15 |  | | | | | | | | | | | | | | | | | |
| D3ZRM9 | 60S ribosomal protein L13 | 25.32 | 34.6 | 9 | 1.23 | 1.15 | 1.28 | 1.04 | 1.18 | 0.1 | 24.19 | 11.41 | LOC100360491 | 100359922 | XP_008774860 | 672022500 | -0.826 | 0.496 | 15 |  | | | | | | | | | | | | | | | | | |
| P62755 | 40S ribosomal protein S6 | 32.87 | 35.34 | 8 | 1.19 | 1.19 | 1.28 | 1.08 | 1.18 | 0.08 | 28.66 | 10.84 | Rps6 | 100911372,29304 | P62755 | 51338614 | -0.945 | 0.454 | 15 |  | | | | | | | | | | | | | | | | | |
| P62853 | 40S ribosomal protein S25 | 8.81 | 29.6 | 4 | 1.19 | 1.18 |  |  | 1.18 |  | 13.73 | 10.11 | Rps25 | 122799 | P62853 | 51338621 | -0.861 | 0.291 | 15 |  | | | | | | | | | | | | | | | | | |
| P62909 | 40S ribosomal protein S3 | 20.84 | 27.57 | 5 | 1.18 | 1.26 | 1.1 | 1.2 | 1.18 | 0.07 | 26.66 | 9.66 | Rps3 | 140654 | P62909 | 51703302 | -0.153 | 0.263 | 15 |  | | | | | | | | | | | | | | | | | |
| P38983 | 40S ribosomal protein SA | 27.84 | 43.73 | 8 | 1.16 | 1.25 | 1.1 | 1.24 | 1.19 | 0.07 | 32.8 | 4.87 | Rpsa | 29236 | P38983 | 730681 | -0.308 | 0.305 | 15 |  | | | | | | | | | | | | | | | | | |
| Q6PDV7 | 60S ribosomal protein L10 | 15.4 | 22.9 | 5 | 1.23 | 1.21 | 1.18 | 1.2 | 1.21 | 0.02 | 24.59 | 10.08 | Rpl10 | 81764 | Q6PDV7 | 50403574 | -0.553 | 0.244 | 15 |  | | | | | | | | | | | | | | | | | |
| P62083 | 40S ribosomal protein S7 | 22.98 | 38.14 | 8 | 1.23 | 1.24 | 1.2 | 1.17 | 1.21 | 0.03 | 22.11 | 10.1 | Rps7 | 100362830,29258 | P62083 | 49065830 | -0.459 | 0.452 | 15 |  | | | | | | | | | | | | | | | | | |
| O55215 | Ribosomal protein S2 | 26.94 | 28.4 | 8 | 1.19 | 1.18 | 1.33 | 1.16 | 1.19 | 0.08 | 27.21 | 9.63 | LOC100360710 | 83789 | O55215 | 81886772 | -0.049 | 0.478 | 5 |  | | | | | | | | | | | | | | | | | |
| Q5XIG1 | Ldb3 protein | 6.17 | 12.72 | 2 | 1.14 | 1.15 | 1.23 | 1.34 | 1.22 | 0.09 | 30.97 | 9.19 | Ldb3 | 498587 | Q5XIG1 | 81889867 | -0.525 | 0.065 |  |  | | | | | | | | | | | | | | | | | |
| P19944 | 60S acidic ribosomal protein P1 | 26.66 | 51.75 | 2 | 1.37 | 1.11 | 1.38 | 1.08 | 1.23 | 0.16 | 11.49 | 4.32 | Rplp1 | 100360522,140661 | P19944 | 133053 | 0.132 | 0.435 | 15 |  | | | | | | | | | | | | | | | | | |
| P62250 | 40S ribosomal protein S16 | 8.59 | 26.03 | 4 | 1.25 | 1.23 | 1.25 | 1.21 | 1.23 | 0.02 | 16.44 | 10.21 | Rps16 | 140655 | P62250 | 54039370 | -0.445 | 0.304 | 15 |  | | | | | | | | | | | | | | | | | |
| M0R9I8 | 40S ribosomal protein S12 | 12.08 | 25.76 | 3 |  |  | 1.25 | 1.23 | 1.24 |  | 14.49 | 6.74 | LOC100364427 | 65139 | NP_113897 | 78126139 | -0.184 | 0.276 | 15 |  | | | | | | | | | | | | | | | | | |
| P62494 | Ras-related protein Rab-11A | 29.39 | 39.35 | 8 | 1.33 | 1.18 |  |  | 1.25 |  | 24.38 | 6.57 | Rab11a | 81830 | P62494 | 50402540 | -0.421 | 0.41 |  |  | | | | | | | | | | | | | | | | | |
| B2RYD0 | Protein Ube2g2 | 61.91 | 52.73 | 6 | 1.37 | 1.26 | 1.3 | 1.21 | 1.31 | 0.07 | 18.55 | 4.7 | Ube2g2 | 294331 | NP_001099850 | 157822469 | -0.205 | 0.97 |  |  | | | | | | | | | | | | | | | | | |
| P62850-2 | Isoform 2 of 40S ribosomal protein S24 | 6.22 | 16.92 | 2 |  |  | 1.32 | 1.25 | 1.29 |  | 15.06 | 10.89 | Rps24 | 81776 | NP_112374 | 13592075 | -0.995 | 0.133 | 15 |  | | | | | | | | | | | | | | | | | |
| P04785 | Protein disulfide-isomerase | 123.1 | 50.49 | 23 | 1.32 | 1.24 | 1.42 | 1.28 | 1.31 | 0.08 | 56.92 | 4.93 | P4hb | 25506 | P04785 | 129731 | -0.382 | 1.019 | 13 |  | | | | | | | | | | | | | | | | | |
| P45479 | Palmitoyl-protein thioesterase 1 | 22.48 | 13.73 | 4 | 1.51 | 1.21 | 1.39 | 1.24 | 1.34 | 0.14 | 34.43 | 7.43 | Ppt1 | 29411 | P45479 | 1172592 | -0.146 | 0.232 | 7,1 |  | | | | | | | | | | | | | | | | | |
| O88767 | Protein DJ-1 | 13.08 | 17.46 | 3 | 1.28 | 1.31 | 1.35 | 1.44 | 1.34 | 0.07 | 19.96 | 6.77 | Park7 | 117287 | O88767 | 56404680 | 0.022 | 0.2 |  |  | | | | | | | | | | | | | | | | | |
| M0R757 | Elongation factor 1-alpha | 53.03 | 29.87 | 4 | 1.46 | 1.49 | 1.47 | 1.56 | 1.49 | 0.05 | 50.08 | 9.01 | LOC100360413 | 171361 | NP_787032 | 28460696 | -0.244 | 0.359 | 5,7,15,16,1 |  | | | | | | | | | | | | | | | | | |
| Q6PDU1 | Serine/arginine-rich splicing factor 2 | 5.34 | 10.41 | 2 | 1.6 | 1.7 | 1.76 | 1.55 | 1.65 | 0.09 | 25.46 | 11.85 | Srsf2 | 494445 | Q6PDU1 | 52783335 | -1.624 | 0.079 |  |  | | | | | | | | | | | | | | | | | |
| G3V6I5 | Protein Dnaja3* | 102.3 | 39.17 | 17 | 0.7 | 0.76 | 0.74 | 0.8 | 0.73 | 0.04 | 52.37 | 9.28 | Dnaja3 | 360481 | XP_006245879 | 564370616 | -0.474 | 0.783 |  |  | | | | | | | | | | | | | | | | | |
| P34058 | Heat shock protein HSP 90-beta* | 97.09 | 31.49 | 12 | 0.84 | 0.76 | 0.74 | 0.81 | 0.79 | 0.04 | 83.23 | 5.03 | Hsp90ab1 | 301252 | P34058 | 122065211 | -0.678 | 0.457 | 4,5 |  | | | | | | | | | | | | | | | | | |
| P82995 | Heat shock protein HSP 90-alpha * | 46.33 | 16.37 | 3 | 0.88 | 0.92 | 0.71 | 0.76 | 0.82 | 0.1 | 84.76 | 5.01 | Hsp90aa1 | 299331 | P82995 | 122065208 | -0.745 | 0.224 | 4,5 |  | | | | | | | | | | | | | | | | | |
| B0BN63 | LOC681996 protein* | 15.37 | 16.86 | 4 | 0.82 | 0.96 | 0.76 | 0.81 | 0.84 | 0.08 | 38.08 | 5.53 | LOC681996 | 681996 | NP_001108506 | 169234816 | -0.48 | 0.105 |  |  | | | | | | | | | | | | | | | | | |
| F1LQH9 | Protein Bag2 (Fragment)* | 4.15 | 10.4 | 2 | 0.86 | 0.88 | 0.88 | 0.85 | 0.87 | 0.01 | 19.46 | 5.91 | Bag2 | 690038 | NP_001121667 | 189491875 | -0.61 | 0.103 |  |  | | | | | | | | | | | | | | | | | |
| P63018 | Heat shock cognate 71 kDa protein* | 273.3 | 50.77 | 32 | 0.91 | 0.89 | 0.84 | 0.87 | 0.88 | 0.03 | 70.83 | 5.52 | Hspa8 | 24468 | P63018 | 51702273 | -0.452 | 1.257 | 8 |  | | | | | | | | | | | | | | | | | |
| Q5BJQ0 | Chaperone activity of bc1 complex-like, mitochondrial* | 64.28 | 32.67 | 16 | 0.84 | 0.9 | 0.95 | 0.98 | 0.92 | 0.06 | 72.18 | 6.46 | Adck3 | 360887 | Q5BJQ0 | 81882496 | -0.247 | 0.305 |  |  | | | | | | | | | | | | | | | | | |
| D4ACB8 | Chaperonin subunit 8 (Theta) (Predicted), isoform CRA_a* | 7.67 | 5.11 | 2 | 1.02 | 0.88 |  |  | 0.95 |  | 59.55 | 5.54 | Cct8 | 288305 | NP_001099367 | 347800699 | -0.078 | 0.05 | 1 |  | | | | | | | | | | | | | | | | | |
| P18418 | Calreticulin* | 72.13 | 46.39 | 14 | 0.93 | 1 | 1.02 | 0.91 | 0.97 | 0.05 | 47.97 | 4.49 | Calr | 64202 | P18418 | 117505 | -1.099 | 0.709 | 5,7,8,1 |  | | | | | | | | | | | | | | | | | |
| P06761 | 78 kDa glucose-regulated protein* | 350 | 63.91 | 45 | 0.99 | 0.99 | 0.95 | 0.93 | 0.97 | 0.03 | 72.3 | 5.16 | Hspa5 | 25617 | P06761 | 121574 | -0.481 | 1.839 |  |  | | | | | | | | | | | | | | | | | |
| Q5XHZ0 | Heat shock protein 75 kDa, mitochondrial* | 14.38 | 8.07 | 3 |  |  | 1 | 1 | 1 |  | 80.41 | 7.03 | Trap1 | 287069 | Q5XHZ0 | 81910345 | -0.355 | 0.062 | 5,11,13,15,16,1 |  | | | | | | | | | | | | | | | | | |
| G3V836 | Clusterin* | 7.13 | 8.72 | 3 | 1.03 | 0.89 | 1.01 | 1.08 | 1 | 0.08 | 51.39 | 5.81 | Clu | 24854 | XP_006252156 | 564386466 | -0.54 | 0.058 |  |  | | | | | | | | | | | | | | | | | |
| Q66HD0 | Endoplasmin* | 49.67 | 17.29 | 12 | 1.03 | 1.01 | 1.04 | 1.1 | 1.05 | 0.04 | 92.71 | 4.81 | Hsp90b1 | 362862 | Q66HD0 | 205716800 | -0.732 | 0.237 | 8 |  | | | | | | | | | | | | | | | | | |
| Q6P502 | T-complex protein 1 subunit gamma* | 7.05 | 5.14 | 3 | 1.04 | 1.23 | 0.96 | 0.95 | 1.05 | 0.13 | 60.61 | 6.64 | Cct3 | 295230 | Q6P502 | 81911258 | -0.271 | 0.049 |  |  | | | | | | | | | | | | | | | | | |
| Q3MHS9 | Chaperonin containing Tcp1, subunit 6A (Zeta 1)* | 8.68 | 5.46 | 2 | 1.03 | 1.09 |  |  | 1.06 |  | 57.98 | 7.08 | Cct6a | 288620 | Q3MHS9 | 123786328 | -0.095 | 0.052 | 1 |  | | | | | | | | | | | | | | | | | |
| Q5U2R7 | LDLR chaperone MESD* | 9.11 | 15.18 | 3 | 1.08 | 1.18 |  |  | 1.13 |  | 25.2 | 5.66 | Mesdc2 | 308796 | Q5U2R7 | 81883487 | -0.824 | 0.119 |  |  | | | | | | | | | | | | | | | | | |
| G3V913 | Heat shock 27kDa protein 1* | 6.7 | 11.65 | 2 | 1.06 | 1.17 | 1.08 | 1.25 | 1.14 | 0.09 | 22.79 | 6.21 | Hspb1 | 24471 | EDM13341 | 149063018 | -0.453 | 0.088 |  |  | | | | | | | | | | | | | | | | | |
| P63039 | 60 kDa heat shock protein, mitochondrial* | 215.7 | 56.37 | 33 | 1.16 | 1.13 | 1.17 | 1.13 | 1.15 | 0.02 | 60.92 | 6.18 | Hspd1 | 63868 | P63039 | 51702230 | -0.085 | 1.264 |  |  | | | | | | | | | | | | | | | | | |
| P23928 | Alpha-crystallin B chain* | 144.1 | 50.86 | 15 | 1.23 | 1.23 | 1.21 | 1.21 | 1.22 | 0.01 | 20.08 | 7.33 | Cryab | 25420 | P23928 | 117388 | -0.527 | 2.441 |  |  | | | | | | | | | | | | | | | | | |
| P97541 | Heat shock protein beta-6* | 32.45 | 45.06 | 5 | 1.56 | 1.56 | 1.51 | 1.7 | 1.58 | 0.08 | 17.49 | 6.52 | Hspb6 | 192245 | P97541 | 6016271 | -0.144 | 0.629 |  |  | | | | | | | | | | | | | | | | | |
| D3ZZR9 | Peptidyl-prolyl cis-trans isomerase* | 13.24 | 25 | 3 | 1.87 | 1.82 | 2.03 | 1.94 | 1.94 | 0.09 | 15.38 | 8.88 | Fkbp2 | 293702 | XP_006230819 | 564332180 | -0.172 | 0.195 |  |  | | | | | | | | | | | | | | | | | |
| **cell signal** | | | | | | | | | | | | | | | | | | |  | |  |  |  |  |  |  |  |  |  |  |  |  |  |  |  |  |  |
| Q9Z1H9 | Protein kinase C delta-binding protein | 30.01 | 33.84 | 7 | 0.59 | 0.57 | 0.57 | 0.59 | 0.58 | 0.01 | 27.89 | 6.1 | Prkcdbp | 85332 | Q9Z1H9 | 81870080 | -0.542 | 0.43 | 8 |  | | | | | | | | | | | | | | | | | |
| D4A115 | Protein Col6a3 | 42.24 | 6.69 | 12 | 0.59 | 0.59 | 0.55 | 0.55 | 0.57 | 0.02 | 240.1 | 6.38 | Col6a3 | 367313 | XP_006227004 | 564312009 | -0.292 | 0.067 |  |  | | | | | | | | | | | | | | | | | |
| D3ZPR0 | Chromosome segregation 1-like (S. cerevisiae) (Predicted) | 4.32 | 1.96 | 2 | 0.57 | 0.8 |  |  | 0.69 |  | 110.1 | 5.77 | Cse1l | 362273 | NP_001102077 | 157820325 | -0.02 | 0.027 |  |  | | | | | | | | | | | | | | | | | |
| Q01129 | Decorin | 32.38 | 26.27 | 10 | 0.68 | 0.71 | 0.66 | 0.71 | 0.69 | 0.02 | 39.78 | 8.79 | Dcn | 29139 | Q01129 | 266763 | -0.22 | 0.427 |  |  | | | | | | | | | | | | | | | | | |
| P70580 | Membrane-associated progesterone receptor component 1 | 4.54 | 10.26 | 2 | 0.91 | 0.52 | 0.93 | 0.43 | 0.7 | 0.26 | 21.58 | 4.6 | Pgrmc1 | 291948 | P70580 | 6647578 | -0.598 | 0.185 |  |  | | | | | | | | | | | | | | | | | |
| F1M566 | Protein Hspg2 | 43.49 | 7.62 | 12 | 0.67 | 0.79 | 0.66 | 0.7 | 0.7 | 0.06 | 230.7 | 6.95 | Hspg2 | 117511 | EDL80840 | 149024343 | -0.278 | 0.061 |  |  | | | | | | | | | | | | | | | | | |
| D3ZUL3 | Protein Col6a1 | 15.52 | 5.66 | 5 | 0.6 | 0.73 | 0.67 | 0.84 | 0.67 | 0.1 | 108.7 | 5.36 | Col6a1 | 294337 | XP_001079629 | 293344916 | -0.536 | 0.055 |  |  | | | | | | | | | | | | | | | | | |
| F1M7X3 | Protein Cdh13 (Fragment) | 24.51 | 11.93 | 6 | 0.78 | 0.71 | 0.63 | 0.76 | 0.72 | 0.07 | 72.29 | 5.15 | Cdh13 | 192248 | NP_620244 | 20302073 | -0.218 | 0.111 |  |  | | | | | | | | | | | | | | | | | |
| F1LTJ5 | Protein Hspg2 | 29.43 | 6.55 | 8 | 0.74 | 0.71 | 0.69 | 0.75 | 0.72 | 0.03 | 216.3 | 5.67 | Hspg2 | 309368 | EDL93824 | 149039662 | -0.417 | 0.046 |  |  | | | | | | | | | | | | | | | | | |
| D3ZDQ9 | Protein Sgca | 9.17 | 10.59 | 3 | 0.82 | 0.71 | 0.84 | 0.53 | 0.72 | 0.14 | 43.13 | 6.62 | Sgca | 303468 | NP_001100509 | 157823585 | -0.127 | 0.07 |  |  | | | | | | | | | | | | | | | | | |
| F1LTF8 | Protein Lama4 | 5.6 | 1.87 | 3 | 0.77 | 0.82 | 0.77 | 0.65 | 0.75 | 0.08 | 195.5 | 6.52 | Lama4 | 309816 | XP_003753537 | 392338444 | -0.427 | 0.015 |  |  | | | | | | | | | | | | | | | | | |
| D4A1V7 | MOB1, Mps One Binder kinase activator-like 1A (Yeast) (Predicted) | 5.31 | 10.65 | 2 | 0.78 | 0.81 |  |  | 0.79 |  | 25.07 | 6.73 | Mob1b | 360920 | NP_001101827 | 157820997 | -0.338 | 0.08 | 1 |  | | | | | | | | | | | | | | | | | |
| D4AE17 | Protein B230120H23Rik | 6.97 | 6.84 | 2 |  |  | 0.88 | 0.71 | 0.79 | 0.12 | 51.28 | 5.3 | Zak | 311743 | XP_008760196 | 672046067 | -0.434 | 0.039 |  |  | | | | | | | | | | | | | | | | | |
| D3ZWA8 | Protein Appl1 (Fragment) | 4.03 | 2.03 | 2 | 0.74 | 0.89 |  |  | 0.81 |  | 77.45 | 5.55 | Appl1 | 290537 | XP_008769020 | 672029011 | -0.579 | 0.026 |  |  | | | | | | | | | | | | | | | | | |
| F1M614 | Protein Lama2 | 9.72 | 2.16 | 4 | 0.89 | 0.89 | 0.81 | 0.76 | 0.84 | 0.06 | 280.4 | 5.92 | Lama2 | 309368 | EDL93824 | 149039662 | -0.464 | 0.014 |  |  | | | | | | | | | | | | | | | | | |
| F1MAA7 | Protein Lamc1 | 39.21 | 8.34 | 10 | 0.88 | 0.85 | 0.82 | 0.86 | 0.85 | 0.03 | 177.3 | 5.22 | Lamc1 | 117036 | NP_446418 | 281371490 | -0.603 | 0.09 |  |  | | | | | | | | | | | | | | | | | |
| P36506 | Dual specificity mitogen-activated protein kinase kinase 2 | 13.64 | 14.75 | 5 | 0.84 | 0.69 | 0.93 | 0.95 | 0.85 | 0.12 | 44.25 | 7.09 | Map2k2 | 58960 | P36506 | 547916 | -0.33 | 0.181 |  |  | | | | | | | | | | | | | | | | | |
| P62161 | Calmodulin | 21.86 | 41.61 | 6 | 0.98 | 0.87 | 0.85 | 0.72 | 0.85 | 0.11 | 16.83 | 4.22 | Calm1 | 24242,24244,50663 | P62161 | 49037408 | -0.654 | 0.654 |  |  | | | | | | | | | | | | | | | | | |
| F8WFS5 | Growth hormone-regulated TBC protein 1 | 11.14 | 11.08 | 4 | 0.85 | 0.94 | 0.76 | 0.87 | 0.86 | 0.07 | 39.72 | 6.04 | Grtp1 | 290880 | NP_001013072 | 61556810 | -0.451 | 0.101 |  |  | | | | | | | | | | | | | | | | | |
| P0C089 | Phosphatidylglycerophosphatase and protein-tyrosine phosphatase 1 | 7.93 | 17.62 | 3 | 0.85 | 0.85 | 0.95 | 0.79 | 0.86 | 0.07 | 21.87 | 9.58 | Ptpmt1 | 29390 | P0C089 | 73621422 | -0.117 | 0.137 |  |  | | | | | | | | | | | | | | | | | |
| Q5XI34 | Protein Ppp2r1a | 4.32 | 3.57 | 2 | 1.04 | 0.71 |  |  | 0.87 |  | 65.28 | 5.11 | Ppp2r1a | 117281 | Q5XI34 | 81883690 | 0.075 | 0.031 |  |  | | | | | | | | | | | | | | | | | |
| Q5XIU9 | Membrane-associated progesterone receptor component 2 | 10.36 | 23.5 | 2 | 0.9 | 0.97 | 0.82 | 0.86 | 0.89 | 0.06 | 23.39 | 5.26 | Pgrmc2 | 361940 | Q5XIU9 | 62900631 | -0.459 | 0.171 |  |  | | | | | | | | | | | | | | | | | |
| F1LMK0 | A kinase (PRKA) anchor protein 1, isoform CRA_a | 6.36 | 6.62 | 3 | 0.87 | 0.92 | 0.95 | 0.82 | 0.89 | 0.06 | 57.62 | 4.96 | Akap1 | 114124 | EDM05642 | 149053825 | -0.502 | 0.052 |  |  | | | | | | | | | | | | | | | | | |
| P36860 | Ras-related protein Ral-B | 23.13 | 23.3 | 2 | 0.91 | 0.89 |  |  | 0.9 |  | 23.3 | 6.62 | Ralb | 116546 | P36860 | 548672 | -0.584 | 0.343 |  |  | | | | | | | | | | | | | | | | | |
| P63095 | Guanine nucleotide-binding protein G(s) subunit alpha isoforms short | 21.39 | 14.47 | 4 | 0.82 | 1 | 0.89 | 0.91 | 0.9 | 0.07 | 45.63 | 5.96 | Gnas | 24896 | P63095 | 52000956 | -0.597 | 0.219 | 8,1 |  | | | | | | | | | | | | | | | | | |
| G3V7W1 | Programmed cell death 6 (Predicted), isoform CRA_a | 12.84 | 26.18 | 4 | 0.85 | 0.92 | 0.88 | 0.99 | 0.91 | 0.06 | 21.89 | 5.64 | Pdcd6 | 308061 | EDL87681.1 | 157818653 | -0.418 | 0.228 |  |  | | | | | | | | | | | | | | | | | |
| P61983 | 14-3-3 protein gamma | 22.59 | 27.94 | 4 | 0.95 | 0.96 | 0.85 | 0.88 | 0.91 | 0.06 | 28.28 | 4.89 | Ywhag | 56010 | P61983 | 48428718 | -0.68 | 0.354 |  |  | | | | | | | | | | | | | | | | | |
| Q99J82 | Integrin-linked protein kinase | 12.17 | 10.18 | 4 | 0.96 | 0.87 |  |  | 0.91 |  | 51.34 | 8.07 | Ilk | 170922 | Q99J82 | 81916552 | -0.401 | 0.097 |  |  | | | | | | | | | | | | | | | | | |
| D3ZQN7 | Protein Lamb1 | 7.83 | 1.96 | 3 |  |  | 0.93 | 0.9 | 0.92 |  | 202.7 | 5.05 | Lamb1 | 298941 | XP_003750185 | 392348740 | -0.459 | 0.015 |  |  | | | | | | | | | | | | | | | | | |
| Q5XIG4 | OCIA domain-containing protein 1 | 10.67 | 19.03 | 3 | 0.85 | 0.96 | 0.87 | 1.01 | 0.92 | 0.08 | 27.64 | 7.42 | Ociad1 | 289590 | Q5XIG4 | 81883766 | -0.753 | 0.109 |  |  | | | | | | | | | | | | | | | | | |
| Q63638 | Striated muscle-specific serine/threonine-protein kinase | 14.36 | 2.7 | 6 | 0.94 | 0.91 | 0.96 | 0.89 | 0.92 | 0.03 | 354 | 8.28 | Speg | 363256 | Q63638 | 97537204 | -0.547 | 0.017 |  |  | | | | | | | | | | | | | | | | | |
| F1LWG8 | Protein Srl | 334 | 35.63 | 32 | 0.9 | 0.97 | 0.89 | 0.96 | 0.93 | 0.04 | 97.76 | 4.45 | Srl | 16018405 | AAI58712.1 | 165971273 | -0.689 | 1.197 |  |  | | | | | | | | | | | | | | | | | |
| P04897 | Guanine nucleotide-binding protein G(i) subunit alpha-2 | 32.24 | 21.13 | 4 | 0.92 | 0.96 | 0.87 | 0.98 | 0.93 | 0.05 | 40.47 | 5.45 | Gnai2 | 81664 | P04897 | 121025 | -0.372 | 0.222 | 8,1 |  | | | | | | | | | | | | | | | | | |
| Q01986 | Dual specificity mitogen-activated protein kinase kinase 1 | 4.21 | 7.12 | 2 | 0.9 | 0.98 |  |  | 0.94 |  | 43.44 | 6.62 | Map2k1 | 170851 | Q01986 | 266566 | -0.3 | 0.092 |  |  | | | | | | | | | | | | | | | | | |
| P54313 | Guanine nucleotide-binding protein G(I)/G(S)/G(T) subunit beta-2 | 39.2 | 28.53 | 6 | 0.9 | 0.9 | 1.1 | 0.9 | 0.95 | 0.1 | 37.31 | 6 | Gnb2 | 81667 | P54313 | 51338712 | -0.177 | 0.348 | 1 |  | | | | | | | | | | | | | | | | | |
| P14480 | Fibrinogen beta chain | 6.32 | 4.18 | 2 | 1.01 | 0.89 |  |  | 0.95 |  | 54.2 | 7.78 | Fgb | 24366 | P14480 | 124106312 | -0.739 | 0.037 | 14 |  | | | | | | | | | | | | | | | | | |
| G3V9N7 | Protein Pacsin3 | 23.99 | 19.48 | 7 | 1.09 | 0.82 | 0.92 | 0.98 | 0.95 | 0.11 | 48.7 | 6.1 | Pacsin3 | 311187 | XP_006234572.1 | 564341769 | -0.992 | 0.164 |  |  | | | | | | | | | | | | | | | | | |
| G3V715 | 5'-AMP-activated protein kinase catalytic subunit alpha-2 | 17.13 | 11.23 | 5 | 0.98 | 1.26 | 0.84 | 0.75 | 0.96 | 0.23 | 62.17 | 7.74 | Prkaa2 | 78975 | EDL97882 | 149044623 | -0.293 | 0.097 | 1 |  | | | | | | | | | | | | | | | | | |
| D4AE56 | Prostaglandin E synthase 2 (Predicted), isoform CRA_b | 99.35 | 56.77 | 18 | 0.98 | 0.96 | 0.9 | 1 | 0.96 | 0.04 | 43.42 | 9.25 | Ptges2 | 311865 | NP_001101302 | 157822395 | -0.295 | 0.737 | 1 |  | | | | | | | | | | | | | | | | | |
| Q9EQP5 | Prolargin | 4.79 | 5.04 | 2 | 0.89 | 1.05 |  |  | 0.97 |  | 43.15 | 9.45 | Prelp | 84400 | Q9EQP5 | 21542187 | -0.279 | 0.046 |  |  | | | | | | | | | | | | | | | | | |
| G3V6P8 | Guanine nucleotide-binding protein subunit gamma | 12.25 | 55.56 | 3 |  |  | 0.98 | 0.97 | 0.98 |  | 7.98 | 8.97 | Gng12 | 114120 | EDM08299.1 | 149056868 | -0.465 | 0.501 | 1 |  | | | | | | | | | | | | | | | | | |
| G3V9M6 | Fibrillin 1, isoform CRA_a | 4.29 | 0.66 | 2 | 0.91 | 0.97 | 0.97 | 1.07 | 0.98 | 0.06 | 311.7 | 4.93 | Fbn1 | 83727 | XP_006234997 | 564342789 | -0.421 | 0.006 | 1 |  | | | | | | | | | | | | | | | | | |
| F1LRZ1 | Ryanodine receptor 2 | 169.2 | 11.83 | 50 | 0.95 | 1.01 | 0.97 | 1.05 | 0.99 | 0.04 | 548.7 | 6.1 | Ryr2 | 689560 | NP_001177972 | 300795339 | -0.307 | 0.12 |  |  | | | | | | | | | | | | | | | | | |
| Q8CFN2 | Cell division control protein 42 homolog | 65.64 | 43.46 | 6 | 1.03 | 0.96 | 1.02 | 0.98 | 1 | 0.03 | 21.25 | 6.55 | Cdc42 | 64465 | Q8CFN2 | 122063303 | -0.157 | 1.036 |  |  | | | | | | | | | | | | | | | | | |
| D4A8F2 | Protein Rsu1 | 1.84 | 5.79 | 2 | 0.94 | 1.06 |  |  | 1 |  | 29.38 | 9.23 | Rsu1 | 680419 | EDL78715 | 149021108 | -0.476 | 0.068 |  |  | | | | | | | | | | | | | | | | | |
| D4A830 | Protein Ppa2 | 8.13 | 8.18 | 3 | 0.98 | 1.08 | 0.91 | 1.06 | 1.01 | 0.08 | 37.82 | 7.17 | Ppa2 | 310856 | NP_001129343 | 209529636 | -0.558 | 0.106 |  |  | | | | | | | | | | | | | | | | | |
| G3V8Q6 | Protein kinase, cAMP-dependent, regulatory, type 2, alpha, isoform CRA_a | 12.74 | 12.97 | 4 | 1.02 | 0.98 | 1 | 1.04 | 1.01 | 0.03 | 45.45 | 4.93 | Prkar2a | 29699 | EDL77146 | 149018505 | -0.499 | 0.044 |  |  | | | | | | | | | | | | | | | | | |
| P63322 | Ras-related protein Ral-A | 55.05 | 33.5 | 5 | 1.03 | 1.06 | 1.09 | 0.93 | 1.05 | 0.07 | 23.54 | 7.11 | Rala | 81757 | P63322 | 54038996 | -0.621 | 0.807 |  |  | | | | | | | | | | | | | | | | | |
| G3V6S3 | Calumenin | 10.93 | 16.83 | 4 | 1.14 | 0.96 | 1.09 | 0.98 | 1.04 | 0.08 | 37.04 | 4.67 | Calu | 64366 | NP_071980 | 158186676 | -1.011 | 0.108 | 1 |  | | | | | | | | | | | | | | | | | |
| Q2PS20 | Junctophilin-2 | 74.32 | 23.7 | 13 | 1.02 | 1.12 | 1.07 | 0.99 | 1.05 | 0.06 | 74.21 | 8.37 | Jph2 | 296345 | Q2PS20 | 119371891 | -0.791 | 0.418 |  |  | | | | | | | | | | | | | | | | | |
| F8WFI0 | Taste receptor type 1 member 2 | 7.64 | 6.58 | 4 | 1.07 | 1 | 1.14 | 1.02 | 1.06 | 0.06 | 61.76 | 8.05 | Tas1r2 | 641316 | AAI68153 | 195540087 | -0.157 | 0.065 |  |  | | | | | | | | | | | | | | | | | |
| Q62636 | Ras-related protein Rap-1b | 75.14 | 57.07 | 4 | 1.1 | 1.04 | 0.96 | 1.15 | 1.07 | 0.08 | 20.78 | 5.78 | Rap1b | 171337 | Q62636 | 51338715 | -0.375 | 1.395 | 10,15 |  | | | | | | | | | | | | | | | | | |
| Q6AYI1 | DEAD (Asp-Glu-Ala-Asp) box polypeptide 5 | 35.29 | 17.89 | 9 | 1.02 | 1.11 | 1.09 | 1.1 | 1.08 | 0.04 | 69.2 | 8.92 | Ddx5 | 287765 | Q6AYI1 | 81891357 | -0.637 | 0.188 | 13,1 |  | | | | | | | | | | | | | | | | | |
| Q9QY17-4 | Isoform 4 of Protein kinase C and casein kinase substrate in neurons 2 protein | 10.21 | 11.46 | 5 | 1.03 | 1.14 | 0.97 | 1.21 | 1.08 | 0.11 | 51.26 | 5.39 | Pacsin2 | 124461 | XP_006242112 | 564361124 | -1.037 | 0.117 | 8,1 |  | | | | | | | | | | | | | | | | | |
| G3V8V3 | Phosphorylase | 19.82 | 9.14 | 4 | 0.97 | 1.18 | 1.03 | 1.17 | 1.09 | 0.11 | 97.23 | 7.11 | Pygm | 24701 | NP_036770 | 158138498 | -0.394 | 0.093 | 1 |  | | | | | | | | | | | | | | | | | |
| F2Z3T8 | Guanine nucleotide-binding protein subunit gamma (Fragment) | 6.79 | 26.87 | 3 | 1.35 | 0.9 | 1.33 | 0.8 | 1.1 | 0.29 | 7.18 | 9.85 | LOC100360499 | 79218 | NP_077353 | 13242281 | -0.128 | 0.696 | 1 |  | | | | | | | | | | | | | | | | | |
| B2RZ37 | Receptor expression-enhancing protein 5 | 40.16 | 25.93 | 10 | 1.2 | 1.01 | 1.08 | 1.09 | 1.1 | 0.08 | 21.42 | 8.12 | Reep5 | 364838 | B2RZ37 | 259563717 | 0.118 | 0.887 |  |  | | | | | | | | | | | | | | | | | |
| P08050 | Gap junction alpha-1 protein | 46.02 | 18.32 | 8 | 1.09 | 0.97 | 1.18 | 1.14 | 1.1 | 0.09 | 43 | 8.76 | Gja1 | 24392 | P08050 | 117708 | -0.22 | 0.605 |  |  | | | | | | | | | | | | | | | | | |
| P20171 | GTPase Hras | 9.21 | 12.17 | 2 |  |  | 1.08 | 1.11 | 1.1 |  | 21.28 | 5.31 | Hras1 | 293621 | P20171 | 341941798 | -0.417 | 0.141 |  |  | | | | | | | | | | | | | | | | | |
| P52287 | Guanine nucleotide-binding protein G(I)/G(S)/G(T) subunit beta-3 | 46.36 | 31.47 | 7 | 1.07 | 1.17 | 1.03 | 1.15 | 1.1 | 0.06 | 37.16 | 5.83 | Gnb3 | 60449 | P52287 | 1730216 | -0.077 | 0.404 | 1 |  | | | | | | | | | | | | | | | | | |
| Q3KR94 | Protein Vtn | 11.34 | 10.46 | 3 | 1.15 | 1.15 | 1.08 | 1.08 | 1.15 | 0.04 | 54.69 | 6.05 | Vtn | 29169 | Q3KR94 | 123780627 | -0.758 | 0.055 |  |  | | | | | | | | | | | | | | | | | |
| Q6RUV5 | Ras-related C3 botulinum toxin substrate 1 | 30.79 | 36.98 | 7 | 1.16 | 1.18 | 1.02 | 1.15 | 1.13 | 0.07 | 21.44 | 8.5 | Rac1 | 363875 | Q6RUV5 | 51701705 | -0.101 | 0.793 |  |  | | | | | | | | | | | | | | | | | |
| Q66HA6 | ADP-ribosylation factor-like protein 8B | 23.98 | 35.48 | 4 | 1.18 | 1.13 | 1.19 | 1.07 | 1.14 | 0.06 | 21.53 | 8.43 | Arl8b | 500282 | Q66HA6 | 81890516 | -0.272 | 0.232 | 1 |  | | | | | | | | | | | | | | | | | |
| P61589 | Transforming protein RhoA | 73.41 | 48.7 | 3 | 1.25 | 0.97 | 1.36 | 0.98 | 1.14 | 0.19 | 21.77 | 6.1 | Rhoa | 117273 | P61589 | 47605935 | -0.366 | 1.194 |  |  | | | | | | | | | | | | | | | | | |
| P05545 | Serine protease inhibitor A3K | 9.8 | 8.65 | 2 | 1.02 | 1.02 | 1.19 | 1.4 | 1.16 | 0.18 | 46.53 | 5.44 | Serpina3k | 24794 | P05545 | 266407 | -0.158 | 0.043 |  |  | | | | | | | | | | | | | | | | | |
| P62836 | Ras-related protein Rap-1A | 81.26 | 53.26 | 3 | 1.07 | 1.04 | 1.39 | 1.15 | 1.16 | 0.16 | 20.97 | 6.67 | Rap1a | 295347 | P62836 | 51338596 | -0.375 | 1.335 | 15 |  | | | | | | | | | | | | | | | | | |
| Q5BJU0 | Protein Rras2 | 27.86 | 32.84 | 6 | 1.08 | 1.24 | 1.11 | 1.32 | 1.16 | 0.11 | 23.38 | 6.01 | Rras2 | 365355 | Q5BJU0 | 81882523 | -0.549 | 0.385 |  |  | | | | | | | | | | | | | | | | | |
| Q5U2V4 | Phospholipase B-like 1 | 10.91 | 6.36 | 3 | 1.16 | 1.32 | 1.16 | 1.14 | 1.2 | 0.08 | 62.99 | 8.6 | Plbd1 | 297694 | Q5U2V4 | 81883513 | -0.283 | 0.079 |  |  | | | | | | | | | | | | | | | | | |
| P63245 | Guanine nucleotide-binding protein subunit beta-2-like 1 | 19.74 | 30.6 | 8 | 1.17 | 1.19 | 1.16 | 1.27 | 1.2 | 0.05 | 35.05 | 7.69 | Gnb2l1 | 83427 | P63245 | 54037164 | -0.251 | 0.228 | 1 |  | | | | | | | | | | | | | | | | | |
| D3ZNV6 | Protein Elmod2 | 11.26 | 7.85 | 3 | 1.41 | 1.16 | 1.31 | 1.17 | 1.26 | 0.12 | 34.76 | 8.57 | Elmod2 | 688581 | NP_001102976 | 157824028 | -0.312 | 0.345 |  |  | | | | | | | | | | | | | | | | | |
| D4ABT0 | Guanine nucleotide-binding protein G(o) subunit alpha | 21.1 | 18.08 | 3 |  |  | 1.29 | 1.26 | 1.28 |  | 40 | 6.01 | Gnao1 | 50664 | XP_008770543 | 672084188 | -0.357 | 0.2 | 1 |  | | | | | | | | | | | | | | | | | |
| G3V7K9 | GTP-binding protein RAD | 143.9 | 42.35 | 13 | 1.29 | 1.29 | 1.26 | 1.29 | 1.29 | 0.01 | 33.26 | 9.26 | Rrad | 83521 | NP_445790 | 399498561 | -0.51 | 1.112 |  |  | | | | | | | | | | | | | | | | | |
| Q6AYC2 | Immunity-related GTPase family M protein | 17.77 | 9.49 | 3 | 1.31 | 1.39 | 1.3 | 1.37 | 1.35 | 0.04 | 46.31 | 8.12 | Irgm | 303090 | Q6AYC2 | 81884566 | -0.134 | 0.108 |  |  | | | | | | | | | | | | | | | | | |
| Q5BK32 | FAS-associated factor 2 | 59.67 | 39.31 | 12 | 1.5 | 1.25 | 1.45 | 1.23 | 1.36 | 0.14 | 41.05 | 5.96 | Faf2 | 291000 | Q5BK32 | 81882585 | -0.739 | 0.512 |  |  | | | | | | | | | | | | | | | | | |
| B1WC88 | UPF0729 protein C18orf32 homolog | 23.91 | 36.11 | 5 | 1.71 | 1.62 | 1.75 | 1.78 | 1.71 | 0.07 | 8.16 | 9.17 | RGD1562987 | 498886 | B1WC88 | 224487703 | -0.189 | 1.837 |  |  | | | | | | | | | | | | | | | | | |
| F1LUV9 | Neural cell adhesion molecule 1 (Fragment) | 44.01 | 10.38 | 7 |  |  | 1.72 | 1.73 | 1.73 |  | 92.25 | 4.91 | Ncam1 | 24586 | XP_006243056 | 564363541 | -0.424 | 0.141 |  |  | | | | | | | | | | | | | | | | | |
| F7F469 | Protein Irgm2 | 132.4 | 19.11 | 21 | 1.84 | 1.77 | 1.7 | 1.65 | 1.74 | 0.08 | 157.7 | 7.53 | Igtp | 303163 | AAP86276 | 32527749 | -0.348 | 0.292 |  |  | | | | | | | | | | | | | | | | | |
| F1LNY3 | Neural cell adhesion molecule 1 (Fragment) | 49.92 | 20.43 | 13 | 2.01 | 2.05 |  |  | 2.03 |  | 93.34 | 4.89 | Ncam1 | 24586 | NP_113709 | 13928706 | -0.44 | 0.182 |  |  | | | | | | | | | | | | | | | | | |
| D4A6C5 | Protein Arhgap1 | 152.5 | 54.44 | 20 | 2.31 | 2.29 | 2.32 | 2.38 | 2.32 | 0.04 | 50.59 | 6.54 | Arhgap1 | 311193 | NP_001101217 | 211065497 | -0.398 | 1.107 |  |  | | | | | | | | | | | | | | | | | |
| **lipid droplet** | | | | | | | | | | | | | | | | | | |  | |  |  |  |  |  |  |  |  |  |  |  |  |  |  |  |  |  |
| M0R7Z9 | Protein Plin5 | 325.5 | 66.32 | 34 | 0.61 | 0.6 | 0.55 | 0.57 | 0.58 | 0.03 | 51.77 | 5.83 | Plin5 | 501283 | M0R7Z9 | 657341140 | -0.268 | 1.912 | 1 |  | | | | | | | | | | | | | | | | | |
| M0R7S5 | Protein Plin4 | 703.5 | 83.2 | 29 | 0.79 | 0.69 | 0.8 | 0.74 | 0.75 | 0.05 | 135.4 | 9.13 | Plin4 | 363331 | XP_008765038 | 672064170 | -0.022 | 1.292 | 12,1 |  | | | | | | | | | | | | | | | | | |
| P43884 | Perilipin-1 | 87.93 | 41.01 | 14 | 0.81 | 0.81 | 0.81 | 0.85 | 0.82 | 0.02 | 55.58 | 6.8 | Plin1 | 25629 | P43884 | 1172433 | -0.378 | 0.45 | 12 |  | | | | | | | | | | | | | | | | | |
| M0RA08 | Protein Plin3 | 527.7 | 84.47 | 37 | 1.09 | 0.95 | 1.06 | 0.96 | 1.01 | 0.07 | 47.31 | 5.72 | Plin3 | 316130 | XP_001061015 | 109486616 | -0.315 | 3.403 | 15 |  | | | | | | | | | | | | | | | | | |
| Q5U2U5 | Adipose differentiation related protein | 1238 | 78.2 | 47 | 1.53 | 1.44 | 1.567 | 1.38 | 1.48 | 0.07 | 46.23 | 7.05 | Plin2 | 298199 | Q5U2U5 | 81883507 | -0.262 | 7.44 | 4,1 |  | | | | | | | | | | | | | | | | | |
| **endoplasmic reticulum** | | | | | | | | | | | | | | | | | | |  | |  |  |  |  |  |  |  |  |  |  |  |  |  |  |  |  |  |
| Q9QZA2 | Programmed cell death 6-interacting protein | 9.92 | 3.67 | 3 |  |  | 0.8 | 0.87 | 0.84 |  | 96.57 | 6.52 | Pdcd6ip | 501083 | Q9QZA2 | 205371813 | -0.448 | 0.031 | 1 |  | | | | | | | | | | | | | | | | | |
| Q9ES40 | PRA1 family protein 3 | 13.51 | 20.21 | 3 | 0.92 | 0.89 | 0.84 | 0.81 | 0.86 | 0.05 | 21.53 | 9.61 | Arl6ip5 | 66028 | Q9ES40 | 57012987 | 0.363 | 0.232 |  |  | | | | | | | | | | | | | | | | | |
| B0BNG0 | ER membrane protein complex subunit 2 | 9.34 | 16.84 | 3 | 0.75 | 0.93 | 0.9 | 0.91 | 0.87 | 0.08 | 34.85 | 6.81 | Emc2 | 362905 | B0BNG0 | 189036952 | -0.726 | 0.143 |  |  | | | | | | | | | | | | | | | | | |
| G3V8S6 | Histidine rich calcium binding protein, isoform CRA_b | 317.9 | 47.48 | 27 | 0.94 | 0.86 | 0.99 | 0.79 | 0.89 | 0.09 | 86.45 | 4.54 | Hrc | 292905 | EDM07376 | 149055945 | -1.767 | 1.122 |  |  | | | | | | | | | | | | | | | | | |
| Q4V7D1 | Signal sequence receptor, alpha | 10.6 | 8.01 | 2 |  |  | 0.83 | 0.97 | 0.9 |  | 32.19 | 4.44 | Ssr1 | 361233 | Q4V7D1 | 81907891 | -0.343 | 0.093 | 5,7,16 |  | | | | | | | | | | | | | | | | | |
| Q5FVQ4 | Malectin | 3.2 | 9.97 | 2 | 1 | 0.94 |  |  | 0.97 |  | 32.4 | 5.55 | Mlec | 304543 | Q5FVQ4 | 62900389 | -0.22 | 0.062 |  |  | | | | | | | | | | | | | | | | | |
| Q9JKZ6 | Protein Trdn | 10.18 | 17.07 | 5 | 1.02 | 0.95 | 0.97 | 1 | 0.98 | 0.03 | 32.11 | 8.88 | Trdn | 59299 | Q9JKZ6 | 81868705 | -1.012 | 0.28 |  |  | | | | | | | | | | | | | | | | | |
| G3V6T7 | Protein disulfide isomerase associated 4 | 5.17 | 5.13 | 3 | 1 | 0.99 |  |  | 0.99 |  | 72.7 | 5.15 | Pdia4 | 116598 | EDL88288 | 149033487 | -0.541 | 0.041 |  |  | | | | | | | | | | | | | | | | | |
| G3V9T7 | ATPase ASNA1 | 6.84 | 8.91 | 2 | 1.05 | 1.1 | 1 | 0.87 | 1.01 | 0.1 | 38.8 | 4.91 | Asna1 | 288919 | NP_001093975 | 213512072 | -0.116 | 0.052 |  |  | | | | | | | | | | | | | | | | | |
| B0BMW2 | 3-hydroxyacyl-CoA dehydrogenase type-2 | 37.8 | 46.36 | 7 | 1.07 | 1.15 | 1.01 | 1.01 | 1.06 | 0.07 | 27.23 | 8.59 | Hsd17b10 | 63864 | NP_113870.1 | 13994225 | 0.237 | 0.33 |  |  | | | | | | | | | | | | | | | | | |
| Q5HZY0 | UBX domain-containing protein 4 | 56.16 | 35.18 | 16 | 1.14 | 0.99 | 1.09 | 1.04 | 1.06 | 0.07 | 56.36 | 6.04 | Ubxn4 | 304766 | Q5HZY0 | 81882961 | -0.73 | 0.479 |  |  | | | | | | | | | | | | | | | | | |
| P52555 | Endoplasmic reticulum resident protein 29 | 18.38 | 29.62 | 7 | 1 | 1.03 | 1.06 | 1.17 | 1.06 | 0.07 | 28.56 | 6.62 | Erp29 | 117030 | P52555 | 2507015 | -0.266 | 0.28 |  |  | | | | | | | | | | | | | | | | | |
| D3ZUY0 | Protein Rdh14 | 8.28 | 8.98 | 3 | 1.13 | 1.08 |  |  | 1.11 |  | 36.17 | 8.09 | Rdh14 | 500629 | NP_001102746 | 157817189 | -0.012 | 0.111 |  |  | | | | | | | | | | | | | | | | | |
| Q07984 | Translocon-associated protein subunit delta | 6.08 | 12.14 | 2 | 1.18 | 1.15 |  |  | 1.17 |  | 18.97 | 5.78 | Ssr4 | 29435 | Q07984 | 1174452 | 0.136 | 0.105 | 5 |  | | | | | | | | | | | | | | | | | |
| A0JN29 | Limb and neural patterns | 14.9 | 13.3 | 5 | 1.41 | 1.32 | 1.52 | 1.36 | 1.37 | 0.09 | 42.14 | 9.25 | Lnp | 362151 | NP_001070897 | 116812561 | -0.364 | 0.142 |  |  | | | | | | | | | | | | | | | | | |
| P46462 | Transitional endoplasmic reticulum ATPase | 417.5 | 66.5 | 47 | 1.54 | 1.47 | 1.54 | 1.55 | 1.53 | 0.04 | 89.29 | 5.26 | Vcp | 116643 | P46462 | 1174637 | -0.351 | 1.4 |  |  | | | | | | | | | | | | | | | | | |
| **lysome** | | | | | | | | | | | | | | | | | | |  | |  |  |  |  |  |  |  |  |  |  |  |  |  |  |  |  |  |
| D3ZF11 | Hepatitis B virus x interacting protein (Predicted), isoform CRA_a | 11.41 | 19.23 | 2 | 0.95 | 0.87 |  |  | 0.91 |  | 16.38 | 5.74 | Lamtor5 | 295357 | NP_001099932 | 157823647 | -0.056 | 0.183 |  |  | | | | | | | | | | | | | | | | | |
| Q642E6 | Tripeptidyl peptidase I | 19.08 | 9.06 | 4 | 1.12 | 1.12 | 1.12 | 1.06 | 1.11 | 0.03 | 61.27 | 6.64 | Tpp1 | 83534 | Q642E6 | 81884107 | -0.156 | 0.131 | 7 |  | | | | | | | | | | | | | | | | | |
| P27615 | Lysosome membrane protein 2 | 4.75 | 5.44 | 3 | 1.04 | 1.13 | 1.15 | 1.2 | 1.13 | 0.07 | 54.06 | 5.01 | Scarb2 | 117106 | P27615 | 126291 | -0.009 | 0.074 | 1 |  | | | | | | | | | | | | | | | | | |
| **mitochondria** | | | | | | | | | | | | | | | | | | |  | |  |  |  |  |  |  |  |  |  |  |  |  |  |  |  |  |  |
| Q75Q41 | Mitochondrial import receptor subunit TOM22 homolog | 13.47 | 33.8 | 3 | 0.59 | 0.6 | 0.52 | 0.56 | 0.59 | 0.03 | 15.48 | 4.34 | Tomm22 | 300075 | Q75Q41 | 81864913 | -0.149 | 0.194 |  |  | | | | | | | | | | | | | | | | | |
| F1M8L6 | Protein Ndufa3 | 4.62 | 20.24 | 2 | 0.68 | 0.7 |  |  | 0.69 |  | 9.38 | 8.5 | Ndufa3 | 691001 | XP_002725549 | 564296738 | 0.013 | 0.213 |  |  | | | | | | | | | | | | | | | | | |
| P29410-2 | Isoform 2 of Adenylate kinase 2, mitochondrial | 10.23 | 15.09 | 3 |  |  | 0.67 | 0.86 | 0.77 |  | 25.51 | 6.8 | Ak2 | 24184 | P29410 | 266401 | -0.319 | 0.118 |  |  | | | | | | | | | | | | | | | | | |
| Q60587 | Trifunctional enzyme subunit beta, mitochondrial | 250.9 | 66.11 | 27 | 0.8 | 0.75 | 0.78 | 0.78 | 0.78 | 0.02 | 51.38 | 9.47 | Hadhb | 171155 | Q60587 | 2501192 | -0.073 | 1.713 |  |  | | | | | | | | | | | | | | | | | |
| D3ZBE2 | Protein Acacb | 5.19 | 1.63 | 3 |  |  | 0.76 | 0.81 | 0.78 |  | 275.8 | 6.2 | Acacb | 116719 | XP_008767535 | 672073277 | -0.205 | 0.206 |  |  | | | | | | | | | | | | | | | | | |
| D3ZVS2 | L-2-hydroxyglutarate dehydrogenase (Predicted) | 90.7 | 47.3 | 19 | 0.78 | 0.81 | 0.73 | 0.83 | 0.79 | 0.04 | 50.7 | 8.48 | L2hgdh | 314196 | NP_001101498 | 157820173 | -0.077 | 0.572 |  |  | | | | | | | | | | | | | | | | | |
| P52873 | Pyruvate carboxylase, mitochondrial | 6.72 | 2.29 | 2 | 0.8 | 0.79 |  |  | 0.8 |  | 129.7 | 6.81 | Pc | 25104 | P52873 | 146345499 | -0.165 | 0.015 | 12,1 |  | | | | | | | | | | | | | | | | | |
| Q9WV97 | Mitochondrial import inner membrane translocase subunit Tim9 | 12.82 | 34.83 | 2 | 0.89 | 0.72 |  |  | 0.81 |  | 10.37 | 7.21 | Timm9 | 171139 | Q9WV97 | 90110082 | -0.582 | 0.289 |  |  | | | | | | | | | | | | | | | | | |
| Q64428 | Trifunctional enzyme subunit alpha, mitochondrial | 482.5 | 65.27 | 54 | 0.84 | 0.79 | 0.84 | 0.8 | 0.82 | 0.03 | 82.61 | 9.06 | Hadha | 170670 | Q64428 | 172045972 | -0.081 | 1.913 |  |  | | | | | | | | | | | | | | | | | |
| F7FKI5 | Pyruvate dehydrogenase E1 component subunit alpha, somatic form, mitochondrial | 141.6 | 60 | 2 | 0.83 | 0.83 |  |  | 0.83 |  | 43.22 | 8.19 | Pdha1 | 29554 | NP_001004072 | 124430510 | -0.295 | 1.643 | 1 |  | | | | | | | | | | | | | | | | | |
| Q66HF3 | Electron transfer flavoprotein-ubiquinone oxidoreductase, mitochondrial | 212 | 55.19 | 34 | 0.85 | 0.82 | 0.86 | 0.81 | 0.84 | 0.02 | 68.12 | 7.56 | Etfdh | 295143 | Q66HF3 | 81884210 | -0.31 | 1.086 | 6,2 |  | | | | | | | | | | | | | | | | | |
| Q7TP78 | Aa2-258 | 103.7 | 42.86 | 12 | 0.85 | 0.86 | 0.81 | 0.84 | 0.84 | 0.02 | 21.95 | 8.1 | Ndufa8 | 296658 | Q7TP78 | 81865328 | -0.581 | 1.64 |  |  | | | | | | | | | | | | | | | | | |
| P31399 | ATP synthase subunit d, mitochondrial | 157.4 | 88.2 | 14 | 0.86 | 0.81 | 0.89 | 0.87 | 0.86 | 0.04 | 18.75 | 6.6 | Atp5h | 641434 | P31399 | 1352051 | -0.702 | 3.626 | 1 |  | | | | | | | | | | | | | | | | | |
| P12075 | Cytochrome c oxidase subunit 5B, mitochondrial | 83.68 | 42.64 | 7 | 0.88 | 0.82 | 0.89 | 0.86 | 0.86 | 0.03 | 13.91 | 7.78 | Cox5b | 94194 | P12075 | 1352167 | -0.281 | 2.373 | 5,16,17 |  | | | | | | | | | | | | | | | | | |
| Q68FT1 | Ubiquinone biosynthesis protein COQ9, mitochondrial | 83.45 | 36.22 | 10 | 0.89 | 0.87 | 0.86 | 0.82 | 0.88 | 0.03 | 35.12 | 5.77 | Coq9 | 498909 | Q68FT1 | 90111992 | -0.454 | 0.769 |  |  | | | | | | | | | | | | | | | | | |
| G3V640 | Mitochondrial import inner membrane translocase subunit TIM44 | 30.21 | 19.87 | 9 | 0.87 | 0.9 | 0.83 | 0.87 | 0.87 | 0.03 | 51.03 | 8.31 | Timm44 | 29635 | EDL74999 | 149015618 | -0.502 | 0.274 |  |  | | | | | | | | | | | | | | | | | |
| D3ZEH6 | Protein Nudt8 | 6.55 | 12.38 | 2 | 0.87 | 0.89 |  |  | 0.88 |  | 23.08 | 6.93 | Nudt8 | 361692 | XP_341976 | 293356343 | -0.05 | 0.087 |  |  | | | | | | | | | | | | | | | | | |
| P04166 | Cytochrome b5 type B | 6.39 | 28.08 | 2 | 0.78 | 0.99 |  |  | 0.88 |  | 16.25 | 5.06 | Cyb5b | 80773 | P04166 | 12643974 | -0.626 | 0.123 | 4,7,10,13,15,1 |  | | | | | | | | | | | | | | | | | |
| Q3KRE0 | ATPase family AAA domain-containing protein 3 | 7.09 | 4.06 | 2 |  |  | 0.8 | 0.96 | 0.88 |  | 66.72 | 9.29 | Atad3 | 298682 | Q3KRE0 | 123783007 | -0.66 | 0.03 |  |  | | | | | | | | | | | | | | | | | |
| D3ZG43 | NADH dehydrogenase (Ubiquinone) Fe-S protein 3 (Predicted), isoform CRA_c | 107.5 | 48.11 | 16 | 0.86 | 0.88 | 0.9 | 0.9 | 0.89 | 0.02 | 30.21 | 7.53 | Ndufs3 | 295923 | NP_001099959 | 157817227 | -0.325 | 1.258 | 2,1 |  | | | | | | | | | | | | | | | | | |
| D3ZD09 | Cytochrome c oxidase subunit 6B1 | 100.6 | 70.93 | 9 | 0.85 | 0.9 | 0.94 | 0.87 | 0.89 | 0.04 | 10.06 | 8.72 | Cox6b1 | 688869 | NP_001138745.1 | 223718723 | -0.764 | 3.477 | 5,16,1 |  | | | | | | | | | | | | | | | | | |
| B5DFN3 | Mitochondrial nucleoid factor 1 | 6.78 | 33.09 | 4 | 0.87 | 0.91 |  |  | 0.89 |  | 16.32 | 9.23 | Mnf1 | 361805 | B5DFN3 | 384950688 | -1.037 | 0.245 |  |  | | | | | | | | | | | | | | | | | |
| B5DEL8 | NADH dehydrogenase (Ubiquinone) Fe-S protein 5 | 66.57 | 78.3 | 13 | 0.87 | 0.88 | 0.91 | 0.9 | 0.89 | 0.02 | 12.69 | 8.92 | Ndufs5 | 100363268,362588 | NP_001025223 | 72086149 | -0.9 | 2.206 | 1 |  | | | | | | | | | | | | | | | | | |
| B2RYS2 | Cytochrome b-c1 complex subunit 7 | 160.6 | 78.38 | 17 | 0.92 | 0.87 | 0.92 | 0.85 | 0.89 | 0.04 | 13.55 | 9.31 | Uqcrb | 362897 | NP_001121025 | 189011657 | -1.05 | 4.797 | 4,7,8,10,13,15,1 |  | | | | | | | | | | | | | | | | | |
| P08461 | Dihydrolipoyllysine-residue acetyltransferase component of pyruvate dehydrogenase complex, mitochondrial | 126.3 | 38.29 | 20 | 0.91 | 0.91 | 0.87 | 0.87 | 0.89 | 0.02 | 67.12 | 8.53 | Dlat | 81654 | P08461 | 119364626 | -0.017 | 0.566 |  |  | | | | | | | | | | | | | | | | | |
| D3ZS58 | NADH dehydrogenase [ubiquinone] 1 alpha subcomplex subunit 2 | 38.29 | 62.89 | 9 | 0.86 | 0.99 | 0.83 | 0.88 | 0.89 | 0.07 | 10.84 | 9.72 | Ndufa2 | 291660 | NP_001099623 | 157817861 | -0.301 | 1.938 | 1 |  | | | | | | | | | | | | | | | | | |
| G3V7Y3 | ATP synthase subunit delta, mitochondrial | 36.87 | 41.07 | 4 | 0.98 | 0.84 | 0.9 | 0.85 | 0.89 | 0.06 | 17.55 | 5.24 | Atp5d | 245965 | EDL89324 | 149034587 | 0.211 | 0.57 | 1 |  | | | | | | | | | | | | | | | | | |
| D4A3V2 | NADH dehydrogenase [ubiquinone] 1 alpha subcomplex subunit 6 | 67.05 | 52.31 | 7 | 0.91 | 0.84 | 0.98 | 0.86 | 0.9 | 0.06 | 15.21 | 10.15 | Ndufa6 | 315167 | NP_001123977 | 194473636 | -0.54 | 1.446 | 1 |  | | | | | | | | | | | | | | | | | |
| P80431 | Cytochrome c oxidase subunit 7B, mitochondrial | 10.38 | 10 | 2 | 1.13 | 0.64 | 0.99 | 0.85 | 0.88 | 0.21 | 8.99 | 10.11 | Cox7b | 303393 | P80431 | 118572633 | -0.337 | 0.89 | 1 |  | | | | | | | | | | | | | | | | | |
| P29418 | ATP synthase subunit epsilon, mitochondrial | 9.96 | 54.9 | 4 | 0.85 | 0.99 | 0.76 | 1.01 | 0.9 | 0.12 | 5.76 | 10.01 | Atp5e | 245958 | P29418 | 2851397 | -0.367 | 1.041 | 5 |  | | | | | | | | | | | | | | | | | |
| P10817 | Cytochrome c oxidase subunit 6A2, mitochondrial (Fragment) | 31.38 | 43.62 | 2 | 0.89 | 0.86 | 0.98 | 0.88 | 0.9 | 0.06 | 10.48 | 9.07 | Cox6a2 | 25278 | P10817 | 1352170 | -0.354 | 0.954 | 1 |  | | | | | | | | | | | | | | | | | |
| D3ZAF6 | ATP synthase subunit f, mitochondrial | 25.35 | 34.09 | 5 | 0.91 | 0.85 | 0.94 | 0.93 | 0.91 | 0.04 | 10.45 | 10.04 | Atp5j2 | 690441 | D3ZAF6 | 385178613 | -0.348 | 0.957 | 5,11,12,16,1 |  | | | | | | | | | | | | | | | | | |
| P35171 | Cytochrome c oxidase subunit 7A2, mitochondrial | 9.75 | 27.71 | 2 | 0.94 | 0.95 | 0.86 | 0.88 | 0.91 | 0.04 | 9.35 | 10.27 | Cox7a2 | 29507,688386 | P35171 | 461803 | -0.258 | 0.428 | 5,16,1 |  | | | | | | | | | | | | | | | | | |
| B0BNE6 | NADH dehydrogenase (Ubiquinone) Fe-S protein 8 (Predicted), isoform CRA_a | 72.16 | 40.09 | 11 | 0.86 | 0.94 | 0.91 | 0.92 | 0.91 | 0.03 | 23.95 | 6.21 | Ndufs8 | 293652 | NP_001099792 | 157821497 | -0.374 | 0.96 | 1 |  | | | | | | | | | | | | | | | | | |
| D3ZLT1 | NADH dehydrogenase (Ubiquinone) 1 beta subcomplex, 7 (Predicted) | 15.18 | 35.04 | 4 | 0.88 | 0.95 | 0.93 | 0.87 | 0.91 | 0.04 | 16.56 | 8.46 | Ndufb7 | 361385 | NP_001101912 | 157823197 | -1.102 | 0.423 | 1 |  | | | | | | | | | | | | | | | | | |
| M0R6J6 | Creatine kinase S-type, mitochondrial | 168.2 | 33.5 | 6 | 0.99 | 0.81 | 0.98 | 0.87 | 0.9 | 0.09 | 46.42 | 9.5 | Ckmt2 | 688698 | NP_001121124 | 189083744 | -0.296 | 1.12 | 1 |  | | | | | | | | | | | | | | | | | |
| P19234 | NADH dehydrogenase [ubiquinone] flavoprotein 2, mitochondrial | 90.37 | 54.44 | 11 | 0.88 | 0.92 | 0.92 | 0.92 | 0.91 | 0.02 | 27.36 | 6.68 | Ndufv2 | 81728 | P19234 | 83305118 | -0.316 | 1.06 | 1 |  | | | | | | | | | | | | | | | | | |
| Q63362 | NADH dehydrogenase [ubiquinone] 1 alpha subcomplex subunit 5 | 63.94 | 61.21 | 9 | 0.95 | 0.8 | 1.03 | 0.86 | 0.91 | 0.1 | 13.4 | 7.43 | Ndufa5 | 25488 | Q63362 | 2499317 | -0.349 | 2.387 | 1 |  | | | | | | | | | | | | | | | | | |
| G3V6D3 | ATP synthase subunit beta | 837.9 | 75.8 | 31 | 0.91 | 0.92 | 0.92 | 0.92 | 0.92 | 0 | 56.31 | 5.27 | Atp5b | 171374 | EDL84889 | 149029718 | 0.033 | 4.102 | 5 |  | | | | | | | | | | | | | | | | | |
| G3V734 | 2,4-dienoyl CoA reductase 1, mitochondrial, isoform CRA_a | 35.36 | 34.33 | 9 | 0.93 | 0.96 | 0.87 | 0.9 | 0.92 | 0.04 | 36.11 | 8.94 | Decr1 | 117543 | EDL98478.**1** | 149045478 | -0.005 | 0.388 | 1 |  | | | | | | | | | | | | | | | | | |
| G3V8F5 | Mitochondrial import receptor subunit TOM40 homolog | 18.88 | 11.08 | 4 | 0.9 | 0.93 | 0.92 | 0.92 | 0.92 | 0.01 | 37.9 | 7.97 | Tomm40 | 308416 | XP_006228482 | 564326563 | -0.117 | 0.237 |  |  | | | | | | | | | | | | | | | | | |
| D4A7L4 | NADH dehydrogenase (Ubiquinone) 1 beta subcomplex, 11 (Predicted) | 17.06 | 21.19 | 2 | 0.91 | 0.94 | 0.95 | 0.88 | 0.92 | 0.03 | 17.62 | 5.24 | Ndufb11 | 299310 | NP_001100226 | 157822851 | -0.436 | 0.227 | 1 |  | | | | | | | | | | | | | | | | | |
| P15999 | ATP synthase subunit alpha, mitochondrial | 667.9 | 76.49 | 58 | 0.95 | 0.9 | 0.93 | 0.9 | 0.92 | 0.02 | 59.72 | 9.19 | Atp5a1 | 65262 | P15999 | 83300587 | -0.09 | 3.617 |  |  | | | | | | | | | | | | | | | | | |
| Q9WVK7 | Hydroxyacyl-coenzyme A dehydrogenase, mitochondrial | 111 | 55.41 | 16 | 0.97 | 0.84 | 0.96 | 0.92 | 0.92 | 0.06 | 34.43 | 8.76 | Hadh | 113965 | Q9WVK7 | 7387725 | -0.142 | 1.046 | 1 |  | | | | | | | | | | | | | | | | | |
| Q5PQZ9 | NADH dehydrogenase [ubiquinone] 1 subunit C2 | 35.8 | 31.67 | 6 | 0.94 | 0.9 | 0.98 | 0.87 | 0.92 | 0.05 | 14.35 | 9.39 | Ndufc2 | 293130 | Q5PQZ9 | 81883284 | -0.473 | 1.324 |  |  | | | | | | | | | | | | | | | | | |
| F1MA54 | [Pyruvate dehydrogenase [lipoamide]] kinase isozyme 1, mitochondrial | 21.75 | 15.21 | 4 | 0.86 | 0.91 | 0.98 | 0.96 | 0.93 | 0.05 | 49.07 | 8 | Pdk1 | 116551 | NP_446278 | 59709473 | -0.324 | 0.122 | 1 |  | | | | | | | | | | | | | | | | | |
| P20788 | Cytochrome b-c1 complex subunit Rieske, mitochondrial | 141.8 | 57.66 | 12 | 0.96 | 0.9 | 0.94 | 0.9 | 0.93 | 0.03 | 29.43 | 8.87 | Uqcrfs1 | 291103 | P20788 | 52001457 | -0.108 | 1.393 |  |  | | | | | | | | | | | | | | | | | |
| P49432 | Pyruvate dehydrogenase E1 component subunit beta, mitochondrial | 143.2 | 55.99 | 17 | 0.96 | 0.9 | 0.96 | 0.9 | 0.93 | 0.03 | 38.96 | 6.65 | Pdhb | 289950 | P49432 | 122065728 | 0.045 | 1.078 | 1 |  | | | | | | | | | | | | | | | | | |
| Q66HF1 | NADH-ubiquinone oxidoreductase 75 kDa subunit, mitochondrial | 352.2 | 64.51 | 44 | 0.94 | 0.93 | 0.9 | 0.94 | 0.93 | 0.02 | 79.36 | 5.9 | Ndufs1 | 301458 | Q66HF1 | 81884209 | -0.101 | 1.424 | 5,1 |  | | | | | | | | | | | | | | | | | |
| D3ZYX8 | Cytochrome c oxidase subunit VIIa polypeptide 2 like (Predicted), isoform CRA_e | 20.06 | 36.67 | 5 | 0.96 | 0.95 | 0.92 | 0.89 | 0.95 | 0.03 | 13.26 | 9.74 | Cox7a2l | 298762 | NP_001100174 | 157821821 | -0.12 | 0.452 | 5,1 |  | | | | | | | | | | | | | | | | | |
| D3ZN43 | NADH dehydrogenase (ubiquinone) complex I, assembly factor 6 | 6.96 | 7.21 | 2 | 0.93 | 1 | 0.9 | 0.88 | 0.93 | 0.05 | 38.49 | 9.63 | Ndufaf6 | 297821 | D3ZN43 | 395406791 | -0.422 | 0.052 |  |  | | | | | | | | | | | | | | | | | |
| Q5RJN0 | NADH dehydrogenase (Ubiquinone) Fe-S protein 7 | 73.6 | 35.32 | 6 | 0.91 | 0.92 | 0.9 | 1 | 0.93 | 0.05 | 23.93 | 9.99 | Ndufs7 | 362837 | Q5RJN0 | 81883325 | -0.042 | 0.919 | 2,1 |  | | | | | | | | | | | | | | | | | |
| P11240 | Cytochrome c oxidase subunit 5A, mitochondrial | 141.6 | 63.01 | 11 | 0.94 | 0.9 | 0.99 | 0.89 | 0.93 | 0.04 | 16.12 | 6.54 | Cox5a | 252934 | P11240 | 117100 | -0.142 | 2.854 | 5,1 |  | | | | | | | | | | | | | | | | | |
| O35796 | Complement component 1 Q subcomponent-binding protein, mitochondrial | 12.37 | 16.85 | 3 | 0.9 | 1.06 | 0.78 | 0.99 | 0.93 | 0.12 | 30.98 | 4.87 | C1qbp | 29681 | O35796 | 122065146 | -0.441 | 0.097 |  |  | | | | | | | | | | | | | | | | | |
| Q5XIH7 | Prohibitin-2 | 70.7 | 54.85 | 15 | 0.88 | 0.97 | 0.89 | 1 | 0.93 | 0.06 | 33.29 | 9.83 | Phb2 | 114766 | Q5XIH7 | 76363296 | -0.266 | 0.721 | 6,7,12,1 |  | | | | | | | | | | | | | | | | | |
| P10860 | Glutamate dehydrogenase 1, mitochondrial | 88.79 | 38.17 | 16 | 0.92 | 0.92 | 0.95 | 0.95 | 0.93 | 0.02 | 61.38 | 8 | Glud1 | 24399 | P10860 | 92090591 | -0.306 | 0.505 | 1 |  | | | | | | | | | | | | | | | | | |
| Q63159 | Hexaprenyldihydroxybenzoate methyltransferase, mitochondrial | 13.96 | 13.04 | 4 | 0.87 | 0.89 | 1.04 | 0.95 | 0.88 | 0.08 | 38.68 | 7.97 | Coq3 | 29309 | Q63159 | 57015268 | -0.247 | 0.129 |  |  | | | | | | | | | | | | | | | | | |
| Q4G064 | 2-methoxy-6-polyprenyl-1,4-benzoquinol methylase, mitochondrial | 25.91 | 22.63 | 7 | 0.89 | 0.99 | 0.96 | 0.91 | 0.93 | 0.05 | 37.28 | 8.81 | Coq5 | 304542 | Q4G064 | 90111989 | -0.339 | 0.402 |  |  | | | | | | | | | | | | | | | | | |
| P11951 | Cytochrome c oxidase subunit 6C-2 | 27.27 | 72.37 | 7 | 1 | 0.91 | 1 | 0.84 | 0.94 | 0.08 | 8.45 | 10.07 | Cox6c2 | 54322 | P11951 | 117119 | -0.108 | 2.604 | 5,1 |  | | | | | | | | | | | | | | | | | |
| P29419 | ATP synthase subunit e, mitochondrial | 72.12 | 88.73 | 12 | 0.99 | 0.97 | 0.93 | 0.87 | 0.94 | 0.05 | 8.25 | 9.35 | Atp5i | 140608 | P29419 | 461587 | -0.494 | 4.606 |  |  | | | | | | | | | | | | | | | | | |
| Q920L2 | Succinate dehydrogenase [ubiquinone] flavoprotein subunit, mitochondrial | 262 | 63.11 | 31 | 0.95 | 0.9 | 0.97 | 0.95 | 0.94 | 0.03 | 71.57 | 7.17 | Sdha | 157074 | Q920L2 | 52782765 | -0.254 | 1.23 | 1 |  | | | | | | | | | | | | | | | | | |
| F1LPG5 | Protein LOC688963 | 51.43 | 54.26 | 9 | 0.97 | 0.9 | 1 | 0.92 | 0.95 | 0.05 | 15.05 | 9.82 | LOC688963 | 288088 | NP_001032415 | 82617686 | -0.676 | 1.661 |  |  | | | | | | | | | | | | | | | | | |
| Q5XIF3 | NADH dehydrogenase [ubiquinone] iron-sulfur protein 4, mitochondrial | 83.96 | 41.71 | 10 | 0.99 | 0.97 | 0.86 | 0.96 | 0.95 | 0.06 | 19.73 | 10.14 | Ndufs4 | 499529 | Q5XIF3 | 81889861 | -0.545 | 1.419 | 1 |  | | | | | | | | | | | | | | | | | |
| P10818 | Cytochrome c oxidase subunit 6A1, mitochondrial | 51.56 | 42.34 | 3 | 1.01 | 0.92 | 1 | 0.86 | 0.96 | 0.07 | 12.29 | 9.32 | Cox6a1 | 25282 | P10818 | 1352174 | -0.338 | 1.302 | 5,1 |  | | | | | | | | | | | | | | | | | |
| D3ZCZ9 | Protein LOC100912599 | 116.1 | 65.52 | 8 | 0.99 | 0.94 | 0.96 | 0.9 | 0.95 | 0.04 | 13.03 | 9.22 | LOC100912599 | 100912599,679739 | XP_001053017 | 109504524 | -0.522 | 2.532 |  |  | | | | | | | | | | | | | | | | | |
| Q5BK63 | NADH dehydrogenase [ubiquinone] 1 alpha subcomplex subunit 9, mitochondrial | 172.6 | 67.11 | 26 | 1.02 | 0.89 | 0.98 | 0.91 | 0.95 | 0.06 | 42.53 | 9.82 | Ndufa9 | 362440 | Q5BK63 | 317373396 | -0.091 | 1.599 | 1 |  | | | | | | | | | | | | | | | | | |
| Q641Y2 | NADH dehydrogenase [ubiquinone] iron-sulfur protein 2, mitochondrial | 142.4 | 54.86 | 18 | 0.92 | 1 | 0.92 | 0.96 | 0.95 | 0.04 | 52.53 | 6.99 | Ndufs2 | 289218 | Q641Y2 | 81890431 | -0.27 | 0.876 |  |  | | | | | | | | | | | | | | | | | |
| Q561S0 | NADH dehydrogenase [ubiquinone] 1 alpha subcomplex subunit 10, mitochondrial | 178.2 | 56.62 | 21 | 0.93 | 0.95 | 0.97 | 0.96 | 0.95 | 0.02 | 40.47 | 7.8 | Ndufa10 | 316632,678759 | Q561S0 | 81882328 | -0.436 | 1.483 | 1 |  | | | | | | | | | | | | | | | | | |
| Q5XII9 | Mitochondrial fission regulator 1-like | 10.72 | 10.03 | 2 |  |  | 0.94 | 0.97 | 0.95 |  | 31.71 | 6.1 | Mtfr1l | 298549 | Q5XII9 | 81883784 | -0.289 | 0.095 |  |  | | | | | | | | | | | | | | | | | |
| P09605 | Creatine kinase S-type, mitochondrial | 231.5 | 60.38 | 12 | 1.04 | 0.93 | 0.96 | 0.87 | 0.95 | 0.07 | 47.36 | 8.51 | Ckmt2 | 688698 | P09605 | 125313 | -0.448 | 1.436 | 1 |  | | | | | | | | | | | | | | | | | |
| Q62651 | Delta(3,5)-Delta(2,4)-dienoyl-CoA isomerase, mitochondrial | 45.13 | 33.64 | 11 | 1 | 0.96 | 0.91 | 0.97 | 0.98 | 0.04 | 36.15 | 7.99 | Ech1 | 64526 | Q62651 | 6015047 | -0.097 | 0.443 |  |  | | | | | | | | | | | | | | | | | |
| P67779 | Prohibitin | 104.3 | 78.31 | 15 | 0.99 | 0.97 | 0.94 | 0.94 | 0.96 | 0.03 | 29.8 | 5.76 | Phb | 25344 | P67779 | 54038835 | 0.009 | 1.107 | 6,7,12,1 |  | | | | | | | | | | | | | | | | | |
| F1LXA0 | NADH dehydrogenase (Ubiquinone) 1 alpha subcomplex, 12 (Predicted), isoform CRA_b | 56.15 | 69.66 | 9 | 0.95 | 0.99 | 1.01 | 0.91 | 0.96 | 0.05 | 17.17 | 9.7 | LOC100910710 | 299739 | NP_001100251 | 164565371 | -0.812 | 1.34 | 1 |  | | | | | | | | | | | | | | | | | |
| P32551 | Cytochrome b-c1 complex subunit 2, mitochondrial | 223.1 | 50.66 | 29 | 0.96 | 0.99 | 0.97 | 0.95 | 0.97 | 0.02 | 48.37 | 9.14 | Uqcrc2 | 293448 | P32551 | 122066611 | -0.067 | 1.571 |  |  | | | | | | | | | | | | | | | | | |
| G3V9S0 | Cytochrome b5 reductase 1 | 6.05 | 5.9 | 2 | 0.93 | 1.01 |  |  | 0.97 |  | 34.21 | 8.84 | Cyb5r1 | 304805 | EDM09729 | 149058572 | -0.134 | 0.088 | 4,7,10,13,15,1 |  | | | | | | | | | | | | | | | | | |
| D4A471 | Protein 2310061C15Rik | 2.03 | 25.32 | 2 | 1.01 | 0.93 |  |  | 0.97 |  | 9.4 | 7.88 | Cmc2 | 100363376 |  |  | -1.153 | 0.213 |  |  | | | | | | | | | | | | | | | | | |
| Q3KR86 | Mitochondrial inner membrane protein (Fragment) | 301.6 | 73.4 | 46 | 0.98 | 0.99 | 0.96 | 0.96 | 0.97 | 0.01 | 67.14 | 5.8 | Immt | 312444 | Q3KR86 | 123780621 | -0.473 | 1.594 |  |  | | | | | | | | | | | | | | | | | |
| D4A4P3 | Protein LOC100361144 | 14.24 | 26.26 | 3 | 0.91 | 1.04 |  |  | 0.97 |  | 11.26 | 9.04 | LOC100361144 | 301427 | NP_001100382 | 157824071 | -0.578 | 0.444 |  |  | | | | | | | | | | | | | | | | | |
| Q5M9I5 | Cytochrome b-c1 complex subunit 6, mitochondrial | 59.3 | 74.16 | 7 | 0.97 | 0.94 | 1 | 0.99 | 0.97 | 0.02 | 10.42 | 4.97 | Uqcrh | 366448 | Q5M9I5 | 62511137 | -1.019 | 1.632 |  |  | | | | | | | | | | | | | | | | | |
| F1M8L5 | Propionyl-CoA carboxylase alpha chain, mitochondrial | 28.13 | 17.73 | 8 | 1.01 | 0.97 | 0.95 | 0.97 | 0.97 | 0.03 | 69.19 | 7.21 | Pcca | 687008 | XP_008769195 | 672079171 | -0.207 | 0.173 |  |  | | | | | | | | | | | | | | | | | |
| Q5RKI8 | ATP-binding cassette sub-family B member 8, mitochondrial | 10.48 | 4.06 | 3 | 1 | 0.95 |  |  | 0.98 |  | 77.72 | 9.52 | Abcb8 | 362302 | Q5RKI8 | 81910043 | 0.149 | 0.039 |  |  | | | | | | | | | | | | | | | | | |
| Q5XIH3 | NADH dehydrogenase (Ubiquinone) flavoprotein 1 | 240 | 72.63 | 28 | 1.02 | 0.96 | 1 | 0.93 | 0.98 | 0.04 | 50.7 | 8.07 | Ndufv1 | 293655 | Q5XIH3 | 81883773 | -0.272 | 1.519 | 1 |  | | | | | | | | | | | | | | | | | |
| Q6AXV4 | Sorting and assembly machinery component 50 homolog | 44.17 | 24.31 | 8 | 0.92 | 1 | 0.97 | 1.02 | 0.98 | 0.04 | 51.93 | 6.8 | Samm50 | 300111 | Q6AXV4 | 81884480 | -0.189 | 0.27 | 1 |  | | | | | | | | | | | | | | | | | |
| P21913 | Succinate dehydrogenase [ubiquinone] iron-sulfur subunit, mitochondrial | 99.18 | 57.8 | 23 | 0.99 | 0.97 | 0.98 | 0.96 | 0.98 | 0.01 | 31.81 | 8.68 | Sdhb | 298596 | P21913 | 205371749 | -0.37 | 1.572 | 1 |  | | | | | | | | | | | | | | | | | |
| P21571 | ATP synthase-coupling factor 6, mitochondrial | 68.97 | 55.56 | 8 | 1.03 | 1.01 | 0.88 | 0.99 | 1.02 | 0.06 | 12.49 | 9.44 | Atp5j | 94271 | P21571 | 114690 | -0.546 | 1.522 | 1 |  | | | | | | | | | | | | | | | | | |
| D3ZZY4 | Protein LOC100360426 | 1.81 | 29.41 | 2 |  |  | 0.96 | 1 | 0.98 |  | 6.03 | 9.6 | LOC100360426 | 680080 | XP_003754060 | 672019283 | -0.002 | 0.331 |  |  | | | | | | | | | | | | | | | | | |
| B0BN52 | Mitochondrial carrier homolog 2 (C. elegans) | 70.62 | 36.96 | 9 | 0.96 | 0.98 | 0.92 | 1.06 | 0.98 | 0.06 | 33.51 | 8.25 | Mtch2 | 295922 | AAI58688.1 | 165971637 | 0.231 | 0.507 | 1 |  | | | | | | | | | | | | | | | | | |
| O55171 | Acyl-coenzyme A thioesterase 2, mitochondrial | 46.19 | 30.24 | 10 | 0.98 | 1.03 | 0.95 | 0.98 | 0.98 | 0.04 | 49.67 | 7.83 | Acot2 | 192272 | O55171 | 6166586 | -0.172 | 0.282 | 1 |  | | | | | | | | | | | | | | | | | |
| B2RYW3 | NADH dehydrogenase (Ubiquinone) 1 beta subcomplex, 9 | 53.22 | 64.8 | 10 | 0.98 | 0.99 | 1.05 | 0.92 | 0.99 | 0.05 | 21.88 | 8.07 | Ndufb9 | 299954 | NP_001120766 | 187937028 | -1.053 | 0.823 | 1 |  | | | | | | | | | | | | | | | | | |
| B2RZD6 | Ndufa4 protein | 61.96 | 86.59 | 9 | 0.98 | 0.99 | 0.96 | 1.02 | 0.99 | 0.03 | 9.32 | 9.52 | Ndufa4 | 681024 | NP_001121156 | 189085365 | -0.406 | 2.682 |  |  | | | | | | | | | | | | | | | | | |
| P19511 | ATP synthase subunit b, mitochondrial | 151.8 | 37.5 | 16 | 0.98 | 0.98 | 1.04 | 0.95 | 0.99 | 0.04 | 28.85 | 9.36 | Atp5f1 | 100911417,171375 | P19511 | 114625 | -0.137 | 1.802 | 5,7,11,12,16,1 |  | | | | | | | | | | | | | | | | | |
| P63031 | Mitochondrial pyruvate carrier 1 | 7.82 | 13.76 | 2 | 0.84 | 1.03 | 1.15 | 0.93 | 0.94 | 0.13 | 12.45 | 9.61 | Mpc1 | 171087 | P63031 | 52000889 | -0.024 | 0.321 |  |  | | | | | | | | | | | | | | | | | |
| Q68FY0 | Cytochrome b-c1 complex subunit 1, mitochondrial | 220.5 | 55 | 21 | 0.93 | 1 | 1 | 1.02 | 0.99 | 0.04 | 52.82 | 5.88 | Uqcrc1 | 301011 | Q68FY0 | 81884378 | -0.159 | 1.136 |  |  | | | | | | | | | | | | | | | | | |
| B2GUZ6 | Protein Rtn4ip1 | 12.65 | 11.36 | 4 | 0.96 | 0.97 | 1.11 | 0.91 | 0.99 | 0.09 | 43.58 | 9.2 | Rtn4ip1 | 309912 | NP_001101114 | 157821769 | 0.032 | 0.092 |  |  | | | | | | | | | | | | | | | | | |
| P35435 | ATP synthase subunit gamma, mitochondrial | 134.6 | 53.48 | 19 | 1.03 | 0.98 | 0.99 | 0.97 | 0.99 | 0.03 | 30.17 | 8.84 | Atp5c1 | 116550 | P35435 | 728931 | -0.221 | 1.591 | 5,11,12,16,1 |  | | | | | | | | | | | | | | | | | |
| P11608 | ATP synthase protein 8 | 18.23 | 40.3 | 4 | 0.93 | 1.05 | 0.89 | 1.08 | 0.99 | 0.09 | 7.64 | 9.32 | Mt-atp8 | 171374 | P11608 | 114491 | -0.093 | 1.44 | 1 |  | | | | | | | | | | | | | | | | | |
| Q5XIC0 | Enoyl-CoA delta isomerase 2, mitochondrial | 34.32 | 28.9 | 9 | 1.01 | 1.03 | 1.01 | 0.92 | 0.99 | 0.05 | 42.99 | 8.98 | Eci2 | 291075 | Q5XIC0 | 81883743 | -0.242 | 0.256 |  |  | | | | | | | | | | | | | | | | | |
| P84817-3 | Isoform 3 of Mitochondrial fission 1 protein | 25.74 | 37.93 | 6 | 0.98 | 0.8 | 1.08 | 1.11 | 0.99 | 0.14 | 16.24 | 9.25 | Fis1 | 288584 | XP_006249185 | 564378842 | -0.286 | 0.616 |  |  | | | | | | | | | | | | | | | | | |
| D3Z9R8 | 6.8 kDa mitochondrial proteolipid | 15.86 | 31.67 | 3 | 0.95 | 0.91 | 0.98 | 1.13 | 0.93 | 0.09 | 6.91 | 9.92 | Mp68 | 691427 | D3Z9R8 | 385178598 | -0.135 | 1.013 |  |  | | | | | | | | | | | | | | | | | |
| Q7TQ16 | Cytochrome b-c1 complex subunit 8 | 30.8 | 50 | 6 | 0.9 | 1.06 | 1.06 | 0.97 | 1 | 0.08 | 9.84 | 10.52 | Uqcrq | 497902 | Q7TQ16 | 81865392 | -0.656 | 1.524 |  |  | | | | | | | | | | | | | | | | | |
| Q9JM53 | Apoptosis-inducing factor 1, mitochondrial | 71.82 | 30.39 | 17 | 0.91 | 1.02 | 1.03 | 1.04 | 1 | 0.06 | 66.68 | 8.97 | Aifm1 | 83533 | Q9JM53 | 13431757 | -0.231 | 0.495 | 1 |  | | | | | | | | | | | | | | | | | |
| Q66HG9 | Mitochondrial antiviral-signaling protein | 13.65 | 12.43 | 4 | 0.98 | 1 | 1.1 | 0.91 | 1 | 0.08 | 53.77 | 6.46 | Mavs | 311430 | Q66HG9 | 81170680 | -0.337 | 0.074 |  |  | | | | | | | | | | | | | | | | | |
| Q5U2X6 | Coiled-coil domain-containing protein 47 | 4.53 | 3.93 | 2 |  |  | 1.05 | 0.95 | 1 |  | 55.7 | 4.84 | Ccdc47 | 303606 | Q5U2X6 | 81883524 | -0.759 | 0.036 |  |  | | | | | | | | | | | | | | | | | |
| D3ZFQ8 | Cytochrome c-1 (Predicted), isoform CRA_b | 13.71 | 8.76 | 3 | 0.97 | 1 | 0.99 | 1.06 | 1.01 | 0.04 | 23.01 | 10.27 | Cyc1 | 300047 | EDM15988 | 149066115 | 0.115 | 0.174 | 1 |  | | | | | | | | | | | | | | | | | |
| P13437 | 3-ketoacyl-CoA thiolase, mitochondrial | 242.6 | 71.03 | 2 | 1.05 | 1.02 | 0.94 | 1.02 | 1.01 | 0.05 | 41.84 | 7.94 | Acaa2 | 170465 | P13437 | 135762 | -0.044 | 1.506 |  |  | | | | | | | | | | | | | | | | | |
| D4A5L9 | Protein LOC679794 | 67.22 | 67.62 | 15 | 1.05 | 0.98 | 0.99 | 1.01 | 1.01 | 0.03 | 11.63 | 9.58 | LOC690675 | 25309 | NP_036971 | 6978725 | -0.781 | 2.666 |  |  | | | | | | | | | | | | | | | | | |
| P23965 | Enoyl-CoA delta isomerase 1, mitochondrial | 35.63 | 21.8 | 5 | 1 | 1 | 0.94 | 1.09 | 1.01 | 0.06 | 32.23 | 9.54 | Eci1 | 29740 | P23965 | 118210 | -0.156 | 0.31 |  |  | | | | | | | | | | | | | | | | | |
| P97521 | Mitochondrial carnitine/acylcarnitine carrier protein | 13.23 | 17.94 | 5 | 0.94 | 1.05 | 0.95 | 1.12 | 1.01 | 0.09 | 33.13 | 9.48 | Slc25a20 | 117035 | P97521 | 2497984 | 0.084 | 0.151 |  |  | | | | | | | | | | | | | | | | | |
| Q5XIJ3 | Isocitrate dehydrogenase 3 (NAD), gamma | 47.91 | 26.21 | 7 | 1.03 | 1.02 | 1.05 | 0.96 | 1.01 | 0.04 | 42.82 | 9.01 | Idh3g | 25179 | Q5XIJ3 | 81883787 | -0.087 | 0.35 | 1 |  | | | | | | | | | | | | | | | | | |
| P10888 | Cytochrome c oxidase subunit 4 isoform 1, mitochondrial | 105.6 | 68.05 | 18 | 1 | 0.96 | 1.1 | 0.99 | 1.01 | 0.06 | 19.5 | 9.44 | Cox4i1 | 29445 | P10888 | 117089 | -0.37 | 2.307 | 5,16,1 |  | | | | | | | | | | | | | | | | | |
| Q5U1Z9 | Metaxin 2 | 26.47 | 21.67 | 5 | 1.11 | 1.02 | 0.94 | 1 | 1.02 | 0.07 | 29.7 | 5.34 | Mtx2 | 288150 | Q5U1Z9 | 81883451 | -0.167 | 0.269 |  |  | | | | | | | | | | | | | | | | | |
| D3ZAQ0 | Protein Fundc2 | 8.74 | 25.83 | 4 | 0.95 | 0.97 | 1.02 | 1.14 | 1.02 | 0.09 | 16.33 | 10.05 | Fundc2 | 361288 | NP_001129229 | 209364562 | -0.228 | 0.306 |  |  | | | | | | | | | | | | | | | | | |
| Q8SEZ0 | NADH-ubiquinone oxidoreductase chain 5 | 3.89 | 2.96 | 2 |  |  | 0.97 | 1.07 | 1.02 |  | 68.57 | 9.26 | Mt-nd5 | 26202 | Q8SEZ0 | 81915096 | 0.617 | 0.029 | 5 |  | | | | | | | | | | | | | | | | | |
| B1WC61 | Acad9 protein | 14.08 | 7.52 | 5 | 0.99 | 0.99 | 1.03 | 1.1 | 1.03 | 0.05 | 68.8 | 7.69 | Acad9 | 294973 | NP_861433 | 197313734 | -0.052 | 0.087 |  |  | | | | | | | | | | | | | | | | | |
| F1M953 | Stress-70 protein, mitochondrial | 148.2 | 43.45 | 28 | 1.07 | 1.05 | 0.97 | 1.02 | 1.03 | 0.04 | 73.7 | 6.16 | Hspa9 | 291671 | NP_001094128 | 410110929 | -0.414 | 0.706 |  |  | | | | | | | | | | | | | | | | | |
| F1LM47 | Protein Sucla2 | 111.3 | 50.32 | 24 | 1.04 | 0.99 | 1.06 | 1.02 | 1.03 | 0.03 | 50.27 | 7.69 | Sucla2 | 361071 | NP_001101857 | 158749584 | -0.023 | 0.915 |  |  | | | | | | | | | | | | | | | | | |
| Q5XIN6 | LETM1 and EF-hand domain-containing protein 1, mitochondrial | 20.89 | 11.37 | 7 | 1.17 | 0.97 | 1.05 | 0.93 | 1.03 | 0.1 | 83.01 | 6.6 | Letm1 | 305457 | Q5XIN6 | 62510718 | -0.413 | 0.084 |  |  | | | | | | | | | | | | | | | | | |
| Q7TQ85 | Ac1164 | 36.97 | 17.44 | 9 | 0.99 | 1.07 | 0.99 | 1.1 | 1.04 | 0.06 | 58.76 | 8.53 | Pdhx | 311254 | Q7TQ85 | 81871023 | -0.118 | 0.221 |  |  | | | | | | | | | | | | | | | | | |
| Q75Q39 | Mitochondrial import receptor subunit TOM70 | 10.49 | 7.54 | 4 | 1.03 | 1.1 | 1.06 | 0.97 | 1.06 | 0.06 | 67.4 | 7.5 | Tomm70a | 304017 | Q75Q39 | 81911805 | -0.498 | 0.059 |  |  | | | | | | | | | | | | | | | | | |
| P80432 | Cytochrome c oxidase subunit 7C, mitochondrial | 32.44 | 41.27 | 4 | 1.03 | 1.12 | 1.08 | 0.92 | 1.04 | 0.08 | 7.37 | 11 | Cox7c | 100188937 | P80432 | 353526223 | -0.114 | 1.9 | 5,1 |  | | | | | | | | | | | | | | | | | |
| Q5XIK2 | Thioredoxin-related transmembrane protein 2 | 10.97 | 13.56 | 4 | 1.08 | 1.08 | 1 | 1 | 1.04 | 0.04 | 33.84 | 8.87 | Tmx2 | 295701 | Q5XIK2 | 81883793 | -0.134 | 0.207 |  |  | | | | | | | | | | | | | | | | | |
| Q6PDU7 | ATP synthase subunit g, mitochondrial | 66.8 | 46.6 | 4 | 1.03 | 1.07 | 1.05 | 1.01 | 1.04 | 0.03 | 11.43 | 9.58 | Atp5l | 300677 | Q6PDU7 | 385178701 | 0.243 | 1.488 | 5 |  | | | | | | | | | | | | | | | | | |
| Q06647 | ATP synthase subunit O, mitochondrial | 121.1 | 72.3 | 16 | 1.09 | 0.99 | 1.05 | 1.03 | 1.04 | 0.04 | 23.38 | 10.02 | Atp5o | 192241 | Q06647 | 543880 | -0.022 | 1.967 |  |  | | | | | | | | | | | | | | | | | |
| P38718-2 | Isoform Short of Mitochondrial pyruvate carrier 2 | 17.19 | 34.48 | 3 | 0.89 | 1.05 | 1.08 | 1.15 | 1.04 | 0.11 | 12.96 | 10.77 | Mpc2 | 100359982 | NP_001071111 | 117647218 | 0.129 | 0.772 |  |  | | | | | | | | | | | | | | | | | |
| D3ZL85 | Protein Hccs | 5 | 8.76 | 2 | 0 | 0 | 1.07 | 1.02 | 1.04 | 0.03 | 31.11 | 6.73 | Hccs | 317444 | NP_001178661 | 300795323 | -0.797 | 0.096 |  |  | | | | | | | | | | | | | | | | | |
| Q4QQV3 | Protein FAM162A | 20.65 | 39.35 | 6 | 1.04 | 1.01 | 1.04 | 1.09 | 1.03 | 0.03 | 17.82 | 9.99 | Fam162a | 360721 | Q4QQV3 | 81918167 | -0.512 | 0.73 |  |  | | | | | | | | | | | | | | | | | |
| G3V6H5 | Mitochondrial 2-oxoglutarate/malate carrier protein | 36.64 | 36.62 | 11 |  |  | 1.03 | 1.06 | 1.05 |  | 34.11 | 9.96 | Slc25a11 | 64201 | EDM05029 | 149053212 | 0.086 | 0.44 |  |  | | | | | | | | | | | | | | | | | |
| G3V6H5 | Mitochondrial 2-oxoglutarate/malate carrier protein | 49.46 | 46.18 | 12 | 1.03 | 1.08 | 1.03 | 1.06 | 1.05 | 0.02 | 34.11 | 9.96 | Slc25a11 | 64201 | EDM05029 | 149053212 | 0.086 | 0.44 |  |  | | | | | | | | | | | | | | | | | |
| B2RYS8 | NADH dehydrogenase (Ubiquinone) 1 beta subcomplex 8 | 55.53 | 49.46 | 6 | 1.09 | 1.04 | 1.03 | 1.06 | 1.05 | 0.03 | 21.94 | 6.05 | Ndufb8 | 293991 | NP_001099830 | 157822261 | -0.705 | 0.729 | 1 |  | | | | | | | | | | | | | | | | | |
| D3ZEN5 | Peroxiredoxin-5, mitochondrial (Fragment) | 21.73 | 42.24 | 5 | 0.99 | 1.17 | 0.95 | 1.12 | 1.06 | 0.1 | 16.91 | 7.24 | Prdx5 | 113898 | AAH78771 | 51261175 | 0.074 | 0.355 |  |  | | | | | | | | | | | | | | | | | |
| Q5PPN7 | Coiled-coil domain-containing protein 51 | 4.8 | 4.88 | 2 | 1 | 1.12 |  |  | 1.06 |  | 45.78 | 8.09 | Ccdc51 | 316008 | Q5PPN7 | 148840310 | -0.415 | 0.044 | 1 |  | | | | | | | | | | | | | | | | | |
| Q641Z9 | Protein Sdhc | 6.58 | 11.83 | 2 | 0.98 | 1.16 |  |  | 1.07 | 0 | 18.19 | 10.21 | Sdhc | 289217 | Q641Z9 | 81884084 | 0.547 | 0.11 |  |  | | | | | | | | | | | | | | | | | |
| P29266 | 3-hydroxyisobutyrate dehydrogenase, mitochondrial | 42.63 | 22.99 | 5 | 1 | 1.05 | 1.12 | 1.11 | 1.03 | 0.06 | 35.28 | 8.47 | Hibadh | 63938 | P29266 | 12585554 | 0.031 | 0.312 |  |  | | | | | | | | | | | | | | | | | |
| F1LMM8 | [Pyruvate dehydrogenase [lipoamide]] kinase isozyme 2, mitochondrial | 7.84 | 9.69 | 2 | 1.05 | 1.09 |  |  | 1.07 |  | 43.99 | 7.36 | Pdk2 | 81530 | AAF81193 | 8895958 | -0.201 | 0.068 | 1 |  | | | | | | | | | | | | | | | | | |
| B2GV15 | Dihydrolipoamide branched chain transacylase E2 | 30.04 | 19.71 | 8 | 1.02 | 1.16 | 1.02 | 1.1 | 1.07 | 0.07 | 53.24 | 8.41 | Dbt | 29611 | NP_445764 | 158749632 | -0.157 | 0.225 |  |  | | | | | | | | | | | | | | | | | |
| P17764 | Acetyl-CoA acetyltransferase, mitochondrial | 231.4 | 67.92 | 26 | 1.07 | 1.05 | 1.11 | 1.08 | 1.08 | 0.03 | 44.67 | 8.76 | Acat1 | 25014 | P17764 | 135757 | 0.086 | 1.612 |  |  | | | | | | | | | | | | | | | | | |
| P97852 | Peroxisomal multifunctional enzyme type 2 | 62.79 | 25.71 | 15 | 1.18 | 1.07 | 1.03 | 1.07 | 1.09 | 0.06 | 79.38 | 8.57 | Hsd17b4 | 79244 | P97852 | 2492741 | -0.104 | 0.265 | 1 |  | | | | | | | | | | | | | | | | | |
| F1LNF7 | Isocitrate dehydrogenase [NAD] subunit alpha, mitochondrial | 65.91 | 48.09 | 18 | 1.13 | 1.09 | 1.08 | 1.05 | 1.09 | 0.03 | 39.56 | 6.92 | Idh3a | 114096 | EDL95540 | 149041699 | -0.068 | 0.758 | 1 |  | | | | | | | | | | | | | | | | | |
| F1LP30 | Methylcrotonoyl-CoA carboxylase subunit alpha, mitochondrial | 7.71 | 4.9 | 3 | 1.04 | 1.3 | 0.99 | 1.02 | 1.09 | 0.14 | 79.25 | 7.14 | Mccc1 | 294972 | NP_001009653 | 57528264 | -0.287 | 0.05 |  |  | | | | | | | | | | | | | | | | | |
| D4A565 | NADH dehydrogenase (Ubiquinone) 1 beta subcomplex, 5 (Predicted), isoform CRA_b | 58.29 | 30.69 | 7 | 1.1 | 1.14 | 1.03 | 1.1 | 1.12 | 0.04 | 21.65 | 9.47 | Ndufb5 | 294964 | NP_001099896 | 157823387 | -0.252 | 0.785 | 1 |  | | | | | | | | | | | | | | | | | |
| Q499N5 | Acyl-CoA synthetase family member 2, mitochondrial | 19.1 | 12.52 | 7 | 1.03 | 1.12 | 1.15 | 1.08 | 1.09 | 0.05 | 67.84 | 8.12 | Acsf2 | 619561 | Q499N5 | 123781622 | -0.153 | 0.147 | 7,8,10,12,13,1 |  | | | | | | | | | | | | | | | | | |
| G3V6P2 | Dihydrolipoamide S-succinyltransferase (E2 component of 2-oxo-glutarate complex), isoform CRA_a | 86.55 | 24.67 | 11 | 1.16 | 1.08 | 1.18 | 0.95 | 1.09 | 0.1 | 48.87 | 8.7 | Dlst | 299201 | NP_001006982 | 195927000 | -0.165 | 0.655 | 1 |  | | | | | | | | | | | | | | | | | |
| P29117 | Peptidyl-prolyl cis-trans isomerase F, mitochondrial | 17.95 | 21.84 | 6 | 1.12 | 1.1 | 1.07 | 1.09 | 1.09 | 0.02 | 21.8 | 9.14 | Ppif | 282819 | P29117 | 2507228 | -0.164 | 0.413 |  |  | | | | | | | | | | | | | | | | | |
| P26772 | 10 kDa heat shock protein, mitochondrial | 51.19 | 80.39 | 10 | 1.09 | 1.07 | 1.16 | 1.08 | 1.1 | 0.04 | 10.89 | 8.92 | Hspe1 | 25462 | P26772 | 461731 | -0.037 | 1.928 |  |  | | | | | | | | | | | | | | | | | |
| P13086 | Succinyl-CoA ligase [ADP/GDP-forming] subunit alpha, mitochondrial | 113.2 | 34.97 | 17 | 1.08 | 1.14 | 1.14 | 1.05 | 1.1 | 0.05 | 36.12 | 9.48 | Suclg1 | 114597 | P13086 | 223634703 | -0.028 | 1.135 |  |  | | | | | | | | | | | | | | | | | |
| F1LN92 | Protein Afg3l2 | 11.93 | 5.99 | 5 | 1.11 | 1.07 | 1.2 | 1.04 | 1.11 | 0.07 | 89.3 | 8.6 | Afg3l2 | 307350 | NP_001128336 | 198442897 | -0.359 | 0.078 |  |  | | | | | | | | | | | | | | | | | |
| Q03344 | ATPase inhibitor, mitochondrial | 47.19 | 39.25 | 4 | 1.16 | 0.96 | 1.15 | 1.17 | 1.06 | 0.1 | 12.24 | 9.6 | Atpif1 | 25392 | Q03344 | 1352410 | -1.106 | 1.797 |  |  | | | | | | | | | | | | | | | | | |
| Q68FX0 | Isocitrate dehydrogenase [NAD] subunit beta, mitochondrial | 90.05 | 42.86 | 15 | 1.09 | 1.23 | 1.05 | 1.11 | 1.12 | 0.08 | 42.33 | 8.75 | Idh3B | 94173 | Q68FX0 | 68051964 | -0.133 | 0.685 | 1 |  | | | | | | | | | | | | | | | | | |
| P15650 | Long-chain specific acyl-CoA dehydrogenase, mitochondrial | 173.7 | 42.33 | 20 | 1.2 | 1.08 | 1.14 | 1.07 | 1.12 | 0.06 | 47.84 | 7.74 | Acadl | 25287 | P15650 | 113016 | -0.223 | 1.254 | 16 |  | | | | | | | | | | | | | | | | | |
| P13803 | Electron transfer flavoprotein subunit alpha, mitochondrial | 146.9 | 63.96 | 16 | 1.16 | 1.14 | 1.12 | 1.07 | 1.12 | 0.04 | 34.93 | 8.38 | Etfa | 300726 | P13803 | 122065179 | 0.131 | 1.088 | 5 |  | | | | | | | | | | | | | | | | | |
| Q68FZ8 | Propionyl coenzyme A carboxylase, beta polypeptide | 54.55 | 36.78 | 2 | 1.18 | 1.11 | 1.1 | 1.12 | 1.13 | 0.04 | 58.64 | 7.84 | Pccb | 24624 | Q68FZ8 | 81884384 | 0.01 | 0.324 | 4,7 |  | | | | | | | | | | | | | | | | | |
| Q6P6R2 | Dihydrolipoyl dehydrogenase, mitochondrial | 154.9 | 45.19 | 18 | 1.15 | 1.06 | 1.16 | 1.15 | 1.13 | 0.04 | 54 | 7.87 | Dld | 298942 | Q6P6R2 | 81885266 | -0.01 | 0.87 |  |  | | | | | | | | | | | | | | | | | |
| Q5XI78 | 2-oxoglutarate dehydrogenase, mitochondrial | 235.8 | 37.24 | 38 | 1.16 | 1.08 | 1.13 | 1.17 | 1.13 | 0.04 | 116.2 | 6.77 | Ogdh | 360975 | Q5XI78 | 81883712 | -0.342 | 0.628 | 1 |  | | | | | | | | | | | | | | | | | |
| P14604 | Enoyl-CoA hydratase, mitochondrial | 90.05 | 51.38 | 15 | 1.12 | 1.08 | 1.18 | 1.18 | 1.1 | 0.05 | 31.5 | 8.13 | Echs1 | 100911186,140547 | P14604 | 119119 | -0.104 | 1.111 |  |  | | | | | | | | | | | | | | | | | |
| Q68FU3 | Electron transfer flavoprotein subunit beta | 97.25 | 64.71 | 18 | 1.11 | 1.14 | 1.18 | 1.16 | 1.15 | 0.03 | 27.67 | 7.75 | Etfb | 292845 | Q68FU3 | 81884360 | -0.076 | 1.301 |  |  | | | | | | | | | | | | | | | | | |
| P12007 | Isovaleryl-CoA dehydrogenase, mitochondrial | 29.39 | 16.98 | 7 | 1.25 | 1.12 | 1.11 | 1.12 | 1.15 | 0.07 | 46.41 | 7.9 | Ivd | 24513 | P12007 | 125052 | -0.113 | 0.237 |  |  | | | | | | | | | | | | | | | | | |
| F1LM33 | Leucine-rich PPR motif-containing protein, mitochondrial | 9.38 | 3.02 | 4 | 1.06 | 1.25 |  |  | 1.15 |  | 156.6 | 6.61 | Lrpprc | 313867 | EDM02690 | 149050517 | -0.185 | 0.026 | 1 |  | | | | | | | | | | | | | | | | | |
| P15651 | Short-chain specific acyl-CoA dehydrogenase, mitochondrial | 85.76 | 48.06 | 14 | 1.15 | 1.16 | 1.14 | 1.16 | 1.15 | 0.01 | 44.74 | 8.28 | Acads | 64304 | P15651 | 1168286 | -0.146 | 0.536 |  |  | | | | | | | | | | | | | | | | | |
| Q9ER34 | Aconitate hydratase, mitochondrial | 347.5 | 55.51 | 44 | 1.22 | 1.08 | 1.21 | 1.12 | 1.16 | 0.07 | 85.38 | 7.83 | Aco2 | 79250 | Q9ER34 | 60391194 | -0.334 | 1.347 |  |  | | | | | | | | | | | | | | | | | |
| M0RDK9 | Protein Acad8 (Fragment) | 7.1 | 7.4 | 2 | 1.2 | 1.12 |  |  | 1.16 |  | 33.28 | 8.4 | Acad8 | 367196 | XP_003754442 | 392341831 | -0.086 | 0.06 |  |  | | | | | | | | | | | | | | | | | |
| Q5BJZ3 | Nicotinamide nucleotide transhydrogenase | 272.2 | 41.9 | 44 | 1.14 | 1.19 | 1.17 | 1.15 | 1.17 | 0.02 | 113.8 | 7.88 | Nnt | 310378 | Q5BJZ3 | 81882562 | 0.274 | 0.808 | 1 |  | | | | | | | | | | | | | | | | | |
| D3ZX74 | Coenzyme Q10 homolog A (Yeast) (Predicted), isoform CRA_b | 4.78 | 11.06 | 2 | 1.13 | 1.29 | 1.05 | 1.19 | 1.17 | 0.1 | 24.68 | 8.94 | Coq10a | 362810 | NP_001102197 | 157823565 | -0.047 | 0.081 |  |  | | | | | | | | | | | | | | | | | |
| G3V7I0 | Peroxiredoxin 3 | 38.88 | 30.35 | 6 | 1.23 | 1.19 | 1.23 | 1.09 | 1.18 | 0.07 | 28.28 | 7.55 | Prdx3 | 64371 | EDL94585 | 149040547 | 0.03 | 0.389 | 1 |  | | | | | | | | | | | | | | | | | |
| G3V9U2 | 3-ketoacyl-CoA thiolase, mitochondrial | 251.9 | 75.76 | 4 | 1.23 | 1.19 | 1.23 | 1.1 | 1.19 | 0.06 | 41.76 | 8.15 | Acaa2 | 170465 | EDL82880 | 149027156 | -0.043 | 1.628 |  |  | | | | | | | | | | | | | | | | | |
| Q09073 | ADP/ATP translocase 2 | 146.1 | 44.63 | 6 | 1.28 | 1.12 | 1.15 | 1.28 | 1.21 | 0.09 | 32.88 | 9.73 | Slc25a5 | 25176 | Q09073 | 728810 | 0.021 | 1.582 | 1 |  | | | | | | | | | | | | | | | | | |
| O88989 | Malate dehydrogenase, cytoplasmic | 60.15 | 47.6 | 13 | 1.33 | 1.18 | 1.23 | 1.1 | 1.21 | 0.09 | 36.46 | 6.58 | Mdh1 | 24551 | O88989 | 81861572 | -0.059 | 0.576 | 16,1 |  | | | | | | | | | | | | | | | | | |
| G3V936 | Citrate synthase | 93.66 | 38.63 | 13 | 1.16 | 1.27 | 1.14 | 1.29 | 1.22 | 0.08 | 51.8 | 8.41 | Cs | 170587 | EDL84868 | 149029697 | -0.176 | 0.579 | 1 |  | | | | | | | | | | | | | | | | | |
| P11980 | Pyruvate kinase isozymes M1/M2 | 26.33 | 21.28 | 9 | 1.27 | 1.24 | 1.17 | 1.2 | 1.25 | 0.04 | 57.78 | 7.06 | Pkm | 25630 | P11980 | 125601 | -0.093 | 0.19 | 7,16 |  | | | | | | | | | | | | | | | | | |
| G3V741 | Phosphate carrier protein, mitochondrial | 55.96 | 26.69 | 11 | 1.14 | 1.31 | 1.19 | 1.26 | 1.22 | 0.08 | 39.51 | 9.26 | Slc25a3 | 245959 | NP_620800 | 399124780 | 0.052 | 0.861 |  |  | | | | | | | | | | | | | | | | | |
| P00507 | Aspartate aminotransferase, mitochondrial | 158.5 | 54.42 | 23 | 1.3 | 1.19 | 1.22 | 1.18 | 1.23 | 0.05 | 47.28 | 9 | Got2 | 25721 | P00507 | 112987 | -0.23 | 1.121 | 1 |  | | | | | | | | | | | | | | | | | |
| P14408-2 | Isoform Cytoplasmic of Fumarate hydratase, mitochondrial | 58.05 | 34.26 | 12 | 1.35 | 1.35 | 1.24 | 0.98 | 1.23 | 0.18 | 50.14 | 8.19 | Fh | 24368 | P14408 | 120605 | -0.09 | 0.379 | 1 |  | | | | | | | | | | | | | | | | | |
| P04636 | Malate dehydrogenase, mitochondrial | 297.2 | 63.02 | 21 | 1.25 | 1.22 | 1.25 | 1.23 | 1.24 | 0.02 | 35.66 | 8.68 | Mdh2 | 81829 | P04636 | 122065494 | 0.119 | 2.215 | 16,1 |  | | | | | | | | | | | | | | | | | |
| P07895 | Superoxide dismutase [Mn], mitochondrial | 35.33 | 35.59 | 8 | 1.31 | 1.2 | 1.23 | 1.21 | 1.24 | 0.05 | 24.66 | 8.81 | Sod2 | 24787 | P07895 | 134678 | -0.422 | 0.568 |  |  | | | | | | | | | | | | | | | | | |
| P11884 | Aldehyde dehydrogenase, mitochondrial | 131.2 | 44.89 | 19 | 1.25 | 1.23 | 1.21 | 1.29 | 1.24 | 0.04 | 56.45 | 7.05 | Aldh2 | 29539 | P11884 | 118505 | -0.138 | 0.815 | 5,12,16,1 |  | | | | | | | | | | | | | | | | | |
| Q9Z0J5-2 | Isoform 2 of Thioredoxin reductase 2, mitochondrial | 8.93 | 10.39 | 2 |  |  | 1.2 | 1.3 | 1.25 |  | 53.21 | 7.71 | Txnrd2 | 50551 | NP_072106 | 12018236 | -0.112 | 0.038 |  |  | | | | | | | | | | | | | | | | | |
| P29147 | D-beta-hydroxybutyrate dehydrogenase, mitochondrial | 50.71 | 34.4 | 10 | 1.26 | 1.21 | 1.38 | 1.24 | 1.27 | 0.07 | 38.18 | 8.84 | Bdh1 | 117099 | P29147 | 68837285 | -0.242 | 0.471 |  |  | | | | | | | | | | | | | | | | | |
| Q924S5 | Lon protease homolog, mitochondrial | 7.3 | 2.21 | 2 | 1.25 | 1.35 |  |  | 1.3 |  | 105.7 | 6.6 | Lonp1 | 170916 | Q924S5 | 81916424 | -0.287 | 0.019 |  |  | | | | | | | | | | | | | | | | | |
| P56571 | ES1 protein homolog, mitochondrial | 92.55 | 59.77 | 12 | 1.32 | 1.23 | 1.51 | 1.24 | 1.32 | 0.13 | 28.15 | 8.92 | RGD1311648 | 294326 | P56571 | 83302472 | -0.067 | 0.923 |  |  | | | | | | | | | | | | | | | | | |
| P36970-2 | Isoform Cytoplasmic of Phospholipid hydroperoxide glutathione peroxidase, mitochondrial | 55.03 | 54.12 | 11 | 1.45 | 1.51 | 1.32 | 1.12 | 1.35 | 0.17 | 19.48 | 8 | Gpx4 | 29328 | NP_058861 | 90903249 | -0.23 | 1.232 |  |  | | | | | | | | | | | | | | | | | |
| P56574 | Isocitrate dehydrogenase [NADP], mitochondrial | 326 | 62.61 | 35 | 1.44 | 1.29 | 1.42 | 1.29 | 1.36 | 0.08 | 50.93 | 8.69 | Idh2 | 361596 | P56574 | 119364595 | -0.408 | 2.336 | 1 |  | | | | | | | | | | | | | | | | | |
| B2GV06 | Succinyl-CoA:3-ketoacid coenzyme A transferase 1, mitochondrial | 138.3 | 50 | 19 | 1.51 | 1.47 | 1.57 | 1.42 | 1.49 | 0.07 | 56.17 | 8.47 | Oxct1 | 690163 | B2GV06 | 205829936 | -0.115 | 0.712 |  |  | | | | | | | | | | | | | | | | | |
| P20070-3 | Isoform 3 of NADH-cytochrome b5 reductase 3 | 332.1 | 78.78 | 18 | 1.75 | 1.72 | 1.75 | 1.64 | 1.72 | 0.05 | 31.57 | 8.15 | Cyb5r3 | 25035 | NP_620232 | 20302049 | -0.213 | 3.262 | 10,15 |  | | | | | | | | | | | | | | | | | |
| Q5M840 | G0/G1 switch protein 2 | 27 | 37.86 | 4 | 2.57 | 2.56 | 2.53 | 2.37 | 2.51 | 0.09 | 11.22 | 7.88 | G0s2 | 289388 | Q5M840 | 81883083 | 0.081 | 0.891 |  |  | | | | | | | | | | | | | | | | | |
| O08776-4 | Isoform 4 of NADH dehydrogenase | 4.63 | 11.73 | 2 |  |  |  |  |  |  | 19.9 | 7.84 | Ndufaf3 |  | O08776-4 | 77157788 | -0.282 | 0.011 |  |  | | | | | | | | | | | | | | | | | |
| **other proteins** | | | | | | | | | | | | | | | | | | |  | |  |  |  |  |  |  |  |  |  |  |  |  |  |  |  |  |  |
| F1LM84 | Nidogen-1 | 11.62 | 3.29 | 4 | 0.54 | 0.61 | 0.56 | 0.62 | 0.58 | 0.03 | 137 | 5.47 | Nid1 | 25494 | XP_213954 | 109505096 | -0.381 | 0.029 |  |  | | | | | | | | | | | | | | | | | |
| O08590 | Membrane primary amine oxidase | 31.38 | 14.02 | 9 | 0.56 | 0.57 | 0.62 | 0.57 | 0.58 | 0.03 | 84.93 | 6.49 | Aoc3 | 29473 | O08590 | 84028175 | -0.171 | 0.118 |  |  | | | | | | | | | | | | | | | | | |
| F1M705 | Uncharacterized protein | 305 | 37.79 | 69 | 0.64 | 0.66 | 0.65 | 0.66 | 0.65 | 0.01 | 245.6 | 5.99 |  | 103690836 | XP_008771503 | 672087714 | -0.711 | 0.448 |  |  | | | | | | | | | | | | | | | | | |
| G3V7C6 | RCG45400 | 339.1 | 74.38 | 4 | 0.57 | 0.64 | 0.67 | 0.77 | 0.6 | 0.08 | 49.8 | 4.89 | Tubb4b | 296554 | XP_006233644 | 564339503 | -0.362 | 2.088 |  |  | | | | | | | | | | | | | | | | | |
| Q499Q2 | Protein Tpd52l1 | 29.28 | 34.97 | 6 | 0.61 | 0.75 | 0.6 | 0.75 | 0.68 | 0.08 | 18.34 | 6.4 | Tpd52l1 | 689256 | Q499Q2 | 123781629 | -0.617 | 0.764 |  |  | | | | | | | | | | | | | | | | | |
| D3ZEK8 | Uncharacterized protein | 42.56 | 17.78 | 5 | 0.64 | 0.76 | 0.64 | 0.71 | 0.69 | 0.06 | 42.12 | 6.6 |  | 103690839 | XP_008756652 | 672032318 | -0.732 | 0.332 |  |  | | | | | | | | | | | | | | | | | |
| M0R6D4 | Uncharacterized protein | 13.33 | 31.21 | 5 | 0.69 | 0.65 | 0.75 | 0.73 | 0.7 | 0.05 | 18.03 | 5.08 |  | 24907 | EDL96057 | 149042350 | -0.757 | 0.333 |  |  | | | | | | | | | | | | | | | | | |
| Q52KS1 | 6-phosphofructokinase | 95.76 | 30.26 | 21 | 0.69 | 0.69 | 0.68 | 0.75 | 0.7 | 0.03 | 85.29 | 7.88 | Pfkm | 65152 | Q52KS1 | 81882300 | -0.183 | 0.363 | 1 |  | | | | | | | | | | | | | | | | | |
| G3V879 | Ubiquinone biosynthesis protein COQ7 homolog | 21.83 | 26.82 | 4 | 0.7 | 0.74 | 0.95 | 0.62 | 0.75 | 0.14 | 20.11 | 5.76 | Coq7 | 25249 | NP_036917 | 472235294 | -0.165 | 0.398 |  |  | | | | | | | | | | | | | | | | | |
| Q05BA4 | Myadm protein | 6.64 | 9.43 | 2 | 0.63 | 0.91 | 0.69 | 0.86 | 0.77 | 0.14 | 35.1 | 8.31 | Myadm | 369016 | Q05BA4 | 123795808 | 0.657 | 0.057 |  |  | | | | | | | | | | | | | | | | | |
| D4A1G4 | Cytochrome b5 | 57.55 | 70 | 7 | 0.77 | 0.84 | 0.7 | 0.79 | 0.81 | 0.06 | 11.47 | 5.71 | Cyb5a | 64001 | AAB67609 | 2257955 | -1.017 | 1.743 | 4,7,10,14,15,1 |  | | | | | | | | | | | | | | | | | |
| D4AAI5 | Cullin-associated NEDD8-dissociated protein 2 | 38.67 | 10.12 | 10 |  |  | 0.76 | 0.81 | 0.78 |  | 135.6 | 5.58 | Cand2 | 192226 | EDM02145 | 149049691 | -0.002 | 0.096 |  |  | | | | | | | | | | | | | | | | | |
| D3ZM60 | Protein Ppp1r3a | 4.02 | 2.55 | 2 | 0.77 | 0.8 |  |  | 0.78 |  | 122.1 | 5.17 | Ppp1r3a | 500036 | NP_001102692 | 157822901 | -0.746 | 0.016 |  |  | | | | | | | | | | | | | | | | | |
| F1MAB9 | Protein Tpd52 | 13.22 | 19.2 | 3 | 0.87 | 0.79 | 0.66 | 0.83 | 0.79 | 0.09 | 24.25 | 4.72 | Tpd52 | 294900 | NP_001099891.1 | 157823391 | -0.723 | 0.165 |  |  | | | | | | | | | | | | | | | | | |
| Q5RK08 | Glioblastoma amplified sequence | 144.7 | 74.38 | 19 | 0.81 | 0.78 | 0.79 | 0.8 | 0.8 | 0.01 | 32.92 | 9.36 | Gbas | 498174 | Q5RK08 | 81883369 | -0.561 | 1.671 |  |  | | | | | | | | | | | | | | | | | |
| P02454 | Collagen alpha-1(I) chain | 8.26 | 3.44 | 3 | 0.71 | 0.83 | 0.81 | 0.84 | 0.8 | 0.06 | 137.9 | 5.92 | Col1a1 | 29393 | P02454 | 259016391 | -0.788 | 0.022 | 1 |  | | | | | | | | | | | | | | | | | |
| Q9R1T1 | Barrier-to-autointegration factor | 14 | 40.45 | 2 | 0.77 | 0.81 | 0.78 | 0.82 | 0.8 | 0.02 | 10.04 | 6.09 | Banf1 | 114087 | Q9R1T1 | 22095476 | -0.4 | 0.398 |  |  | | | | | | | | | | | | | | | | | |
| Q68FT3 | Pyridine nucleotide-disulfide oxidoreductase domain-containing protein 2 | 31.19 | 25.47 | 9 | 0.78 | 0.91 | 0.75 | 0.83 | 0.84 | 0.07 | 62.84 | 8.18 | Pyroxd2 | 309381 | Q68FT3 | 81884356 | -0.098 | 0.191 |  |  | | | | | | | | | | | | | | | | | |
| B0K020 | CDGSH iron-sulfur domain-containing protein 1 | 127.9 | 55.56 | 9 | 0.86 | 0.78 | 0.85 | 0.78 | 0.82 | 0.04 | 12.09 | 8.84 | Cisd1 | 294362 | B0K020 | 215275245 | -0.378 | 2.895 | 1 |  | | | | | | | | | | | | | | | | | |
| F1M7X5 | Dipeptidyl peptidase 4 | 6.97 | 4.22 | 3 | 0.73 | 0.9 | 0.77 | 0.91 | 0.83 | 0.09 | 84.12 | 6.1 | Dpp4 | 25253 | XP_008760101 | 672045733 | -0.353 | 0.036 |  |  | | | | | | | | | | | | | | | | | |
| M0RC66 | Adenylate kinase isoenzyme 1 (Fragment) | 28.54 | 31.61 | 6 |  |  | 0.82 | 0.84 | 0.83 |  | 21.28 | 9.16 | Ak1 | 24183 | NP_077325 | 61889092 | -0.47 | 0.423 | 1 |  | | | | | | | | | | | | | | | | | |
| D3ZUR9 | Uncharacterized protein (Fragment) | 12.08 | 5.76 | 4 | 0.82 | 1.02 | 0.63 | 0.86 | 0.83 | 0.16 | 117 | 5.6 | Lnpep | 171105 | NP_001106874 | 164663801 | -0.22 | 0.051 |  |  | | | | | | | | | | | | | | | | | |
| E9PT90 | Protein Spg20 | 8.05 | 7.38 | 3 | 0.83 | 0.86 | 0.91 | 0.74 | 0.83 | 0.07 | 64.72 | 5.35 | Spg20 | 295053 | XP_006232428 | 564336490 | -0.523 | 0.046 |  |  | | | | | | | | | | | | | | | | | |
| B5DEH2 | Erlin-2 | 49.32 | 38.64 | 13 | 0.83 | 0.88 | 0.77 | 0.86 | 0.83 | 0.05 | 37.69 | 5.74 | Erlin2 | 290823 | B5DEH2 | 229485399 | -0.172 | 0.504 |  |  | | | | | | | | | | | | | | | | | |
| P81155 | Voltage-dependent anion-selective channel protein 2 | 166.7 | 58.98 | 14 | 0.81 | 0.84 | 0.82 | 0.87 | 0.82 | 0.03 | 31.73 | 7.49 | Vdac2 | 83531 | P81155 | 46397780 | -0.221 | 1.702 | 5,15 |  | | | | | | | | | | | | | | | | | |
| Q6P2A5 | Adenylate kinase 3 | 21.88 | 35.68 | 7 | 0.87 | 0.77 | 0.86 | 0.84 | 0.84 | 0.05 | 25.48 | 8.85 | Ak3 | 26956 | Q6P2A5 | 81892174 | -0.266 | 0.314 | 5,1 |  | | | | | | | | | | | | | | | | | |
| Q5M9H2 | Acyl-Coenzyme A dehydrogenase, very long chain | 689.4 | 73.89 | 54 | 0.83 | 0.81 | 0.87 | 0.84 | 0.84 | 0.03 | 70.78 | 8.81 | Acadvl | 25363 | Q5M9H2 | 81889170 | -0.116 | 2.911 | 1 |  | | | | | | | | | | | | | | | | | |
| Q9WVJ6 | Protein Tgm2 | 21.35 | 10.06 | 6 | 0.85 | 0.84 | 0.79 | 0.86 | 0.84 | 0.03 | 76.89 | 5.1 | Tgm2 | 56083 | Q9WVJ6 | 81869912 | -0.353 | 0.104 |  |  | | | | | | | | | | | | | | | | | |
| Q5U302 | Catenin (Cadherin associated protein), alpha 1 | 12.97 | 4.19 | 3 | 0.79 | 0.89 | 0.84 | 0.83 | 0.84 | 0.04 | 100.2 | 6.23 | Ctnna1 | 307505 | Q5U302 | 81883536 | -0.374 | 0.04 | 1 |  | | | | | | | | | | | | | | | | | |
| Q9R0L4-3 | Isoform 3 of Cullin-associated NEDD8-dissociated protein 2 | 26.44 | 8.26 | 8 | 0.83 | 0.84 |  |  | 0.84 |  | 132.8 | 5.54 | Cand2 | 192226 | Q9R0L4 | 67460125 | 0.005 | 0.075 |  |  | | | | | | | | | | | | | | | | | |
| G3V7U4 | Lamin-B1 | 7.06 | 5.28 | 3 | 0.87 | 0.82 | 0.79 | 0.9 | 0.85 | 0.05 | 66.65 | 5.16 | Lmnb1 | 116685 | EDM14499 | 149064296 | -0.819 | 0.045 | 16,1 |  | | | | | | | | | | | | | | | | | |
| F1M9G8 | Histidine triad nucleotide-binding protein 3 | 18.39 | 28 | 4 | 0.87 | 0.87 | 0.82 | 0.82 | 0.85 | 0.03 | 19.72 | 6.71 | Hint3 | 246769 | NP_001094295 | 451172109 | -0.263 | 0.254 |  |  | | | | | | | | | | | | | | | | | |
| Q63083 | Nucleobindin-1 | 11.94 | 12.64 | 3 | 0.9 | 0.8 |  |  | 0.85 |  | 53.47 | 5.12 | Nucb1 | 84595 | Q63083 | 2493471 | -1.022 | 0.094 |  |  | | | | | | | | | | | | | | | | | |
| M0R6K0 | Laminin subunit beta-2 | 21.09 | 4.94 | 7 | 0.97 | 0.84 | 0.91 | 0.69 | 0.85 | 0.12 | 196.4 | 6.8 | Lamb2 | 25473 | XP_006243771 | 564365344 | -0.483 | 0.041 | 5 |  | | | | | | | | | | | | | | | | | |
| P00388 | NADPH--cytochrome P450 reductase | 17.46 | 10.77 | 6 | 0.88 | 0.85 | 0.85 | 0.88 | 0.86 | 0.02 | 76.91 | 5.48 | Por | 29441 | P00388 | 127966 | -0.433 | 0.078 | 5,1 |  | | | | | | | | | | | | | | | | | |
| B5DEI0 | Pcyox1l protein | 5.86 | 3.85 | 2 |  |  | 0.8 | 0.93 | 0.87 |  | 54.6 | 7.65 | Pcyox1l | 307396 | NP_001128014 | 197384556 | 0.056 | 0.037 |  |  | | | | | | | | | | | | | | | | | |
| F1LQN3 | Reticulon-4 | 9.48 | 4.04 | 5 |  |  | 0.8 | 0.93 | 0.87 |  | 126.3 | 4.49 | Rtn4 | 83765 | NP_114019 | 13929188 | -0.407 | 0.04 |  |  | | | | | | | | | | | | | | | | | |
| D4ABZ8 | Uncharacterized protein | 7.41 | 13.1 | 2 |  |  | 0.85 | 0.88 | 0 |  | 19.36 | 6.81 |  | 362495 | NP_942078 | 38454320 | -0.649 | 0.155 |  |  | | | | | | | | | | | | | | | | | |
| P15791-5 | Isoform Delta 5 of Calcium/calmodulin-dependent protein kinase type II subunit delta | 20 | 17.36 | 7 | 0.88 | 0.83 | 0.81 | 0.96 | 0.87 | 0.07 | 54.16 | 7.12 | Camk2d | 24246 | P15791.1 | 125288 | -0.424 | 0.148 |  |  | | | | | | | | | | | | | | | | | |
| F1LN35 | Dystrophin | 18.9 | 10.77 | 5 | 0.81 | 0.94 | 0.74 | 0.99 | 0.87 | 0.12 | 70.74 | 6.25 | Dmd | 24907 | NP_001005244 | 52630316 | -0.56 | 0.085 |  |  | | | | | | | | | | | | | | | | | |
| P14841 | Cystatin-C | 9.4 | 20 | 2 |  |  | 0.89 | 0.86 | 0.87 |  | 15.43 | 9.22 | Cst3 | 25307 | P14841 | 83301921 | -0.27 | 0.194 |  |  | | | | | | | | | | | | | | | | | |
| P19357 | Solute carrier family 2, facilitated glucose transporter member 4 | 6.88 | 4.52 | 2 | 0.81 | 1.06 | 0.77 | 0.87 | 0.87 | 0.13 | 54.86 | 7.3 | Slc2a4 | 25139 | P19357 | 121763 | 0.556 | 0.036 | 1 |  | | | | | | | | | | | | | | | | | |
| Q5M878 | Serum amyloid A protein | 5.89 | 22.31 | 3 |  |  | 0.87 | 0.88 | 0.87 |  | 14.96 | 9.1 | Hps5 | 365245 | Q5M878 | 81889141 | -0.589 | 0.2 |  |  | | | | | | | | | | | | | | | | | |
| D3ZUX5 | Coiled-coil-helix-coiled-coil-helix domain containing 3 (Predicted), isoform CRA_a | 113 | 59.91 | 17 | 0.87 | 0.89 | 0.87 | 0.88 | 0.88 | 0.01 | 26.42 | 8.13 | Chchd3 | 296966 | NP_001100058 | 157817027 | -1.035 | 2.082 | 1 |  | | | | | | | | | | | | | | | | | |
| G3V8L3 | Lamin A, isoform CRA_b | 30.39 | 21.65 | 12 | 0.81 | 0.93 | 0.86 | 0.92 | 0.87 | 0.06 | 74.27 | 6.98 | Lmna | 60374 | NP_001002016 | 612149768 | -0.867 | 0.162 | 2,7,1 |  | | | | | | | | | | | | | | | | | |
| M0R9D5 | Protein Ahnak | 35.57 | 15.29 | 10 | 0.92 | 0.9 | 0.84 | 0.86 | 0.88 | 0.03 | 571.3 | 6.23 | Ahnak | 191572 | NP_001178880 | 300794574 | -0.456 | 0.025 | 1 |  | | | | | | | | | | | | | | | | | |
| P39069 | Adenylate kinase isoenzyme 1 | 31.23 | 36.08 | 6 | 0.94 | 0.83 |  |  | 0.88 |  | 21.57 | 7.9 | Ak1 | 24183 | P39069 | 122065251 | -0.43 | 0.603 | 5,1 |  | | | | | | | | | | | | | | | | | |
| G3V7V6 | All-trans-13,14-dihydroretinol saturase, isoform CRA_b | 16.46 | 11 | 5 | 0.89 | 0.9 | 0.84 | 0.91 | 0.89 | 0.03 | 67.43 | 8.81 | Retsat | 246298 | EDL91048 | 149036430 | -0.036 | 0.089 |  |  | | | | | | | | | | | | | | | | | |
| F1M2D3 | Uncharacterized protein | 118.8 | 49.65 | 2 | 0.76 | 0.83 | 1 | 0.97 | 0.89 | 0.11 | 30.71 | 8.27 | Vdac1 | 83529 | NP_112643.1 | 13786200 | -0.387 | 1.563 |  |  | | | | | | | | | | | | | | | | | |
| P61016 | Cardiac phospholamban | 3.96 | 32.69 | 2 | 0.79 | 0.99 |  |  | 0.89 |  | 6.09 | 8.81 | Pln | 64672 | P61016 | 46577628 | 0.842 | 0.493 |  |  | | | | | | | | | | | | | | | | | |
| Q9R1Z0 | Voltage-dependent anion-selective channel protein 3 | 156.9 | 62.19 | 17 | 0.93 | 0.87 |  |  | 0.9 |  | 30.78 | 8.7 | Vdac3 | 83532 | Q9R1Z0 | 15214186 | -0.296 | 1.754 | 1 |  | | | | | | | | | | | | | | | | | |
| D4A4H3 | Protein Vps13c | 66.55 | 7.67 | 20 | 0.88 | 0.92 |  |  | 0.9 |  | 413.8 | 6.79 | Vps13c | 363087 | EDL84223 | 149028882 | -0.183 | 0.053 |  |  | | | | | | | | | | | | | | | | | |
| Q9WUS0 | GTP:AMP phosphotransferase AK4, mitochondrial | 17.74 | 30.94 | 6 | 0.9 | 1 | 0.84 | 0.87 | 0.9 | 0.07 | 25.19 | 8.02 | Ak4 | 29223 | Q9WUS0 | 6707705 | -0.318 | 0.238 |  |  | | | | | | | | | | | | | | | | | |
| Q63258-3 | Isoform Alpha-7X1C of Integrin alpha-7 | 16.25 | 5.86 | 6 | 0.91 | 0.88 | 0.88 | 0.95 | 0.9 | 0.03 | 117.5 | 5.97 | Itga7 | 81008 | NP_110469 | 57528941 | -0.121 | 0.051 |  |  | | | | | | | | | | | | | | | | | |
| Q99PS8 | Histidine-rich glycoprotein | 31.64 | 19.62 | 8 | 0.91 | 0.89 | 0.84 | 1.01 | 0.91 | 0.07 | 59.01 | 7.84 | Hrg | 171016 | Q99PS8 | 81880331 | -0.921 | 0.186 |  |  | | | | | | | | | | | | | | | | | |
| Q6PCT3 | Tumor protein D54 | 94.97 | 56.82 | 16 | 0.93 | 0.88 | 0.94 | 0.89 | 0.91 | 0.03 | 23.98 | 6.15 | Tpd52l2 | 296480 | Q6PCT3 | 62511141 | -0.59 | 1.376 | 4,12 |  | | | | | | | | | | | | | | | | | |
| G3V9W6 | Aldehyde dehydrogenase | 12.07 | 11.98 | 4 | 0.94 | 0.88 | 1.05 | 0.79 | 0.91 | 0.11 | 54.07 | 7.66 | Aldh3a2 | 65183 | NP_113919 | 166157462 | -0.092 | 0.111 |  |  | | | | | | | | | | | | | | | | | |
| D3ZYY0 | Protein Sdr39u1 | 17.21 | 13.09 | 5 | 0.86 | 1.03 | 0.85 | 0.94 | 0.92 | 0.08 | 31.92 | 8.05 | Sdr39u1 | 361044 | NP_001101848 | 157821511 | 0.036 | 0.188 |  |  | | | | | | | | | | | | | | | | | |
| F1LPV8 | Protein Suclg2 | 39.3 | 33.41 | 12 | 0.91 | 0.92 | 0.95 | 0.9 | 0.91 | 0.02 | 46.61 | 7.71 | Suclg2 | 362404 | NP_001094220 | 189491689 | -0.12 | 0.343 |  |  | | | | | | | | | | | | | | | | | |
| Q6AY58 | B-cell receptor-associated protein 31 | 24.59 | 22.86 | 6 | 1.04 | 0.93 | 0.84 | 0.88 | 0.92 | 0.09 | 27.89 | 8.88 | Bcap31 | 293852 | Q6AY58 | 81891343 | -0.226 | 0.394 | 10,12,1 |  | | | | | | | | | | | | | | | | | |
| P11517 | Hemoglobin subunit beta-2 | 191.1 | 79.59 | 2 | 0.96 | 0.96 | 0.9 | 0.89 | 0.93 | 0.04 | 15.97 | 8.79 | Ptges2 | 100134871,689064 | P11517 | 122529 | -0.003 | 3.569 |  |  | | | | | | | | | | | | | | | | | |
| D4A0T0 | Protein Ndufb10 | 97.96 | 68.75 | 12 | 0.93 | 0.9 | 0.97 | 0.92 | 0.93 | 0.03 | 20.85 | 7.69 | Ndufb10 | 681418 | NP_001102913 | 157822175 | -0.937 | 1.679 |  |  | | | | | | | | | | | | | | | | | |
| D4A4K4 | Protein Vps13c | 63.26 | 6.71 | 20 |  |  | 0.97 | 0.89 | 0.93 |  | 418.4 | 6.84 | Vps13c | 363087 | EDL84223 | 149028882 | -0.19 | 0.055 |  |  | | | | | | | | | | | | | | | | | |
| P57093 | Phytanoyl-CoA dioxygenase, peroxisomal | 15.84 | 18.64 | 5 | 0.85 | 0.91 | 0.95 | 1.01 | 0.93 | 0.07 | 38.56 | 8.48 | Phyh | 114209 | P57093 | 12643482 | -0.459 | 0.078 |  |  | | | | | | | | | | | | | | | | | |
| P12346-2 | Isoform 2 of Serotransferrin | 20.7 | 10.28 | 5 | 0.96 | 0.91 |  |  | 0.93 |  | 54.49 | 7.66 | Tf | 24825 | NP_001013128 | 61556986 | -0.252 | 0.147 |  |  | | | | | | | | | | | | | | | | | |
| F1M8K0 | Protein Dag1 | 6.56 | 3.92 | 3 | 0.91 | 0.73 | 0.94 | 1.16 | 0.82 | 0.18 | 96.65 | 8.44 | Dag1 | 114489 | NP_446149 | 387157894 | -0.357 | 0.031 |  |  | | | | | | | | | | | | | | | | | |
| A9UMV9 | Ndufa7 protein | 84.56 | 77.68 | 13 | 0.9 | 0.99 | 0.94 | 0.92 | 0.94 | 0.04 | 12.49 | 10.48 | Ndufa7 | 299643 | NP_001100242 | 325974480 | -0.662 | 2.562 |  |  | | | | | | | | | | | | | | | | | |
| A2VD12 | Pre-B-cell leukemia transcription factor-interacting protein 1 | 54.06 | 22.3 | 12 | 1 | 1.02 | 0.89 | 0.88 | 0.95 | 0.07 | 80.23 | 5.41 | Pbxip1 | 310644 | A2VD12 | 158706096 | -0.978 | 0.237 |  |  | | | | | | | | | | | | | | | | | |
| Q9Z2L0 | Voltage-dependent anion-selective channel protein 1 | 422.6 | 85.87 | 9 | 0.99 | 0.92 | 0.94 | 0.93 | 0.95 | 0.03 | 30.74 | 8.54 | Vdac1 | 83529 | Q9Z2L0 | 46397782 | -0.423 | 3.872 | 5,1 |  | | | | | | | | | | | | | | | | | |
| P24473 | Glutathione S-transferase kappa 1 | 21.1 | 28.76 | 4 | 1 | 0.96 | 0.91 | 0.93 | 0.95 | 0.04 | 25.48 | 9.07 | Gstk1 | 297029 | P24473 | 3041680 | -0.116 | 0.236 | 1 |  | | | | | | | | | | | | | | | | | |
| D4AD67 | Protein Ktn1 | 7.46 | 2.12 | 2 | 1.03 | 0.87 |  |  | 0.95 | 0 | 145.7 | 6.05 | Ktn1 | 361029 | XP_006221975 | 564317712 | -0.837 | 0.021 |  |  | | | | | | | | | | | | | | | | | |
| P0C5I0 | Cerebral dopamine neurotrophic factor | 7.31 | 14.97 | 3 | 0.96 | 0.92 | 0.93 | 1 | 0.96 | 0.04 | 21.35 | 6.86 | Cdnf | 361276 | P0C5I0 | 158706419 | -0.263 | 0.141 |  |  | | | | | | | | | | | | | | | | | |
| D3ZAN3 | Alpha glucosidase 2 alpha neutral subunit (Predicted) | 18.3 | 11.04 | 7 | 0.93 | 1.03 | 0.89 | 0.98 | 0.98 | 0.06 | 90.51 | 6.2 | Ganab | 293721 | NP_001099804 | 157822919 | -0.342 | 0.088 |  |  | | | | | | | | | | | | | | | | | |
| G3V7K3 | Ceruloplasmin | 8.04 | 4.91 | 4 | 0.94 | 0.91 | 0.93 | 1.07 | 0.96 | 0.08 | 120.6 | 5.64 | Cp | 24268 | NP_036664 | 401461786 | -0.492 | 0.033 |  |  | | | | | | | | | | | | | | | | | |
| P06686 | Sodium/potassium-transporting ATPase subunit alpha-2 | 117.1 | 21.37 | 7 | 0.89 | 0.97 | 0.91 | 1.09 | 0.97 | 0.09 | 112.2 | 5.55 | Atp1a2 | 24212 | P06686 | 114379 | -0.007 | 0.321 |  |  | | | | | | | | | | | | | | | | | |
| D4A249 | Protein Mblac2 | 4.51 | 5.02 | 2 | 0.98 | 0.98 |  |  | 0.98 |  | 31.17 | 6.86 | Mblac2 | 365627 | NP_001102404 | 157821863 | -0.186 | 0.064 |  |  | | | | | | | | | | | | | | | | | |
| F1LR02 | Procollagen, type XVIII, alpha 1, isoform CRA_a | 12.88 | 2.52 | 3 | 0.87 | 1.09 |  |  | 0.98 |  | 134.6 | 6.29 | Col18a1 | 85251 | NP_445941 | 309319796 | -0.628 | 0.03 | 1 |  | | | | | | | | | | | | | | | | | |
| Q62669 | Protein Hbb-b1 | 78.77 | 57.14 | 4 | 0.96 | 0.93 | 1.07 | 0.95 | 0.98 | 0.06 | 16.01 | 8.19 | Hbb-b1 | 361619 | Q62669 | 81890295 | 0.001 | 1.936 |  |  | | | | | | | | | | | | | | | | | |
| B1WC34 | Protein Prkcsh | 12.44 | 10.67 | 5 | 0.93 | 1.05 | 1.08 | 0.88 | 0.98 | 0.1 | 59.18 | 4.48 | Prkcsh | 300445 | NP_001100276 | 157818781 | -0.889 | 0.118 |  |  | | | | | | | | | | | | | | | | | |
| P02091 | Hemoglobin subunit beta-1 | 192.4 | 83.67 | 2 | 1.05 | 0.98 | 0.97 | 0.94 | 1.02 | 0.05 | 15.97 | 8.07 | Hbb | 24440 | P02091 | 122514 | -0.042 | 3.695 |  |  | | | | | | | | | | | | | | | | | |
| P11507-2 | Isoform SERCA2A of Sarcoplasmic/endoplasmic reticulum calcium ATPase 2 | 934.6 | 47.94 | 63 | 0.98 | 0.98 | 1 | 1 | 0.99 | 0.01 | 109.6 | 5.36 | Atp2a2 | 29693 | NP_001104293 | 161016776 | 0.092 | 2.582 |  |  | | | | | | | | | | | | | | | | | |
| F1LN18 | Hypoxia up-regulated protein 1 | 27.27 | 11.81 | 10 | 0.97 | 1.07 | 0.93 | 1.01 | 1 | 0.06 | 111.2 | 5.19 | Hyou1 | 192235 | NP_001029200 | 77404375 | -0.567 | 0.108 |  |  | | | | | | | | | | | | | | | | | |
| Q5M7T6 | ATPase, H+ transporting, lysosomal 38kDa, V0 subunit d1 | 4.55 | 5.41 | 2 |  |  | 0.95 | 1.05 | 1 |  | 40.28 | 5 | Atp6v0d1 | 291969 | Q5M7T6 | 81883040 | -0.093 | 0.05 | 7,1 |  | | | | | | | | | | | | | | | | | |
| Q7TP42 | Ab2-292 | 7.76 | 4.96 | 3 | 0.95 | 1 | 1.14 | 0.92 | 1 | 0.1 | 67.89 | 7.58 | Sec62 | 294912 | Q7TP42 | 81865307 | -0.929 | 0.044 |  |  | | | | | | | | | | | | | | | | | |
| P10960 | Sulfated glycoprotein 1 | 25.54 | 7.4 | 5 | 1.03 | 1.07 | 1.01 | 0.91 | 1 | 0.07 | 61.08 | 5.25 | Psap | 25524 | P10960 | 134219 | -0.034 | 0.164 |  |  | | | | | | | | | | | | | | | | | |
| Q80W89 | NADH dehydrogenase [ubiquinone] 1 alpha subcomplex subunit 11 | 23.46 | 38.3 | 4 | 1.15 | 1.16 | 0.79 | 0.92 | 1.01 | 0.18 | 14.84 | 8.09 | Ndufa11 | 301123 | Q80W89 | 52000746 | 0.157 | 0.472 | 1 |  | | | | | | | | | | | | | | | | | |
| D3ZJW3 | PTPRF interacting protein, binding protein 1 (Liprin beta 1) (Predicted) | 8.44 | 5.41 | 4 | 1.11 | 0.93 | 1.06 | 0.95 | 1.02 | 0.09 | 101.2 | 5.69 | Ppfibp1 | 312855 | NP_001101366 | 157824075 | -0.676 | 0.04 |  |  | | | | | | | | | | | | | | | | | |
| D4ACG2 | IlvB (Bacterial acetolactate synthase)-like (Predicted), isoform CRA_c | 8.18 | 6.55 | 3 |  |  | 0.94 | 1.09 | 1.01 |  | 72.22 | 8.28 | Ilvbl | 362843 | NP_001102208 | 157823815 | 0.204 | 0.042 |  |  | | | | | | | | | | | | | | | | | |
| P01946 | Hemoglobin subunit alpha-1/2 | 105.5 | 64.08 | 9 | 1.04 | 1 | 1.03 | 0.99 | 1.02 | 0.03 | 15.32 | 7.97 | Hba1 | 25632,360504 | P01946 | 122477 | -0.115 | 2.285 | 1 |  | | | | | | | | | | | | | | | | | |
| G3V8U8 | Branched-chain-amino-acid aminotransferase | 7.99 | 13.23 | 4 | 0.95 | 1.02 | 0.99 | 1.1 | 1.02 | 0.07 | 44.2 | 8.16 | Bcat2 | 64203 | EDM07329 | 149055898 | -0.143 | 0.09 |  |  | | | | | | | | | | | | | | | | | |
| D3ZKE6 | Sarcolemma associated protein (Predicted) | 164.6 | 30.88 | 29 | 1.01 | 0.95 | 1.06 | 1.06 | 1.02 | 0.05 | 90.5 | 5.2 | Slmap | 290533 | NP_001099530 | 157819677 | -0.802 | 0.729 |  |  | | | | | | | | | | | | | | | | | |
| F1M944 | Calsequestrin | 286.6 | 51.57 | 26 | 1.04 | 0.96 | 1.1 | 1 | 1.02 | 0.06 | 47.87 | 4.31 | Casq2 | 29209 | AAH72547 | 47940716 | -0.609 | 1.964 | 1 |  | | | | | | | | | | | | | | | | | |
| Q6AXS5-2 | Isoform 2 of Plasminogen activator inhibitor 1 RNA-binding protein | 12.95 | 12.76 | 4 | 1.01 | 1.04 |  |  | 1.03 |  | 42.96 | 8.44 | Serbp1 | 246303 | XP_006236678 | 564347159 | -1.281 | 0.14 |  |  | | | | | | | | | | | | | | | | | |
| P70490 | Lactadherin | 12.59 | 12.65 | 4 | 1.1 | 1.07 | 0.97 | 1.05 | 1.09 | 0.06 | 47.38 | 7.09 | Mfge8 | 25277 | P70490 | 2494287 | -0.258 | 0.148 |  |  | | | | | | | | | | | | | | | | | |
| Q499S6 | Cathepsin F | 10.14 | 9.09 | 3 | 1.12 | 1.07 | 0.96 | 1.04 | 1.05 | 0.07 | 51.8 | 6.21 | Ctsf | 361704 | Q499S6 | 123781645 | -0.24 | 0.058 |  |  | | | | | | | | | | | | | | | | | |
| P23711 | Heme oxygenase 2 | 11.76 | 11.43 | 2 | 1.12 | 0.98 |  |  | 1.05 |  | 35.74 | 5.49 | Hmox2 | 79239 | P23711 | 123449 | -0.545 | 0.084 |  |  | | | | | | | | | | | | | | | | | |
| P80067 | Dipeptidyl peptidase 1 | 4.14 | 2.6 | 2 |  |  | 1 | 1.13 | 1.06 |  | 52.2 | 6.89 | Ctsc | 25423 | P80067 | 114152780 | -0.26 | 0.038 |  |  | | | | | | | | | | | | | | | | | |
| P20761 | Ig gamma-2B chain C region | 5.78 | 7.21 | 2 |  |  | 1.02 | 1.12 | 1.07 |  | 36.47 | 7.64 | Igh-1a | 102546876 | P20761 | 121055 | -0.35 | 0.082 |  |  | | | | | | | | | | | | | | | | | |
| M0R3V4 | Protein LOC501282 | 14.37 | 24.85 | 4 | 1.07 | 1.08 | 1.18 | 0.98 | 1.08 | 0.08 | 17.84 | 6.16 | LOC501282 | 501282 | XP_008756141 | 672023602 | -0.138 | 0.336 |  |  | | | | | | | | | | | | | | | | | |
| G3V7J0 | Aldehyde dehydrogenase family 6, subfamily A1, isoform CRA_b | 108.3 | 49.35 | 18 | 1.1 | 1.08 | 1.11 | 1.02 | 1.08 | 0.04 | 57.71 | 8.18 | Aldh6a1 | 81708 | NP_112319 | 145651820 | -0.041 | 0.554 |  |  | | | | | | | | | | | | | | | | | |
| P53534 | Glycogen phosphorylase, brain form (Fragment) | 15.98 | 6.68 | 3 | 1.01 | 1.01 | 1.21 | 1.1 | 1.01 | 0.09 | 96.11 | 6.68 | Pygb | 25739 | P53534 | 1730559 | -0.322 | 0.073 | 1 |  | | | | | | | | | | | | | | | | | |
| Q5RK27 | Solute carrier family 12 member 7 | 9.9 | 4.16 | 4 | 1.08 | 1.1 | 1.05 | 1.11 | 1.09 | 0.02 | 119.3 | 6.58 | Slc12a7 | 308069 | Q5RK27 | 156633625 | 0.123 | 0.034 | 1 |  | | | | | | | | | | | | | | | | | |
| Q6WN19 | Protein Rtn2 | 17.2 | 4.05 | 2 | 1.07 | 1 | 1.1 | 1.19 | 1.09 | 0.08 | 50.94 | 4.97 | Rtn2 | 308410 | Q6WN19 | 81864458 | -0.283 | 0.098 |  |  | | | | | | | | | | | | | | | | | |
| D3ZE15 | Protein Ndufa13 | 82.43 | 72.22 | 14 | 1.06 | 1.09 | 1.1 | 1.12 | 1.09 | 0.02 | 16.77 | 9.48 | Ndufa13 | 100911483 |  |  | -0.437 | 1.73 |  |  | | | | | | | | | | | | | | | | | |
| Q6RJR6-2 | Isoform 2 of Reticulon-3 | 2.27 | 7.17 | 2 | 1.05 | 1.14 |  |  | 1.09 |  | 25.41 | 8.5 | Rtn3 | 140945 | NP_543185 | 57977297 | -0.409 | 0.079 |  |  | | | | | | | | | | | | | | | | | |
| D3ZV37 | Protein Cisd3 | 11.8 | 19.62 | 4 | 1.08 | 1.01 | 1.15 | 1.14 | 1.09 | 0.07 | 18.06 | 9.77 | Cisd3 | 287661 | NP_001099305 | 157786678 | -0.084 | 0.332 |  |  | | | | | | | | | | | | | | | | | |
| E9PSR7 | Uncharacterized protein (Fragment) | 8.47 | 3.01 | 3 |  |  | 1.07 | 1.12 | 1.1 |  | 118 | 8.4 |  | 309243 | AAP92652 | 33086680 | -0.385 | 0.025 |  |  | | | | | | | | | | | | | | | | | |
| P02770 | Serum albumin | 308.6 | 71.38 | 40 | 1.16 | 1.07 | 1.17 | 1.01 | 1.11 | 0.07 | 68.69 | 6.48 | Alb | 24186 | P02770 | 124028612 | -0.38 | 1.601 |  |  | | | | | | | | | | | | | | | | | |
| P13221 | Aspartate aminotransferase, cytoplasmic | 14.44 | 14.29 | 4 | 1.11 | 1.08 | 1.08 | 1.19 | 1.11 | 0.05 | 46.4 | 7.21 | Got1 | 24401 | P13221 | 122065118 | -0.305 | 0.086 | 1 |  | | | | | | | | | | | | | | | | | |
| D4A4B0 | Uncharacterized protein (Fragment) | 4.45 | 0.35 | 2 |  |  | 1.1 | 1.15 | 1.12 | 0 | 583.9 | 5.62 |  |  |  | 672046076 | -0.608 | 0.003 |  |  | | | | | | | | | | | | | | | | | |
| Q8VBU2-2 | Isoform 2 of Protein NDRG2 | 115.1 | 52.94 | 14 | 1.13 | 1.12 | 1.17 | 1.09 | 1.13 | 0.03 | 39.25 | 5.49 | Ndrg2 | 171114 | Q8VBU2 | 81867103 | -0.272 | 0.866 |  |  | | | | | | | | | | | | | | | | | |
| P06685 | Sodium/potassium-transporting ATPase subunit alpha-1 | 296.9 | 42.62 | 31 | 1.18 | 1.08 | 1.15 | 1.1 | 1.13 | 0.04 | 113 | 5.45 | Atp1a1 | 24211 | P06685 | 114376 | 0.002 | 0.867 |  |  | | | | | | | | | | | | | | | | | |
| D3ZN21 | Protein RGD1309586 | 28.78 | 11.08 | 2 |  |  | 1.12 | 1.14 | 1.13 |  | 72.95 | 7.03 | RGD1309586 | 364073 | NP_001102328 | 157819755 | -0.619 | 0.137 |  |  | | | | | | | | | | | | | | | | | |
| F1M013 | Protein LOC100910109 (Fragment) | 28.72 | 30.57 | 8 | 1.16 | 1.16 | 1.08 | 1.11 | 1.13 | 0.04 | 29.85 | 10.61 | LOC100910109 | 296596 | NP_001107863 | 167466288 | -0.556 | 0.335 |  |  | | | | | | | | | | | | | | | | | |
| P08010 | Glutathione S-transferase Mu 2 | 8.77 | 15.14 | 2 | 1.01 | 1.25 |  |  | 1.13 |  | 25.69 | 7.39 | Gstm2 | 24424 | P08010 | 121719 | -0.502 | 0.117 |  |  | | | | | | | | | | | | | | | | | |
| P07340 | Sodium/potassium-transporting ATPase subunit beta-1 | 76.94 | 39.14 | 13 | 1.17 | 1.09 | 1.09 | 1.18 | 1.13 | 0.05 | 35.18 | 8.65 | Atp1b1 | 25650 | P07340 | 114395 | -0.548 | 0.966 |  |  | | | | | | | | | | | | | | | | | |
| D3ZKC6 | Protein Vps13d | 20.79 | 1.92 | 6 | 1.23 | 1.28 | 0.93 | 1.11 | 1.14 | 0.15 | 488.7 | 6.54 | Vps13d | 313825 | XP_006239411 | 564353981 | -0.296 | 0.012 |  |  | | | | | | | | | | | | | | | | | |
| D3ZJE2 | Uncharacterized protein | 10.54 | 29.7 | 4 |  |  | 1.09 | 1.2 | 1.15 |  | 17.84 | 9.26 | Rpl12-ps1 | 499782 | NP_001102668 | 157822227 | -0.401 | 0.28 |  |  | | | | | | | | | | | | | | | | | |
| B2RYX0 | Naca protein | 10.6 | 13.02 | 2 |  |  | 1.14 | 1.17 | 1.16 |  | 23.37 | 4.56 | Naca | 288770 | NP_001099409 | 157786942 | -0.655 | 0.128 |  |  | | | | | | | | | | | | | | | | | |
| P20760 | Ig gamma-2A chain C region | 6.14 | 10.56 | 3 | 1.15 | 1.18 | 1.21 | 1.1 | 1.16 | 0.05 | 35.16 | 7.68 | Igg-2a | 679045 | P20760 | 121052 | -0.371 | 0.085 |  |  | | | | | | | | | | | | | | | | | |
| F1M5H4 | Uncharacterized protein | 2.95 | 12.93 | 2 | 1.01 | 1.31 |  |  | 1.16 |  | 16.5 | 10.73 |  | 293418 | NP_001099760 | 157820727 | -0.647 | 0.182 |  |  | | | | | | | | | | | | | | | | | |
| Q4KLZ0 | Protein Vnn1 | 22.78 | 14.06 | 6 | 1.08 | 1.15 | 1.16 | 1.27 | 1.11 | 0.08 | 56.98 | 5.55 | Vnn1 | 29142 | Q4KLZ0 | 123795700 | 0.054 | 0.14 |  |  | | | | | | | | | | | | | | | | | |
| P04762 | Catalase | 57.64 | 35.67 | 14 | 1.12 | 1.19 | 1.12 | 1.23 | 1.17 | 0.06 | 59.72 | 7.5 | Cat | 24248 | P04762 | 115707 | -0.639 | 0.301 | 7 |  | | | | | | | | | | | | | | | | | |
| Q6PDW8 | Glutathione peroxidase | 21.28 | 54.48 | 6 | 1.19 | 1.19 | 1.22 | 1.09 | 1.17 | 0.06 | 16.48 | 7.25 | Gpx1 | 24404 | Q6PDW8 | 81892539 | -0.515 | 0.607 | 1 |  | | | | | | | | | | | | | | | | | |
| P04797 | Glyceraldehyde-3-phosphate dehydrogenase | 118.6 | 48.35 | 12 |  |  | 1.22 | 1.14 | 1.18 |  | 35.81 | 8.03 | Gapdh | 24383 | P04797 | 122065190 | -0.089 | 0.838 | 5,7,8,9,10,15,1 |  | | | | | | | | | | | | | | | | | |
| P05708 | Hexokinase-1 | 112.3 | 31.81 | 30 | 1.1 | 1.26 | 0.92 | 1.44 | 1.18 | 0.22 | 102.3 | 6.71 | Hk1 | 25058 | P05708 | 6226638 | -0.198 | 0.42 |  |  | | | | | | | | | | | | | | | | | |
| P26453-2 | Isoform 2 of Basigin | 70.81 | 28.68 | 9 | 1.2 | 1.21 | 1.16 | 1.16 | 1.18 | 0.02 | 29.57 | 5.27 | Bsg | 25246 | NP_001103352 | 158081773 | -0.305 | 0.879 |  |  | | | | | | | | | | | | | | | | | |
| M0R776 | Protein Mrps36 | 30.39 | 62.14 | 4 | 1.12 | 1.14 | 1.25 | 1.22 | 1.18 | 0.07 | 11.41 | 10.35 | Mrps36 | 294696 | NP_001178534 | 300797955 | -0.675 | 0.701 |  |  | | | | | | | | | | | | | | | | | |
| M0RBJ7 | Complement C3 | 20.73 | 3.31 | 4 | 1.06 | 1.17 | 1.14 | 1.37 | 1.11 | 0.13 | 186.1 | 6.47 | C3 | 24232 | NP_058690.2 | 158138561 | -0.285 | 0.027 |  |  | | | | | | | | | | | | | | | | | |
| P07632 | Superoxide dismutase [Cu-Zn] | 22.66 | 38.96 | 5 | 1.17 | 1.27 | 1.2 | 1.11 | 1.19 | 0.07 | 15.9 | 6.35 | Sod1 | 24786 | P07632 | 134625 | -0.404 | 0.44 |  |  | | | | | | | | | | | | | | | | | |
| P48500 | Triosephosphate isomerase | 41.29 | 37.35 | 9 | 1.16 | 1.2 | 1.23 | 1.19 | 1.19 | 0.03 | 26.83 | 7.24 | Tpi1 | 24849 | P48500 | 124056485 | -0.1 | 0.485 |  |  | | | | | | | | | | | | | | | | | |
| P15429 | Beta-enolase | 21.8 | 14.98 | 3 | 1.19 | 1.21 | 1.18 | 1.2 | 1.2 | 0.01 | 46.98 | 7.44 | Eno3 | 25438 | P15429 | 122065177 | -0.196 | 0.128 |  |  | | | | | | | | | | | | | | | | | |
| G3V6U3 | Asparagine-linked glycosylation 2 homolog (Yeast, alpha-1,3-mannosyltransferase), isoform CRA_a | 8.2 | 7.71 | 2 | 1.1 | 1.3 |  |  | 1.2 |  | 47.29 | 7.97 | Alg2 | 313231 | NP_001094180 | 213511844 | -0.058 | 0.042 |  |  | | | | | | | | | | | | | | | | | |
| P00564 | Creatine kinase M-type | 71.03 | 45.93 | 12 | 1.21 | 1.24 | 1.21 | 1.2 | 1.22 | 0.02 | 43.02 | 7.06 | Ckm | 24265 | P00564 | 124056470 | -0.596 | 0.488 | 1 |  | | | | | | | | | | | | | | | | | |
| D4AAB5 | Protein Pm20d2 | 10.28 | 4.18 | 3 | 1.3 | 1.13 | 1.44 | 1 | 1.22 | 0.19 | 46.65 | 5.57 | Pm20d2 | 313130 | NP_001101392 | 298493325 | -0.128 | 0.129 |  |  | | | | | | | | | | | | | | | | | |
| M0R5J4 | Uncharacterized protein | 41.97 | 24.88 | 6 | 1.23 | 1.23 | 1.18 | 1.23 | 1.23 | 0.03 | 47.05 | 6.37 |  | 24333 | NP_036686 | 158186649 | -0.177 | 0.34 |  |  | | | | | | | | | | | | | | | | | |
| D3ZU04 | Uncharacterized protein | 4.4 | 18.55 | 3 | 1.24 | 1.55 | 0.99 | 1.15 | 1.24 | 0.24 | 14.3 | 10.43 |  | 64298 | NP_071951 | 11968078 | -0.836 | 0.28 |  |  | | | | | | | | | | | | | | | | | |
| D3ZJ63 | Protein LOC312502 (Fragment) | 13.63 | 9.18 | 5 | 1.22 | 1.23 | 1.27 | 1.23 | 1.24 | 0.02 | 69.59 | 9.26 | LOC312502 | 312502 | XP_006225034 | 564303964 | -0.666 | 0.072 |  |  | | | | | | | | | | | | | | | | | |
| P24268 | Cathepsin D | 158.5 | 42.26 | 15 | 1.27 | 1.25 | 1.26 | 1.21 | 1.25 | 0.03 | 44.65 | 7.09 | Ctsd | 171293 | P24268 | 115720 | 0.008 | 1.12 |  |  | | | | | | | | | | | | | | | | | |
| P19804 | Nucleoside diphosphate kinase B | 38.96 | 50.66 | 7 | 1.24 | 1.38 | 1.17 | 1.21 | 1.25 | 0.09 | 17.27 | 7.44 | Nme2 | 83782 | P19804 | 127984 | -0.27 | 0.637 |  |  | | | | | | | | | | | | | | | | | |
| P00787 | Cathepsin B | 27.8 | 14.16 | 4 | 1.28 | 1.24 | 1.32 | 1.31 | 1.29 | 0.04 | 37.45 | 5.6 | Ctsb | 64529 | P00787 | 1705630 | -0.355 | 0.374 |  |  | | | | | | | | | | | | | | | | | |
| F1M4S5 | Uncharacterized protein (Fragment) | 6.2 | 26.42 | 4 | 1.34 | 1.25 |  |  | 1.29 |  | 12.27 | 10.4 | RGD1563431 | 103692456 | XP_008762216 | 672027565 | -1.153 | 0.407 |  |  | | | | | | | | | | | | | | | | | |
| P16290 | Phosphoglycerate mutase 2 | 18.88 | 16.6 | 4 |  |  | 1.26 | 1.34 | 1.3 |  | 28.74 | 8.72 | Pgam2 | 24959 | P16290 | 130354 | -0.543 | 0.209 | 1 |  | | | | | | | | | | | | | | | | | |
| Q3KRE2 | Methyltransferase like 7A | 168.2 | 49.59 | 12 | 1.32 | 1.32 | 1.31 | 1.31 | 1.31 | 0.01 | 28.12 | 8.46 | Mettl7a | 315306 | Q3KRE2 | 123780644 | 0.146 | 1.742 | 6,9,1 |  | | | | | | | | | | | | | | | | | |
| A8WCF8 | Tumor protein p63-regulated gene 1-like protein | 228.4 | 74.44 | 17 | 1.36 | 1.3 | 1.39 | 1.31 | 1.34 | 0.04 | 29.83 | 7.37 | Tprg1l | 687090 | A8WCF8 | 205830336 | -0.239 | 2.246 | 7，10 |  | | | | | | | | | | | | | | | | | |
| F1LM30 | Uncharacterized protein (Fragment) | 58.84 | 29.36 | 13 | 1.43 | 1.37 | 1.31 | 1.34 | 1.37 | 0.05 | 51.71 | 6.39 |  | 299357 | AAI05826 | 77748244 | -0.203 | 0.522 |  |  | | | | | | | | | | | | | | | | | |
| P16617 | Phosphoglycerate kinase 1 | 25.91 | 20.62 | 8 | 1.29 | 1.57 | 1.3 | 1.31 | 1.37 | 0.14 | 44.51 | 7.9 | Pgk1 | 24644 | P16617 | 124106305 | -0.083 | 0.27 |  |  | | | | | | | | | | | | | | | | | |
| A1L134 | Ancient ubiquitous protein 1 | 76.46 | 27.8 | 11 | 1.38 | 1.28 | 1.39 | 1.5 | 1.39 | 0.09 | 46.17 | 8.4 | Aup1 | 680423 | A1L134 | 221222583 | -0.099 | 0.628 | 2 |  | | | | | | | | | | | | | | | | | |
| P04642 | L-lactate dehydrogenase A chain | 21.23 | 18.07 | 4 | 1.53 | 1.48 | 1.45 | 1.48 | 1.49 | 0.03 | 36.43 | 8.27 | Ldha | 24533 | P04642 | 126051 | 0.064 | 0.247 |  |  | | | | | | | | | | | | | | | | | |
| P05065 | Fructose-bisphosphate aldolase A | 102.7 | 62.64 | 20 | 1.56 | 1.5 | 1.42 | 1.4 | 1.53 | 0.07 | 39.33 | 8.09 | Aldoa | 24189 | P05065 | 113609 | -0.273 | 0.89 |  |  | | | | | | | | | | | | | | | | | |
| P42123 | L-lactate dehydrogenase B chain | 96.41 | 43.11 | 15 | 1.52 | 1.56 | 1.5 | 1.44 | 1.51 | 0.05 | 36.59 | 6.05 | Ldhb | 24534 | P42123 | 1170739 | 0.039 | 0.984 |  |  | | | | | | | | | | | | | | | | | |
| P20059 | Hemopexin | 7.54 | 6.52 | 3 | 1.63 | 1.64 | 1.57 | 1.71 | 1.64 | 0.06 | 51.32 | 7.65 | Hpx | 58917 | P20059 | 122065203 | -0.408 | 0.058 |  |  | | | | | | | | | | | | | | | | | |
| Q6AY30 | Saccharopine dehydrogenase-like oxidoreductase | 393.5 | 48.48 | 28 | 1.66 | 1.54 | 1.75 | 1.6 | 1.64 | 0.09 | 47.06 | 8.84 | Sccpdh | 305021 | Q6AY30 | 73919297 | -0.038 | 2.699 | 15,17 |  | | | | | | | | | | | | | | | | | |
| Q6UK00 | Promethin | 37.92 | 22.36 | 7 | 1.7 | 1.58 | 1.81 | 1.6 | 1.7 | 0.1 | 17.57 | 6.51 | Tmem159 | 378467 | Q6UK00 | 81864301 | 0.755 | 0.968 |  |  | | | | | | | | | | | | | | | | | |
| G3V9Z3 | Amine oxidase [flavin-containing] A | 217.5 | 47.34 | 29 | 1.77 | 1.7 | 1.9 | 1.74 | 1.77 | 0.09 | 59.48 | 7.93 | Maoa | 29253 | NP_387502 | 270288740 | -0.213 | 1.261 |  |  | | | | | | | | | | | | | | | | | |
| E9PTT7 | Protein Dhrsx | 20.49 | 21.35 | 3 | 1.99 | 1.95 | 1.83 | 2.04 | 1.95 | 0.09 | 19.26 | 10.49 | Dhrsx | 288525 | AAI67053.1 | 187469315 | 0.084 | 0.363 |  |  | | | | | | | | | | | | | | | | | |
| **cell skeleton^1^** | | | | | | | | | | | | | | | | | | |  | |  |  |  |  |  |  |  |  |  |  |  |  |  |  |  |  |  |
| E9PT87 | Myosin light chain kinase 3 | 9.8 | 5.73 | 4 | 0.71 | 0.63 | 0.68 | 0.72 | 0.69 | 0.04 | 85.49 | 5.5 | Mylk3 | 291926 | E9PT87 | 408407655 | -0.342 | 0.047 |  |  | | | | | | | | | | | | | | | | | |
| Q08163 | Adenylyl cyclase-associated protein 1 | 5.66 | 2.95 | 2 | 0.75 | 0.74 |  |  | 0.74 |  | 51.56 | 7.52 | Cap1 | 64185 | Q08163 | 124012084 | -0.352 | 0.039 |  |  | | | | | | | | | | | | | | | | | |
| G3V8C3 | Vimentin | 82.39 | 42.27 | 15 | 0.79 | 0.77 | 0.68 | 0.74 | 0.75 | 0.05 | 53.67 | 5.12 | Vim | 81818 | EDL78721 | 149021114 | -0.839 | 0.578 | 7,12,14 |  | | | | | | | | | | | | | | | | | |
| Q78P75 | Dynein light chain 2, cytoplasmic | 13.21 | 50.56 | 3 | 0.76 | 0.75 | 0.84 | 0.76 | 0.78 | 0.04 | 10.34 | 7.37 | Dynll2 | 140734 | Q78P75 | 56748619 | -0.456 | 0.677 | 1 |  | | | | | | | | | | | | | | | | | |
| P85972 | Vinculin | 35.36 | 10.79 | 10 | 0.69 | 0.79 | 0.81 | 0.88 | 0.79 | 0.08 | 116.5 | 6.09 | Vcl | 305679 | P85972 | 205830826 | -0.414 | 0.103 |  |  | | | | | | | | | | | | | | | | | |
| G3V6S0 | Protein Sptbn1 | 12.42 | 2.38 | 5 | 0.97 | 0.77 | 0.71 | 0.74 | 0.8 | 0.12 | 273.3 | 5.72 | Sptbn1 | 305614 | NP_001013148 | 61557085 | -0.748 | 0.022 |  |  | | | | | | | | | | | | | | | | | |
| P85108 | Tubulin beta-2A chain | 265.8 | 71.69 | 5 | 0.73 | 0.81 | 0.87 | 0.86 | 0.77 | 0.06 | 49.87 | 4.89 | Tubb2a | 498736 | P85108 | 144587401 | -0.408 | 1.624 | 7,8,12,15,16,1 |  | | | | | | | | | | | | | | | | | |
| B5DFL0 | Protein Snta1 | 15.18 | 9.62 | 3 | 0.79 | 0.84 | 0.79 | 0.85 | 0.82 | 0.03 | 53.33 | 6.8 | Snta1 | 362242 | NP_001094371 | 201023317 | -0.218 | 0.075 |  |  | | | | | | | | | | | | | | | | | |
| P16086 | Spectrin alpha chain, non-erythrocytic 1 | 26.22 | 4.41 | 7 | 0.77 | 0.87 | 0.85 | 0.8 | 0.82 | 0.05 | 284.5 | 5.33 | Sptan1 | 64159 | P16086 | 17380501 | -0.789 | 0.028 | 1 |  | | | | | | | | | | | | | | | | | |
| P68370 | Tubulin alpha-1A chain | 177.8 | 54.99 | 7 | 0.86 | 0.74 | 0.92 | 0.82 | 0.84 | 0.07 | 50.1 | 5.06 | Tuba1a | 64158 | P68370 | 55977470 | -0.229 | 1.098 |  |  | | | | | | | | | | | | | | | | | |
| F1LP60 | Moesin (Fragment) | 7.5 | 5.03 | 3 | 0.92 | 0.82 | 0.91 | 0.71 | 0.84 | 0.1 | 67.61 | 6.47 | Msn | 81521 | EDL95973 | 149042266 | -0.994 | 0.044 | 1 |  | | | | | | | | | | | | | | | | | |
| D3ZWC6 | Protein Sntb1 | 23.35 | 12.43 | 5 | 0.82 | 0.91 | 0.83 | 0.91 | 0.87 | 0.05 | 58.26 | 8.31 | Sntb1 | 299940 | NP_001124014 | 194474040 | -0.268 | 0.12 |  |  | | | | | | | | | | | | | | | | | |
| Q63355 | Unconventional myosin-Ic | 5.59 | 2.68 | 2 |  |  | 0.86 | 0.91 | 0.89 |  | 119.7 | 9.39 | Myo1c | 65261 | Q63355 | 226723126 | -0.41 | 0.017 |  |  | | | | | | | | | | | | | | | | | |
| Q505J9 | ATPase family AAA domain-containing protein 1 | 54.01 | 38.5 | 11 | 0.89 | 0.91 | 0.89 | 0.85 | 0.89 | 0.03 | 40.69 | 6.9 | Atad1 | 309532 | Q505J9 | 81908923 | -0.246 | 0.467 |  |  | | | | | | | | | | | | | | | | | |
| F1LRT9 | Cytoplasmic dynein 1 heavy chain 1 | 13.69 | 1.16 | 4 | 0.87 | 0.97 | 0.88 | 0.83 | 0.89 | 0.06 | 531.4 | 6.48 | Dync1h1 | 29489 | NP_062099 | 148491097 | -0.336 | 0.009 | 14,1 |  | | | | | | | | | | | | | | | | | |
| P56741 | Myosin-binding protein C, cardiac-type | 11.75 | 3.85 | 4 | 0.87 | 0.76 | 0.91 | 1.04 | 0.89 | 0.12 | 140.7 | 6.54 | Mybpc3 | 295929 | P56741 | 322510050 | -0.422 | 0.028 |  |  | | | | | | | | | | | | | | | | | |
| Q5XID6 | Protein Sgcg | 10.28 | 9.59 | 2 |  |  | 1.11 | 0.7 | 0.9 |  | 32.16 | 5.91 | Sgcg | 305941 | Q5XID6 | 81883752 | -0.068 | 0.093 |  |  | | | | | | | | | | | | | | | | | |
| Q6P0K8 | Junction plakoglobin | 15.35 | 8.72 | 4 | 0.87 | 0.96 | 0.81 | 0.97 | 0.9 | 0.08 | 81.75 | 6.14 | Jup | 81679 | Q6P0K8 | 81885083 | -0.162 | 0.061 |  |  | | | | | | | | | | | | | | | | | |
| Q2PQA9 | Kinesin-1 heavy chain | 4.98 | 3.63 | 4 | 0.91 | 0.88 | 0.94 | 0.89 | 0.91 | 0.03 | 109.5 | 6.44 | Kif5b | 117550 | Q2PQA9 | 109892476 | -0.835 | 0.037 |  |  | | | | | | | | | | | | | | | | | |
| F7EUB6 | Fibrinogen alpha chain | 11.84 | 9.16 | 3 | 0.94 | 0.87 | 0.96 | 0.88 | 0.91 | 0.04 | 60.09 | 7.4 | Fga | 361969 | NP_434684 | 144922622 | -0.845 | 0.067 | 14 |  | | | | | | | | | | | | | | | | | |
| P60711 | Actin, cytoplasmic 1 | 306.9 | 51.73 | 3 | 0.92 | 0.88 | 0.98 | 0.95 | 0.93 | 0.05 | 41.71 | 5.48 | Actb | 81822 | P60711 | 46397316 | -0.2 | 2.565 | 4,5,7,8,15,16,1 |  | | | | | | | | | | | | | | | | | |
| Q5XIF6 | Tubulin alpha-4A chain | 167.6 | 55.8 | 7 | 1.03 | 0.91 | 0.97 | 0.87 | 0.94 | 0.07 | 49.89 | 5.06 | Tuba4a | 316531 | Q5XIF6 | 81889864 | -0.252 | 1.082 | 7,8,12,15,16,1 |  | | | | | | | | | | | | | | | | | |
| P69897 | Tubulin beta-5 chain | 267.8 | 75 | 4 | 1.07 | 0.81 | 1.07 | 0.84 | 0.95 | 0.14 | 49.64 | 4.89 | Tubb5 | 29214 | P69897 | 56754676 | -0.348 | 1.733 | 1 |  | | | | | | | | | | | | | | | | | |
| P02680-2 | Isoform Gamma-A of Fibrinogen gamma chain | 6.17 | 7.78 | 2 | 0.97 | 1.09 | 0.84 | 0.9 | 0.95 | 0.1 | 49.62 | 6.25 | Fgg | 24367 | XP_006232587 | 564336900 | -0.613 | 0.04 |  |  | | | | | | | | | | | | | | | | | |
| Q6P725 | Desmin | 112.1 | 54.8 | 20 |  |  | 0.91 | 1 | 0.96 |  | 53.39 | 5.27 | Des | 64362 | Q6P725 | 81892277 | -0.705 | 0.749 | 1 |  | | | | | | | | | | | | | | | | | |
| P49134 | Integrin beta-1 | 15.46 | 6.13 | 4 | 0.93 | 0.99 | 0.92 | 1.03 | 0.97 | 0.05 | 88.44 | 6.07 | Itgb1 | 24511 | P49134 | 1352494 | -0.374 | 0.057 |  |  | | | | | | | | | | | | | | | | | |
| P48675 | Desmin | 110.3 | 55.44 | 18 | 1 | 0.97 |  |  | 0.98 |  | 53.42 | 5.27 | Des | 64362 | P48675 | 1352241 | -0.709 | 0.73 | 1 |  | | | | | | | | | | | | | | | | | |
| G3V885 | Myosin-6 | 1089 | 58.84 | 30 | 1.01 | 0.98 | 0.98 | 0.99 | 0.99 | 0.01 | 223.4 | 5.73 | Myh6 | 29556 | NP_058935.2 | 186659510 | -0.81 | 1.724 | 1,8 |  | | | | | | | | | | | | | | | | | |
| Q64119 | Myosin light polypeptide 6 | 21.65 | 40.4 | 4 | 0.96 | 0.99 | 1.17 | 1.01 | 1.03 | 0.09 | 16.96 | 4.55 | Myl6 | 362816 | Q64119 | 2842665 | -0.406 | 0.354 | 1 |  | | | | | | | | | | | | | | | | | |
| P13832 | Myosin regulatory light chain RLC-A | 8.95 | 21.51 | 3 | 1.02 | 0.97 | 1.11 | 1.11 | 1.05 | 0.07 | 19.88 | 4.81 | Rlc-a | 501203 | P13832 | 127170 | -0.793 | 0.151 | 1 |  | | | | | | | | | | | | | | | | | |
| P68035 | Actin, alpha cardiac muscle 1 | 459.2 | 77.19 | 12 | 1.05 | 1.09 | 1.05 | 1.03 | 1.05 | 0.02 | 41.99 | 5.39 | Actc1 | 29275 | P68035 | 54036667 | -0.228 | 3.739 | 2,4,5,7,8,16,1 |  | | | | | | | | | | | | | | | | | |
| F1LMI8 | Troponin T, cardiac muscle | 124 | 40.48 | 16 | 1.05 | 1.07 | 1.07 | 1.05 | 1.06 | 0.01 | 34.34 | 5.33 | Tnnt2 | 24837 | XP_006249900 | 564380662 | -1.623 | 1.019 | 1 |  | | | | | | | | | | | | | | | | | |
| D3ZH41 | Cytoskeleton-associated protein 4 (Predicted) | 33.77 | 34.97 | 11 | 0.96 | 1.03 | 1.13 | 1.16 | 1.07 | 0.09 | 36.29 | 4.82 | Ckap4 | 362859 | NP_001102210 | 157823877 | -0.503 | 0.386 | 15 |  | | | | | | | | | | | | | | | | | |
| G3V6P7 | Myosin, heavy polypeptide 9, non-muscle | 22.34 | 6.33 | 10 | 1.16 | 1.04 | 1.15 | 1.03 | 1.1 | 0.07 | 226.3 | 5.66 | LOC100911597 | 100911597 | XP_003750426 | 392349606 | -0.84 | 0.049 | 1,2 |  | | | | | | | | | | | | | | | | | |
| Q4G069 | Regulator of microtubule dynamics protein 1 | 5.84 | 6.13 | 2 |  |  | 1.23 | 1.08 | 1.16 |  | 35.38 | 7.74 | Rmdn1 | 500419 | Q4G069 | 123789064 | -0.293 | 0.057 |  |  | | | | | | | | | | | | | | | | | |
| D3ZCV0 | Protein Actn2 | 66.07 | 23.6 | 16 | 1.14 | 1.14 | 1.1 | 1.24 | 1.16 | 0.06 | 103.8 | 5.45 | Actn2 | 291245 | NP_001163796 | 281332157 | -0.605 | 0.212 |  |  | | | | | | | | | | | | | | | | | |
| D3ZX18 | Myozenin 2 (Predicted), isoform CRA_b | 6.27 | 17.84 | 4 | 1.06 | 1.29 | 1.15 | 1.2 | 1.18 | 0.1 | 20.58 | 5.02 | Myoz2 | 295426 | NP_001099939 | 157816913 | -0.711 | 0.243 |  |  | | | | | | | | | | | | | | | | | |
| G3V7K1 | Myomesin 2 | 235.4 | 37.98 | 45 | 1.24 | 1.21 | 1.15 | 1.25 | 1.21 | 0.04 | 164.6 | 5.88 | Myom2 | 306616 | NP_001162612 | 281306803 | -0.467 | 0.492 | 1 |  | | | | | | | | | | | | | | | | | |
| P04692 | Tropomyosin alpha-1 chain | 149.4 | 71.48 | 29 | 1.24 | 1.2 | 1.26 | 1.15 | 1.21 | 0.05 | 32.66 | 4.74 | Tpm1 | 24851 | P04692 | 92090646 | -1.042 | 2.266 | 1 |  | | | | | | | | | | | | | | | | | |
| P08733 | Myosin regulatory light chain 2, ventricular/cardiac muscle isoform | 132.6 | 84.94 | 15 | 1.29 | 1.22 | 1.3 | 1.25 | 1.25 | 0.04 | 18.87 | 4.93 | Myl2 | 363925 | P08733 | 127167 | -0.59 | 2.385 | 1 |  | | | | | | | | | | | | | | | | | |
| P16409 | Myosin light chain 3 | 163.7 | 67 | 14 | 1.37 | 1.22 | 1.32 | 1.32 | 1.31 | 0.06 | 22.14 | 5.1 | Myl3 | 24585 | P16409 | 127151 | -0.634 | 2.529 | 1 |  | | | | | | | | | | | | | | | | | |
| P23693 | Troponin I, cardiac muscle | 54.62 | 39.81 | 8 | 1.31 | 1.36 | 1.31 | 1.26 | 1.31 | 0.04 | 24.14 | 9.55 | Tnni3 | 29248 | P23693 | 136215 | -0.935 | 1.035 | 1 |  | | | | | | | | | | | | | | | | | |
| Q4PP99 | Cardiac troponin C | 32.89 | 29.81 | 5 | 1.53 | 1.09 | 1.5 | 1.22 | 1.34 | 0.22 | 18.41 | 4.18 | Tnnc1 | 290561 | Q4PP99 | 81918069 | -0.627 | 0.652 |  |  | | | | | | | | | | | | | | | | | |
| G3V8B0 | Myosin-7 | 1166 | 62.02 | 46 | 1.57 | 1.45 | 1.51 | 1.44 | 1.49 | 0.06 | 222.8 | 5.76 | Myh7 | 29557 | XP_006252013 | 564386123 | -0.792 | 1.841 |  |  | | | | | | | | | | | | | | | | | |
| Q9QZ76 | Myoglobin | 201.2 | 74.03 | 19 | 1.63 | 1.4 | 1.57 | 1.49 | 1.52 | 0.1 | 17.15 | 8.1 | Mb | 59108 | Q9QZ76 | 78099013 | -0.373 | 4.607 | 1 |  | | | | | | | | | | | | | | | | | |

*Chaperon proteins.

†Lipid droplet proteins were extracted from rat hearts. The whole lipid droplet proteins were subjected to LC-MS/MS analysis after iTRAQ labeling.

Note: GI No., GI number; Uniprot, Uniprot Accessing number; Seq. Cov., sequence coverage; MW, molecular weight; pI, isoelectricpoint; GRAVY, grand average of hydropath; PAF, protein abundance factor.

**Reference**

1. Zhang, H. *et al.* Proteome of skeletal muscle lipid droplet reveals association with mitochondria and apolipoprotein a-I. *J Proteome Res* **10**, 4757-4768 (2011).

2. Kim, S. C. *et al.* A clean, more efficient method for in-solution digestion of protein mixtures without detergent or urea. *J Proteome Res* **5**, 3446-3452 (2006).

3. Binns, D. *et al.* An intimate collaboration between peroxisomes and lipid bodies. *J Cell Biol* **173**, 719-731 (2006).

4. Umlauf, E. *et al.* Association of stomatin with lipid bodies. *J Biol Chem* **279**, 23699-23709 (2004).

5. Beller, M. *et al.* Characterization of the Drosophila lipid droplet subproteome. *Mol Cell Proteomics* **5**, 1082-1094 (2006).

6. Liu, P. *et al.* Chinese hamster ovary K2 cell lipid droplets appear to be metabolic organelles involved in membrane traffic. *J Biol Chem* **279**, 3787-3792 (2004).

7. Bartz, R. *et al.* Dynamic activity of lipid droplets: protein phosphorylation and GTP-mediated protein translocation. *J Proteome Res* **6**, 3256-3265 (2007).

8. Turro, S. *et al.* Identification and characterization of associated with lipid droplet protein 1: A novel membrane-associated protein that resides on hepatic lipid droplets. *Traffic* **7**, 1254-1269 (2006).

9. Athenstaedt, K., Zweytick, D., Jandrositz, A., Kohlwein, S. D. & Daum, G. Identification and characterization of major lipid particle proteins of the yeast Saccharomyces cerevisiae. *J Bacteriol* **181**, 6441-6448 (1999).

10. Fujimoto, Y. *et al.* Identification of major proteins in the lipid droplet-enriched fraction isolated from the human hepatocyte cell line HuH7. *Biochim Biophys Acta* **1644**, 47-59 (2004).

11. Katavic, V., Agrawal, G. K., Hajduch, M., Harris, S. L. & Thelen, J. J. Protein and lipid composition analysis of oil bodies from two Brassica napus cultivars. *Proteomics* **6**, 4586-4598 (2006).

12. Brasaemle, D. L., Dolios, G., Shapiro, L. & Wang, R. Proteomic analysis of proteins associated with lipid droplets of basal and lipolytically stimulated 3T3-L1 adipocytes. *J Biol Chem* **279**, 46835-46842 (2004).

13. Sato, S. *et al.* Proteomic profiling of lipid droplet proteins in hepatoma cell lines expressing hepatitis C virus core protein. *J Biochem* **139**, 921-930 (2006).

14. Wu, C. C., Howell, K. E., Neville, M. C., Yates, J. R., 3rd & McManaman, J. L. Proteomics reveal a link between the endoplasmic reticulum and lipid secretory mechanisms in mammary epithelial cells. *Electrophoresis* **21**, 3470-3482 (2000).

15. Wan, H. C., Melo, R. C., Jin, Z., Dvorak, A. M. & Weller, P. F. Roles and origins of leukocyte lipid bodies: proteomic and ultrastructural studies. *FASEB J* **21**, 167-178 (2007).

16. Cermelli, S., Guo, Y., Gross, S. P. & Welte, M. A. The lipid-droplet proteome reveals that droplets are a protein-storage depot. *Curr Biol* **16**, 1783-1795 (2006).
